# An Alumanyl Test Case of Group 1 Redox Interchange

**DOI:** 10.1002/chem.202502197

**Published:** 2025-07-21

**Authors:** Kyle G. Pearce, Agustín Morales, Michael S. Hill, Claire L. McMullin

**Keywords:** alumanyl, cesium, density functional theory, lithium, potassium, rubidium, sodium

## Abstract

Rationalized by a thermochemical evaluation using both theoretical density functional theory (DFT) and empirical data, we show that the arene‐encapsulated M^+^ cations of the group 1 alumanyls, [{SiN^Dipp^}AlM]_2_ ({SiN^Dipp^} = {CH_2_SiMe_2_N(Dipp)}_2_; Dipp = 2,6‐*i*‐Pr_2_C_6_H_3_); M = Li, Na, K, Rb, Cs] may be interconverted by redox reactions with the elemental alkali metals. These observations contradict the established *E* ^0^ data and allow access to an otherwise inaccessible sodium derivative.

## Graphical abstract

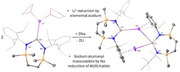

## 1 Introduction

**Figure 1:**
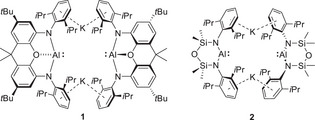
The structures of compounds **1** and **2**.

Notwithstanding Dye and co‐workers’ identification of alkalide (M^−^; M = Na, K, Rb, Cs) anions,^[^ ^1^ ^]^ little consideration is applied to the redox behavior of group 1 elements in molecular systems. Although the synthesis of many low oxidation state *p*‐block compounds,^[^ ^2^ ^]^ and even group 2 derivatives,^[^ ^3^ ^]^ requires reduction of a higher oxidation state precursor, the choice of alkali metal is more often dictated by synthetic expedience than consideration of the exact source of reducing electrons. In such cases, the resultant M^+^ cation is either a component of an ionic salt by‐product or, if incorporated into the target compound, most commonly viewed as an innocent bystander to the now reduced element of interest. A relevant case in point is the development of diamidoalumanyl derivatives (e.g., **1** and **2**, Figure 1),^[^ ^4^ ^]^ where charge balance of the Al(I) anions has most commonly been maintained by K^+^ after potassium reduction of an aluminum(III) iodide.

**Scheme 1:**
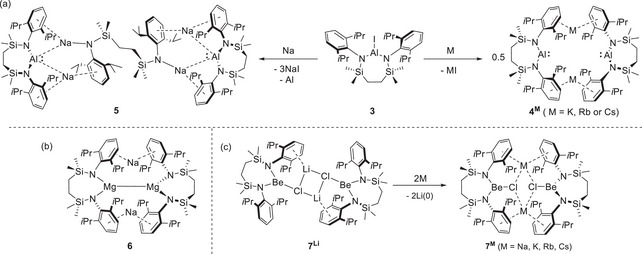
a) Alkali metal reduction of **3** to provide **4^K^**, **4^Rb^**, **4^Cs^,** and **5**; b) the structure of **6**; c) the synthesis of compounds **7** **^M^** by alkali metal (M = Na, K, Rb, Cs) reduction of **7^Li^**.

Whereas changes to the identity of M^+^ have been recognized to impact on the solid‐ and/or solution‐state structures of contact ion pair group 1 alumanyls like **1** and **2**,^[^ ^5^ ^]^ the consequences of such variations on reactivity are less regularly considered.^[^ ^5a^ ^]^ Our own studies have focused on the reduction of the aluminum(III) iodide, [{SiN^Dipp^}AlI] (**3**, {SiN^Dipp^}^2−^ = {CH_2_SiMe_2_NDipp}_2_^2−^ where Dipp = 2,6‐*i*‐Pr_2_C_6_H_3_; Scheme 1a). Reactions of **3** with potassium, rubidium, or cesium, provided the contact ion pair derivatives, [{SiN^Dipp^}AlM]_2_ [**4** **^M^**; M = K (**4^K^**);^[^ ^4^, ^6^ ^]^ Rb (**4^Rb^**); Cs (**4^Cs^**)].^[^ ^5c^ ^]^ The facility of these species toward oxidative addition of both arene (C(*sp* ^2^)) and terminal alkyne (C(*sp*)) C‐H bonds was found to be dependent on the constituent alkali metal.^[^ ^5^, ^7^ ^]^ Attempted extension to a lighter sodium analogue (**4^Na^**) was frustrated by the facile over‐reduction of **3**. This was evident even at the first point of analysis, with the incorporation of a formal equivalent of [{SiN^Dipp^}Na_2_] into the resultant sodium alumanyl species (**5**, Scheme 1a).

Electrochemical reduction potentials provide a commonly used figure of merit to assess the reductive potency of the individual group 1 metals.^[^ ^8^ ^]^ As demonstrated by their use in the low oxidation state group 13 chemistry shown in Scheme 1a, the data for the five available alkali metals are all highly negative [i.e., M_(aq)_^+^ + e^−^ → M_(s)_; where M = Li (*E* ^0^ = −3.04 V vs. SHE), Na (−2.71 V), K (−2.93 V), Rb (−2.98 V), Cs (−2.92 V)].^[^ ^8^ ^]^ When viewed in isolation, these values describe the relative thermodynamic resistance to reduction of all the group 1 elements in their monocationic form and imply that the Li^+^ cation should be impervious to its chemical reduction. It should be noted, however, that these data are experimentally determined under standard/aqueous conditions. The electrochemical assessment is, thus, heavily biased by the M^+^ hydration enthalpies (Δ*H*_Hyd_), the values of which decline incrementally as group 1 is descended (Li^+^ 506; Na^+^ 406; K^+^ 330; Rb^+^ 310; Cs^+^ 276 kJ mol^−1^).^[^ ^8b^ ^]^

Contradicting this naïve expectation, a number of recent reports have highlighted that chemical reduction of M^+^, and even Li^+^, may be achieved with a highly reducing reaction medium in conjunction with a coordination environment provided by the M^+^ cation's molecular encapsulation.^[^ ^3^, ^9^ ^]^ The heterobimetallic compound, [{SiN^Dipp^}MgNa]_2_ (**6**, Scheme 1b), for example, extrudes a pure sodium mirror when treated with bases such as THF, a process deduced to be initiated by intramolecular [2Na^+^ + {Mg‐Mg}^2+^ → 2Na(0) + 2Mg^2+^] electron transfer through ether coordination and disruption of the otherwise stabilizing Na^+^∙∙∙arene interactions.^[^ ^9d^ ^]^

The current work is prompted by our attempted synthesis of a beryllium analogue of compound **6** by reduction of the dimeric lithium chloroberyllate, [{SiN^Dipp^}BeClLi]_2_ (**7^Li^**), with either sodium, potassium, rubidium, or cesium metal.^[^ ^10^, ^11^ ^]^ Rather than Be^2+^ reduction, these reactions resulted in exclusive replacement of the lithium cations to provide [{SiN^Dipp^}BeClM]_2_ (**7** **^M^** where M = Na, K, Rb, Cs, Scheme 1c). This outcome was rationalized by construction of thermodynamic Hess's law cycles derived from a combination of density functional theory (DFT) calculation energies and the free energies of atomization (Δ*G*_at_) of each alkali metal in their bulk forms. Incorporation of these latter inputs, deduced from the available enthalpies of atomization (Δ*H*_at_) and solid‐state and gas‐phase entropies,^[^ ^12^ ^]^ yielded data consistent with the exergonicity inferred from the experimental observations. Further validation of this approach was provided by the energetics associated with the transformation of **7^K^** to **7^Rb^** (ΔΔ*G* = −0.5 kcal mol^−1^), the isogonic nature of which was experimentally verified by the reversible interconversion of both species. In a further examination of this approach, we have reported that a charge‐separated 12‐crown‐4 (12‐cr‐4) derivative of **7^Li^** may be similarly converted by sodium and potassium metal reduction to the heavier congeneric analogues, [{SiN^Dipp^}BeCl]^−^[M(12‐cr‐4)_2_]^+^ (M = Na and K), albeit the broader range of transformation provided by the M∙∙∙arene encapsulation provided by **7** **^M^** was perturbed by the crown ether coordination environment.^[^ ^13^ ^]^

The behavior of the arene‐coordinated M^+^ centers of **7** **^M^** contradicts the established *E* ^0^ values. An immediate question arising, therefore, is whether analogous group 1 redox interchange can be observed in other molecular systems featuring similar M^+^‐arene encapsulation. In this contribution and prompted by the notable stability of the Al(I) centers of diamidoalumanyls such as **1** and **2**, we establish that [{SiN^Dipp^}AlM]_2_ (**4** **^M^,** Scheme 1a) also provides suitable scaffolds for alkali metal (M^+^/Mʹ) interchange. Furthermore, through the synthesis of the sodium derivative **4^Na^**, we demonstrate that this approach circumvents the formation of compound **5** (Scheme 1a), providing a novel means to explore previously inaccessible chemical space.

Our study commenced with a theoretical/thermochemical assessment, similar to that applied to **7** **^M^**,^[^ ^11^ ^]^ of the potential for the group 1 alumanyls (**4** **^M^**) to undergo alkali metal redox exchange (see the Supporting Information). These results, which include the previously unreported lighter group 1 derivatives, **4^Li^** and **4^Na^**, are summarized in Table 1 and indicate the thermodynamic viability of the sequential reduction of the alkali metal component of **4** **^M^** by its successively heavier group 1 congener. The reversibility of the exchange implied by the almost isoenergetic nature of **4^K^** and **4^Rb^** is again especially notable (entries 3 and 4, *vide infra*).

**Table 1:** Summary of Gibbs energies (ΔG(*sol&s*); *sol *= solution, *s* = solid) for redox interconversion of [{SiN^Dipp^}AlM]_2_ (**4** **^M^**) estimated from a combined experimental and computational Hess cycle using the methodology articulated in reference 11 and using the methodology described in the Supporting Information; BP86‐D3^BJ^(CPCM = C_6_H_6_)/BS3//BP86/BS1. Gibbs solvation energies were calculated using CPCM(C_6_H_6_) and BS3 = ZORA‐def2‐TZVPP and ZORA(‐SARC)‐TZVPP (for Rb and Cs).

Entry	Reaction	ΔG[*sol&s*] kcal mol^−1^
1	**4^Li^**_(sol)_ + 2Na_(s)_ **→ 4^Na^**_(sol)_ + 2Li_(s)_	−14.3
2	**4^Na^**_(sol)_ + 2K_(s)_ **→ 4^K^**_(sol)_ + 2Na_(s)_	−8.6
3	**4^K^**_(sol)_ + 2Rb_(s)_ **→ 4^Rb^**_(sol)_ + 2K_(s)_	−0.1
4	**4^Rb^**_(sol)_ + 2K_(s)_ **→ 4^K^**_(sol)_ + 2Rb_(s)_	0.1
5	**4^Rb^**_(sol)_ + 2Cs_(s)_ **→ 4^Cs^**_(sol)_ + 2Rb_(s)_	−3.1
6	**4^K^**_(sol)_ + 2Cs_(s)_ **→ 4^Cs^**_(sol)_ + 2K_(s)_	−3.2

Although compounds **4^K^**, **4^Rb^,** and **4^Cs^** were experimentally available by direct reduction of **3**, the synthesis of both lighter analogues, **4^Li^** and **4^Na^**, by a similar method has been more problematic. Sodium reduction of **3** results in the exclusive formation of compound **5** (Scheme 1a),^[^ ^5c^ ^]^ while, in unreported observations, all our attempts to access **4^Li^** by a similar route have been unsuccessful. Aldridge and Goicoechea have reported that treatment of the precursor to compound **1** (Figure 1), [{_Xanth_NON^Dipp^}AlI], with lithium metal also induced decomposition.^[^ ^14^ ^]^ In contrast, Coles and co‐workers found that the dimeric lithium analogue of compound **2**, [(NON)AlLi]_2_ (**8^Li^**), may be synthesized by direct lithium reduction of [{NON}AlI] ({NON}^2−^ = {O(SiMe_2_NDipp)_2_}^2−^).^[^ ^5a^ ^]^ The same study also showed that crystallization of **8^Li^** from Et_2_O afforded the Al‐Li bonded monomeric ion pair [{NON}AlLi(Et_2_O)_2_] (**9**). In an effectively simultaneous advance, Aldridge and co‐workers described the similarly monomeric lithium alumanyl, [(_Xanth_NON^Dipp^)AlLi(OEt_2_)_2_] (**10**), synthesized by Li^+^ for K^+^ exchange of **1** with LiI in diethyl ether.^[^ ^14^ ^]^

**Scheme 2:**
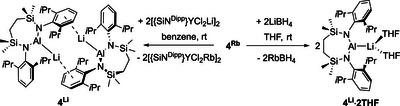
Synthesis of **4^Li^∙2THF** and **4^Li^**.

**Figure 2:**
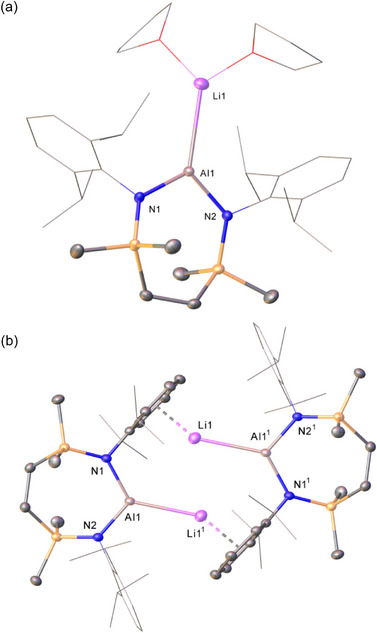
Molecular structures of a) compound **4^Li^·2THF** and b) compound **4^Li^** with displacement ellipsoids at 30%. For clarity, hydrogen and disordered atoms, and occluded benzene (**4^Li^**) are omitted. Similarly, Dipp groups not involved in π∙∙∙arene interactions and THF ligands are displayed as wireframe. Selected bond lengths (Å) and angles (°): (**4^Li^·2THF**) Al1‐N2 1.886(2), Al1‐N1 1.884(2), Al1‐Li1 2.710(6), Li1‐O1 1.891(6), Li1‐O2 1.910(6), N1‐Al1‐N2 109.11(10), N2‐Al1‐Li1 127.71(14), N1‐Al1‐Li1 123.10(14); (**4^Li^**) Al1‐N1 1.8956(19), Al1‐N2 1.873(2), Al1‐Li1^1^ 2.808(5), N1‐Al1‐Li1^1^ 129.08(12), N2‐Al1‐N1 110.54(9), N2‐Al1‐Li1^1^ 120.21(12). Symmetry operations to generate primed atoms: ^1^1‐*x*, 1‐*y*, 2‐*z*.

Directed by this latter report, compound **4^Rb^** was reacted with two stoichiometric equivalents of LiBH_4_ in THF (Scheme 2). Removal of volatiles and crystallization from hexane provided compound **4^Li^·2THF**, which formed exclusively and was characterized by NMR spectroscopy and single‐crystal X‐ray diffraction analysis (Figure 2a). In a further serendipitous advance, we observed that the reaction of compound **4^Rb^** with [{SiN^Dipp^}YCl_2_Li]_2_ provided base‐free **4^Li^** (Scheme 2). However unusual, the lithium dichloroyttrate reagent in this reaction performs a similar, albeit now benzene‐soluble, role to the borohydride anion employed in the synthesis of **4^Li^·2THF**. The polymeric rubidium salt, [{SiN^Dipp^}YCl_2_Rb]_∞_, then crystallizes readily from solution, allowing the isolation of dimeric **4^Li^** in a moderate yield (54%) of single crystals suitable for X‐ray diffraction analysis (Figure 2b) on further concentration of the filtrate.

Like Coles’ and Aldridge's previously described alumanyl lithium‐ether adducts,^[^ ^5^, ^14^ ^]^ **4^Li^·2THF** is a monomeric ion pair with an unsupported Al─Li bond [2.710(6) Å]. While this distance is marginally shorter than the equivalent interactions observed in compounds **9** [2.767(2) Å] and **10** [2.750(4) Å],^[^ ^5^, ^14^ ^]^ and in a tris‐THF ligated analogue of **9** [2.839(5) Å], it is slightly elongated in comparison to that of a further TMEDA adduct, [(NON)AlLi(TMEDA)] [2.669(5) Å], which also displays a terminal Al‐Li interaction.^[^ ^15^ ^]^ Irrespective of these minor variations, the constitution of **4^Li^·2THF** may be considered as typical of its structural type, precluding further necessary comment.

In a similar manner to Coles’ solvent‐free analogue (**8^Li^**),^[^ ^5a^ ^]^ the centrosymmetric dimer **4^Li^** is best viewed as adopting a “slipped” contact dimeric pair structure in which each lithium interacts directly with Al [Al1‐Li1^1^ 2.808(5) Å]. Dimerization is then effected by Li···π(aryl) interactions with a Dipp substituent of the adjacent alumanyl moiety [Li1∙∙∙Ct 2.008 Å]. Although the precedent is again very limited, this latter feature is commensurate with that observed for **8^Li^**, again obviating additional discussion.

**Scheme 3:**
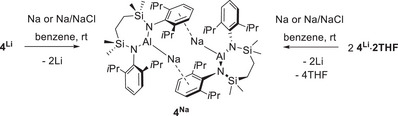
Synthesis of compound **4^Na^**.

**Figure 3:**
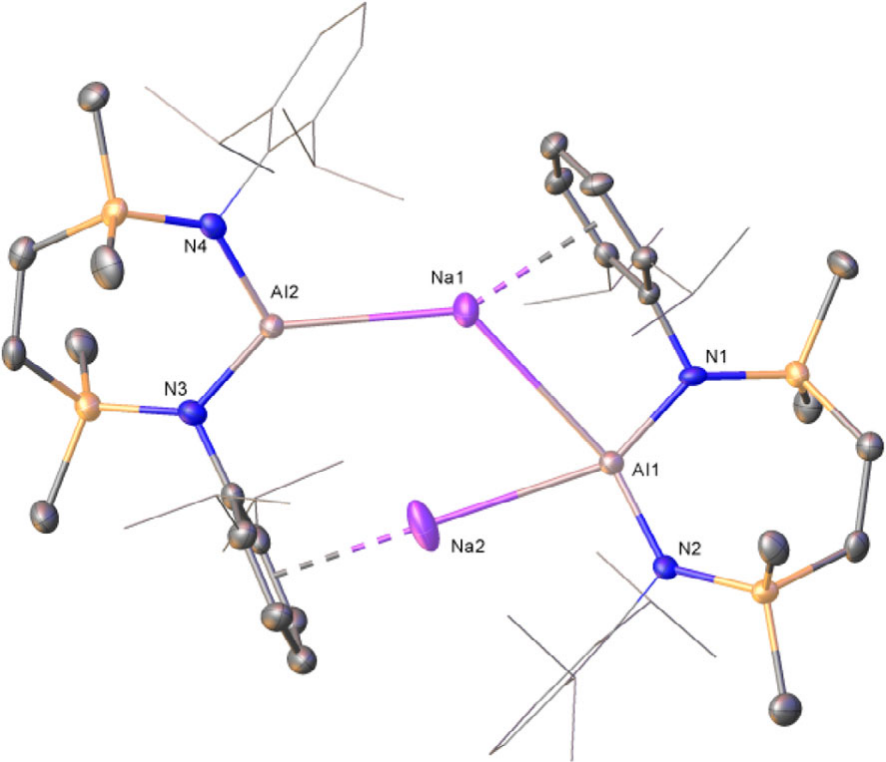
Molecular structure of compound **4^Na^** with displacement ellipsoids at 30%. For clarity, hydrogen and disordered atoms are omitted. Similarly, Dipp groups not involved in π∙∙∙arene interactions are displayed as wireframe. Selected bond lengths (Å) and angles (°): Al1‐Na1 3.316(6), Al1‐Na2 3.105(6), Al1‐N1 1.883(9), Al1‐N2 1.854(9), Al2‐Na1 3.048(5), Al2‐N3 1.894(10), Al2‐N4 1.888(10), N2‐Al1‐N1 110.1(4) N4‐Al2‐N3 109.9(4), N1‐Al1‐Na1 87.7(3), N1‐Al1‐Na2 150.8(3), N2‐Al1‐Na1 159.6(3), N2‐Al1‐Na2 98.7(3), N3‐Al2‐Na1 135.3(3), N4‐Al2‐Na1 114.8(3), Al2‐Na1‐Al1‐128.64(19).

With **4^Li^·2THF** and **4^Li^** in hand, reactions were performed in benzene between both compounds and either elemental sodium or Na/NaCl reductants (Scheme 3). Although sonication was required to induce reactions with the bulk metal, all four systems provided similar observations and the formation of a predominant new species (**4^Na^**). The formation of **4^Na^** was readily identified in solution by ^1^H NMR spectroscopy, which demonstrated a shift in the Dipp *iso*‐propyl methine signal from either δ 4.29 ppm (**4^Li^∙2THF**) or δ 3.74 ppm (**4^Li^**) to δ 3.84 ppm. Notably, no evidence for the formation of the previously reported species **5** (Scheme 1a) could be observed. Although compound **4^Na^** proved unusually sensitive, decomposing via an unidentified mechanism even on extended exposure to vacuum, filtration of the reaction of Na/NaCl and **4^Li^∙2THF** and slow evaporation of the reaction solvent provided a sample suitable for single crystal X‐ray diffraction analysis (88%). The results of this experiment were unambiguous and confirmed **4^Na^** as a further example of a “slipped” contact dimer, with a structure (Figure 3) that resembles both **4^Li^** and Coles and co‐workers’ [{NON}AlNa]_2_.^[^ ^5^, ^15^ ^]^ The alkali metal centers of **4^Na^** are encapsulated by a combination of shorter [Al1‐Na2 3.105(6), Al2‐Na1 3.048(5) Å] and longer [Al1‐Na1 3.316(6) Å] Al‐Na contacts augmented by robust Na∙∙∙arene interactions [Na1∙∙∙Ct 2.563, Na2∙∙∙Ct 2.570 Å] to the C9‐ and C38‐containing Dipp substituents, respectively. Although **4^Na^** lacks the inversion center of its literature precedent, these values are again commensurate with those provided by [{NON}AlNa]_2_ [Al‐Na 3.0305(6), Na∙∙∙Ct 2.4596(8) Å] and, thus, may be considered representative of this structural type.

In the light of these observations and the implications of the data presented in Table 1, the broader scope of this reductive protocol for the redox interconversion of the group 1 alumanyl derivatives was investigated. The instability of **4^Na^** mitigated against a confident assessment of its bulk transformation. Monitoring of a sample of **4^Li^** in benzene by ^1^H NMR spectroscopy, however, when treated either with Na/NaCl or sonicated with sodium metal to complete conversion to **4^Na^**, followed by filtration onto freshly cut potassium, resulted in stoichiometric transformation to **4^K^**. Similar reactions of **4^K^** with elemental rubidium or cesium, both on a bulk synthetic or NMR scale, resulted in the isolation of the previously described **4^Rb^** and **4^Cs^** in high yield (>90%).^[^ ^5c^ ^]^

**Scheme 4:**
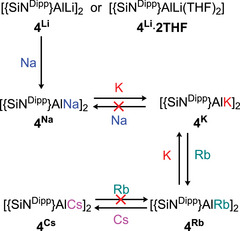
Observed scope of the group 1 redox reactivity of **4** **^M^**.

Analogous conversion to **4^Cs^** was also achieved by reduction of **4^Rb^** with elemental cesium. In line with the expectation provided by their thermochemical estimates (entries 3 and 4, Table 1), and our previous observations of **7** **^M^** (Scheme 1c),^[^ ^11^ ^]^ only **4^K^** and **4^Rb^** could be mutually interconverted by reactions with rubidium or potassium metal. The various alkali metal‐based transformations described in this contribution are, thus, summarized in Scheme 4.

In conclusion, we present experimental verification of alkali metal redox interchange within a series of group 1 alumanyls (**4** **^M^**). The synthetic observations further validate a predictive DFT/thermochemical evaluation and, in this instance, also provide a synthesis of the previously inaccessible sodium analogue, **4^Na^**. In conjunction with the analogous behavior of [{SiN^Dipp^}BeClM]_2_,^[^ ^11^ ^]^ these observations suggest that M^+^/Mʹ(0) interchange may be extended to other systems that feature similar M^+^ arene encapsulation and signpost potential as a previously unrecognized protocol in *s*‐block element synthesis. More provocatively, these observations again contradict the expectation prompted by standard electrochemical data and imply that subtle “ligand field” considerations may be more broadly applied in the molecular chemistry of the alkali metals. We are continuing to explore the potential of this reactivity to allow access to a greater diversity of group 1‐containing species.

## Supporting Information

Full experimental and instrumental details, spectral data, NMR spectra, details of the X‐ray analysis and the methods employed in the quantum chemical investigations of this chemistry are available in the Supporting Information to this article. Crystallographic data for all compounds have been deposited with the Cambridge Crystallographic Data Centre as supplementary publications CCDC 2452423–2452427. Copies of these data can be obtained free of charge on application to CCDC, 12 Union Road, Cambridge CB2 1EZ, UK [fax(+44) 1223 336 033, e‐mail: deposit@ccdc.cam.ac.uk.

## Conflict of Interests

The authors declare no conflict of interest.

## Supporting information



Supporting Information

Supporting Information

## Data Availability

The data that support the findings of this study are available in the supplementary material of this article.
