# Supplementary material for: An Alumanyl Test Case of Group 1 Redox Interchange
Source: Chemistry. 2025 Jul 21;31(44):e202502197. doi: 10.1002/chem.202502197 (PMC12336768; doi:10.1002/chem.202502197)
Supplement: Supplementary file 1 — Supporting Information [file CHEM-31-e202502197-s001.pdf]

Synthetic Details

General Considerations

All manipulations were carried out using standard Schlenk line and glovebox techniques under an inert atmosphere of argon. NMR experiments were conducted in J-Young's NMR tubes and prepared in a glovebox. NMR spectra were recorded on a Bruker BioSpin GmbH spectrometer operating at 400.15 MHz (^{1}H) and 100.62 MHz (^{13}C) or on an Agilent ProPulse spectrometer operating at 194.3 MHz (^{7}Li). Elemental analyses were performed by the Elemental Analysis Services Team at London Metropolitan University. Solvents were dried by passage through a commercially available solvent purification system and stored under argon in ampoules over 4 Å molecular sieves. C_6D_6 and C_4D_8O were purchased from Merck, dried over potassium, distilled and stored over molecular sieves. $LiBH_4$ and YCl_3 were purchased from Merck and used without purification. $[\{CH_2SiMe_2NDipp\}_2AlK]_2$[1,2] and 10% w/w Na/NaCl were synthesised according to literature procedures.[3]

Synthesis of $[\{CH_2SiMe_2NDipp\}_2AlLi(THF)_2]$ (4-Li·2THF)

$[\{CH_2SiMe_2NDipp\}_2AlRb]_2$ (**4^{Rb},** 0.65 g, 0.5 mmol) and $LiBH_4$ (24 mg, 1.07 mmol) were introduced into an ampoule, THF (*ca.* 10 cm^3) was added, and the mixture was stirred for 2 hours. The solution was filtered away from the colorless precipitate and volatiles were removed *in vacuo*. The product was extracted into hexane and filtered into a fresh ampoule. The solution was decanted into a vial and placed in the freezer (–35 °C), affording colourless crystals overnight. Crystal yield: 0.503 g, 73 %.

^{1}H NMR (C_6D_6): δ = 7.09 (d, Dipp-*m*-Ar-H, $^{3}J_{HH}$ = 7.53 Hz, 4H), 6.94 (t, Dipp-*p*-Ar-H, $^{3}J_{HH}$ = 7.53 Hz, 2H), 4.29 (sept., $C\underline{H}(CH_3)_2$, $^{3}J_{HH}$ = 6.93 Hz, 4H), 3.02 ($O\{C\underline{H}_2CH_2\}_2$), 1.46 (d, $CH(C\underline{H}_3)_2$, $^{3}J_{HH}$ = 6.93 Hz, 12H), 1.31 (overlapping $CH(C\underline{H}_3)_2$ and $SiCH_2$, 16H), 1.23 ($O\{CH_2C\underline{H}_2\}_2$), 0.36 (s, $SiCH_3$, $^{2}J_{HSi}$ = 54.9 Hz, 12H).

$^{13}C\{^{1}H\}$ NMR (C_6D_6): δ = 149.4 (*i*-C_6H_3), 147.8 (*o*-C_6H_3), 122.9 (*m*-C_6H_3), 121.8 (*p*-C_6H_3), 68.0 ($O\{\underline{C}H_2CH_2\}_2$), 28.0 ($\underline{C}H(CH_3)_2$), 25.9 ($CH(\underline{C}H_3)_2$), 25.4 ($O\{CH_2\underline{C}H_2\}_2$), 24.5 ($CH(\underline{C}H_3)_2$), 14.8 ($SiCH_2$), 1.72 ($SiCH_3$)

^{7}Li NMR (C_6D_6) δ = 0.13 (s).

Anal. Calc. for Calc. for $C_{38}H_{64}AlLiN_2O_2Si_2$: C, 68.02; H, 9.61; N, 4.17. Found: C, 66.73; H, 9.70; N, 3.88.

Synthesis of $[\{CH_2SiMe_2NDipp\}_2YCl_2Li]_2$

^{n}Butyl lithium (2.5 M, 1.61 cm^3, 4.02 mmol) was slowly added dropwise to a pre-cooled (0 °C) ethereal solution (*ca.* 20 cm^3) of $\{CH_2SiMe_2N(H)Dipp\}_2$ (1 g, 2.01 mmol), resulting in a colourless suspension. The reaction mixture was stirred for 30 minutes at 0 °C, before being allowed to warm to ambient temperature and stir for a further 120 minutes. The suspension was then slowly added to an ethereal solution of YCl_3 (0.393 g, 2.01 mmol), and the reaction mixture was left to stir for 16 hours. After

filtration, the volatiles were removed under reduced pressure, the crude solid was then extracted into hexane and concentrated, before being washed with hexane at –78 °C. The colorless solid was then dried under reduced pressure. Yield: 0.62 g, 47%.

^{1}H NMR (C_6D_6): δ = 7.18-7.03 (m, Ar-H, 4H), 6.99 (t, Dipp-*p*-H, $^{3}J_{HH}$ = 7.63 Hz, 2H), 3.92 (sept., $C\underline{H}(CH_3)_2$, $^{3}J_{HH}$ = 6.74 Hz, 4H), 1.58 (d, $CH(C\underline{H}_3)_2$, $^{3}J_{HH}$ = 6.74 Hz, 12H), 1.40 (s, $SiCH_2$, 4H), 1.36 ($CH(C\underline{H}_3)_2$, $^{3}J_{HH}$ = 6.74 Hz, 12H), 0.39 (s, $SiCH_3$, 12H).

Synthesis of [{CH_2SiMe_2NDipp}$_2$AlLi]$_2$ (4^{Li})

[{CH_2SiMe_2NDipp}$_2$AlRb]$_2$ (**4^{Rb}**, 21 mg, 0.017 mmol) and [{CH_2SiMe_2NDipp}$_2YCl_2Li$]$_2$ (22.88 mg, 0.017 mmol) were introduced into a J-Young's NMR tube and dissolved in C_6D_6 (0.6 cm^3), resulting in the deposition of colourless crystals of [{CH_2SiMe_2NDipp}$_2YCl_2Rb$]$_n$ over the course of 4 hours, these colourless crystals were isolated, washed with cold hexane and dried under reduced pressure. Yield: 22.9 mg, 91%. The initial C_6D_6 supernatant was concentrated to incipient crystallisation and left to sit overnight to afford pale yellow crystals of [{CH_2SiMe_2NDipp}$_2$AlLi]$_2$, which were washed with cold hexane. Yield: 9.71 mg, 54%.

[{CH_2SiMe_2NDipp}$_2$AlLi]$_2$ (4^{Li}): ^{1}H NMR (C_6D_6): δ = 7.06-6.94 (m, Dipp-*m*-Ar-H, 4H), 6.55 (t, Dipp-*p*-Ar-H, $^{3}J_{HH}$ = 7.43 Hz, 2H), 3.74 (sept., $C\underline{H}(CH_3)_2$, $^{3}J_{HH}$ = 6.74 Hz, 4H), 1.23 (d, $CH(C\underline{H}_3)_2$, $^{3}J_{HH}$ = 6.74 Hz, 12H), 1.16 (d, $CH(C\underline{H}_3)_2$, $^{3}J_{HH}$ = 6.74 Hz, 12H), 1.03 (s, $SiC\underline{H}_2$, 4H), 0.19 (s, $SiCH_3$, 12H).

^{13}C{^{1}H} NMR (C_6D_6): δ = 148.2 (*i*-C_6H_3), 146.2 (*o*-C_6H_3), 123.6 (*m*-C_6H_3), 122.1 (*p*-C_6H_3), 28.4 ($\underline{C}H(CH_3)_2$), 25.4 ($CH(\underline{C}H_3)_2$), 23.8 ($CH(\underline{C}H_3)_2$), 14.4 ($SiCH_2$), 1.43 ($SiCH_3$).

[{CH_2SiMe_2NDipp}$_2YCl_2Rb$]$_n$: ^{1}H NMR (C_4D_8O): δ = 6.92 (d, Dipp-*m*-Ar-H, $^{3}J_{HH}$ = 7.68 Hz, 4H), 6.71 (t, Dipp-*p*-Ar-H, $^{3}J_{HH}$ = 7.68 Hz, 2H), 4.12 (sept., $C\underline{H}(CH_3)_2$, $^{3}J_{HH}$ = 6.78 Hz, 4H), 1.34 (s, $SiC\underline{H}_2$, 4H), 1.24 (d, $CH(C\underline{H}_3)_2$, $^{3}J_{HH}$ = 6.78 Hz, 12H), 1.15 (d, $CH(C\underline{H}_3)_2$, $^{3}J_{HH}$ = 6.78 Hz, 12H), 0.0 (s, $SiCH_3$, 12H).

^{13}C{^{1}H} NMR (C_4D_8O): δ = 151.7 (*i*-C_6H_3), 146.2 (*o*-C_6H_3), 123.7 (*m*-C_6H_3), 120.7 (*p*-C_6H_3), 27.8 ($\underline{C}H(CH_3)_2$), 27.0 ($CH(\underline{C}H_3)_2$), 24.3 ($CH(\underline{C}H_3)_2$), 13.7 ($SiCH_2$), 2.8 ($SiCH_3$)

Anal. Calc. for $C_{60}H_{100}Cl_4N_4Rb_2Si_4Y_2$: C, 48.68; H, 6.81; N, 3.78. Found: C, 47.46; H, 6.51; N, 3.44.

Synthesis of [{CH_2SiMe_2NDipp}$_2$AlNa]$_2$

[{CH_2SiMe_2NDipp}$_2$AlLi(THF)$_2$] (0.19 g, 0.28 mmol) and 10% w/w Na/NaCl (524 mg) were introduced into an ampoule, benzene (*ca.* 5 cm^3) was added, and the mixture was stirred for 2 hours. The solution was filtered away from the black solid and the solution was frozen with salt water. The volatiles were slowly removed *in vacuo*, affording a pale yellow fluffy solid. Yield: 137 mg, 88%.

The product was isolated and analysed by ^{1}H NMR spectroscopy (**Figure S19**), observing [{CH_2SiMe_2NDipp}$_2$AlNa]$_2$. In contrast, after prolonged exposure to vacuum (in salt ice water), only {CH_2SiMe_2N(H)Dipp}$_2$ could be observed spectroscopically (**Figure S21**).

^1H NMR (C_6D_6): δ = 7.09-6.85 (m, Dipp-Ar-H, 6H), 3.84 (sept., C<u>H</u>(CH_3)$_2$, $^3J_{HH}$ = 6.90 Hz, 4H), 1.28 (d, CH(C<u>H</u>$_3$)$_2$, $^3J_{HH}$ = 6.90 Hz, 12H), 1.10 (overlapping CH(C<u>H</u>$_3$)$_2$ and $SiCH_2$, 16H), 0.21 (s, $SiCH_3$, 12H).

$^{13}C\{^1H\}$ NMR (C_6D_6): δ = 148.4 (*i*-C_6H_3), 148.1 (*o*-C_6H_3), 123.4 (*m*-C_6H_3), 122.9 (*p*-C_6H_3), 27.9 (<u>C</u>H(CH_3)$_2$), 25.8 (CH(<u>C</u>H$_3$)$_2$), 24.2 (CH(<u>C</u>H$_3$)$_2$), 14.3 ($SiCH_2$), 1.50 ($SiCH_3$).

149.4 (*i*-C_6H_3), 147.8 (*o*-C_6H_3), 122.9 (*m*-C_6H_3), 121.8 (*p*-C_6H_3), 68.0 (O{<u>C</u>H$_2$CH$_2$}$_2$), 28.0 (<u>C</u>H(CH_3)$_2$), 25.9 (CH(<u>C</u>H$_3$)$_2$), 25.4 (O{CH$_2$<u>C</u>H$_2$}$_2$), 24.5 (CH(<u>C</u>H$_3$)$_2$), 14.8 ($SiCH_2$), 1.72 ($SiCH_3$)

Anal. Calc. for $C_{60}H_{100}Al_2N_4Na_2Si_4$: C, 66.13; H, 9.25; N, 5.14. Found: C, 65.43; H, 9.64; N, 4.12.

Notes: This synthesis can be achieved from either sodium metal or Na/NaCl starting from [{CH_2SiMe_2NDipp}$_2$AlLi(THF)$_2$] (**4^{Li}·2THF)** or [{CH_2SiMe_2NDipp}$_2$AlLi]$_2$ (**4^{Li}**) though due to the hard nature of sodium metal, long sonication times were required (4 x 40 minutes) resulting in some formation of the ring closed silazane, ({CH_2SiMe_2}$_2$NDipp; **Figure S24**). 10% w/w Na/NaCl was, thus, our preferred sodium source when performing this reaction on scale. Neither **4^{Li}·2THF or 4^{Li}** display any reactivity to NaCl alone.

This compound decomposes under exposure to vacuum, ultimately affording {CH_2SiMe_2N(H)Dipp}$_2$. Therefore, lyophilisation (freeze-drying) of this compound was the best method to overcome deleterious reaction pathways.

Synthesis of [{CH_2SiMe_2NDipp}$_2$AlRb]$_2$ (4^{Rb})

Rb metal was added to a C_6D_6 (0.6 cm^3) solution of [{CH_2SiMe_2NDipp}$_2$AlK]$_2$ (**4^K**, 20 mg, 0.04 mmol) inside a J. Young's NMR tube and sonicated for 20 minutes, providing a grey precipitate and allowing the spectroscopic identification of [{CH_2SiMe_2NDipp}$_2$AlRb]$_2$ (**4^{Rb}**). The solution was filtered away and volatiles were removed *in vacuo*, affording (**4^{Rb}**) as a yellow powder. Yield: 20.8 mg, 96%.

The spectroscopic signatures of **4^{Rb}** are consistent with the literature data.[2]

Synthesis of [{CH_2SiMe_2NDipp}$_2$AlCs]$_2$ (4^{Cs})

Cs metal was added to a C_6D_6 (0.6 cm^3) solution of [{CH_2SiMe_2NDipp}$_2$AlK]$_2$ (**4^K**, 20 mg, 0.04 mmol) inside a J. Young's NMR tube and sonicated for 3.5 minutes, providing a grey precipitate and the spectroscopic observation of [{CH_2SiMe_2NDipp}$_2$AlCs]$_2$ (**4^{Cs}**) as the exclusive product. The solution was filtered away from the grey precipitate and volatiles were removed *in vacuo*, affording [{CH_2SiMe_2NDipp}$_2$AlCs]$_2$ (**4^{Cs}**) as a yellow powder. Yield: 20.5 mg, 88%.

The spectroscopic signatures of **4^{Cs}** are consistent with the literature.[3]

Gram Scale Synthesis of [{CH_2SiMe_2NDipp}$_2$AlRb]$_2$ (4^{Rb})

Rb metal (350 mg, 4 mmol), [{CH_2SiMe_2NDipp}$_2$AlK]$_2$ (**4^K,** 20 mg, 0.04 mmol) and hexane (*ca.* 5 cm^3) were introduced into an ampoule and stirred at 40 °C for 16 hours. The yellow solution was filtered away from the grey precipitate, and the precipitate was washed with hexane. The combined hexane

extracts were concentrated under reduced pressure affording a yellow powder of **4^{Rb}**. Yield: 1.01 g, 94%.

Sequential Reduction of [{CH_2SiMe_2NDipp}$_2$AlLi]$_2$ (4^{Li})

[{CH_2SiMe_2NDipp}$_2$AlLi]$_2$ (**4^{Li}**, 20 mg, 0.04 mmol) was dissolved in C_6D_6 (0.6 cm^3), Na metal was added and the reaction mixture was sonicated for 4 $\times$ 40 minutes, observing the formation of [{CH_2SiMe_2NDipp}$_2$AlNa]$_2$ (**4^{Na}**), though the harsh conditions led to a small amount of {CH_2SiMe_2}$_2$NDipp formation, which is not observed when reduction is performed with 10% w/w Na/NaCl. The solution was then filtered into a J-Young's tube containing finely cut K metal and sonicated for 20 minutes observing the exclusive formation of [{CH_2SiMe_2NDipp}$_2$AlK]$_2$ (**4^{K}**).

Sequential Reduction of [{CH_2SiMe_2NDipp}$_2$AlK]$_2$ (4^{K})

[{CH_2SiMe_2NDipp}$_2$AlK]$_2$ (**4^{K}**, 20 mg, 0.04 mmol) was dissolved in C_6D_6 (0.6 cm^3), Na metal was added and the reaction mixture was sonicated for 1 hour with no observed reaction. The solution was filtered into a fresh J. Young's NMR tube containing Rb metal and sonicated for 20 minutes, affording [{CH_2SiMe_2NDipp}$_2$AlRb]$_2$ (**4^{Rb}**). The solution of **4^{Rb}** was filtered into a J. Young's tube containing finely cut K metal and was subsequently sonicated for 60 minutes and heated at 40 °C for 60 minutes, observing the exclusive formation of [{CH_2SiMe_2NDipp}$_2$AlK]$_2$ (**4^{K}**). The solution of **4^{K}** was then filtered into a fresh J. Young's NMR tube containing Rb metal and sonicated for 40 minutes to afford **4^{Rb}**. This solution was then filtered into another J. Young's NMR tube containing Cs metal and sonicated for 3 minutes, affording [{CH_2SiMe_2NDipp}$_2$AlCs]$_2$ (**4^{Cs}**). The solution of **4^{Cs}** was filtered into a new J. Young's NMR tube containing an excess of Rb metal and was sonicated for 20 minutes, however, no reaction was observed.

NMR Spectra

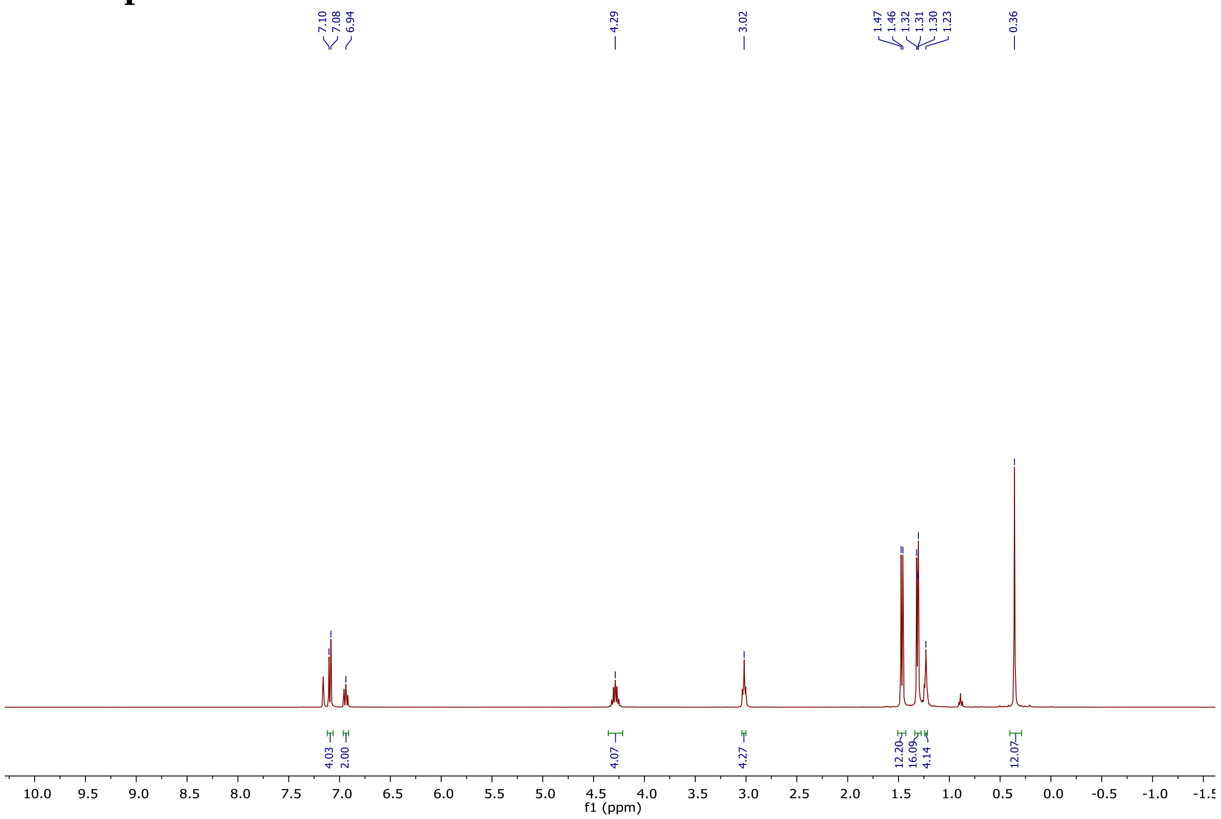

Figure S1. *^{1}H NMR Spectrum (C_6D_6, 298 K, 400.15 MHz) for* ***[{CH_2SiMe_2NDipp}$_2AlLi(THF)_2$] (4^{Li}·2THF)****.*

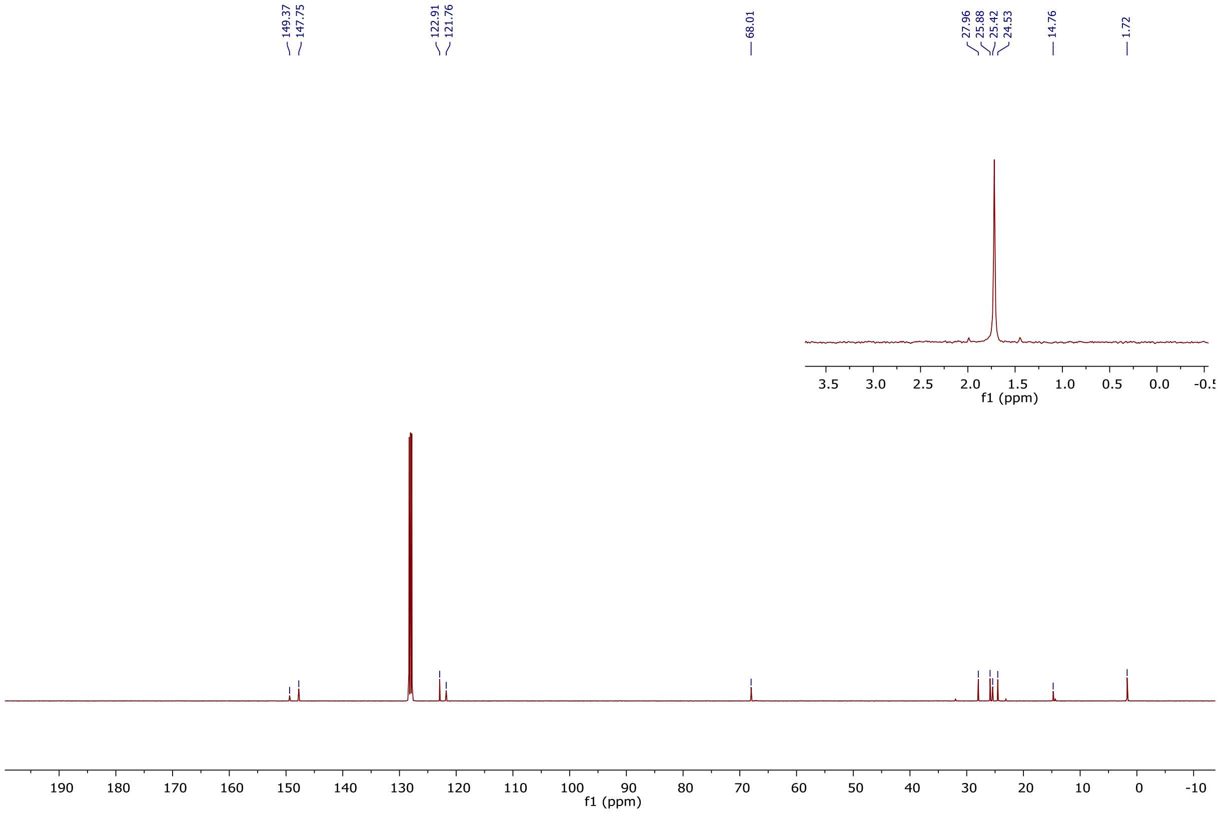

Figure S2. *$^{13}C\{^{1}H\}$ NMR Spectrum (C_6D_6, 298 K, 100.62 MHz) for* ***[{CH_2SiMe_2NDipp}$_2AlLi(THF)_2$] (4^{Li}·2THF)****.*

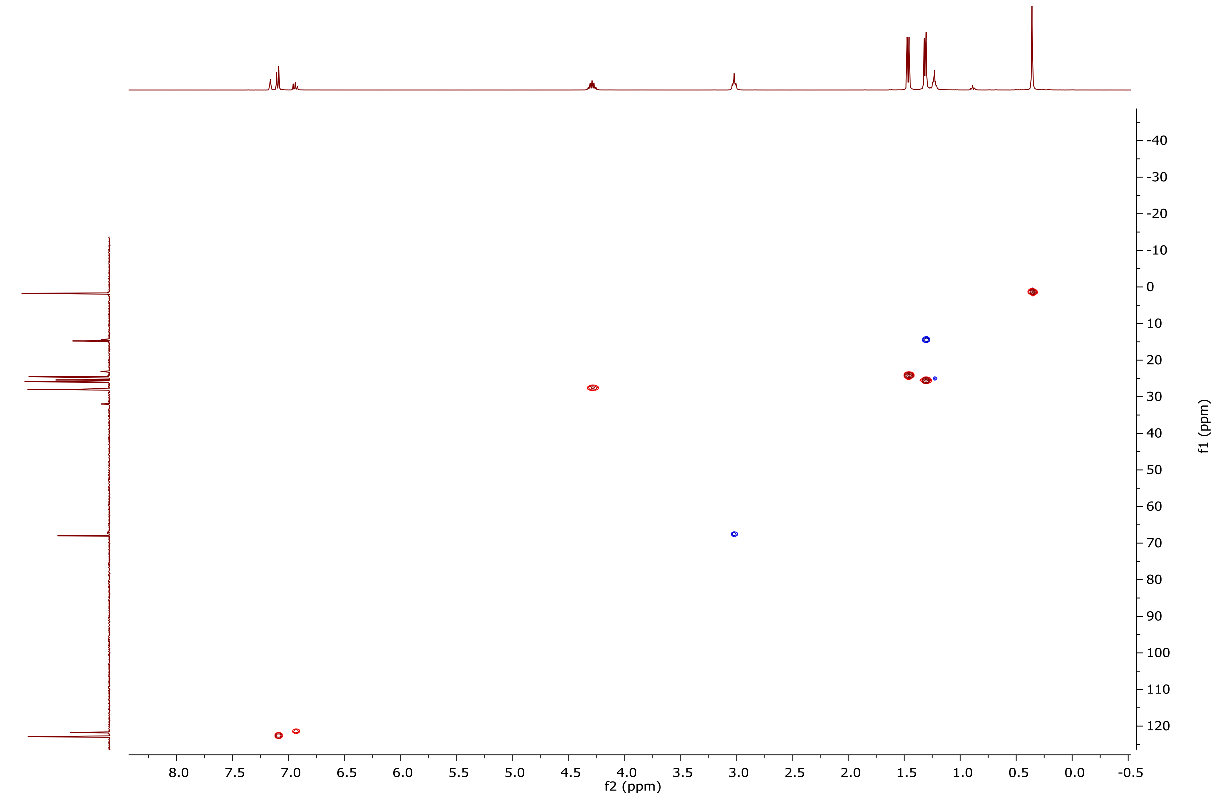

Figure S3. ^{1}H-^{13}C HSQC trace (C_6D_6, 298 K, 400.13, 100.62 MHz) for ***[{CH$_2$SiMe$_2$NDipp}$_2$AlLi(THF)$_2$] (4^{Li}·2THF)***.

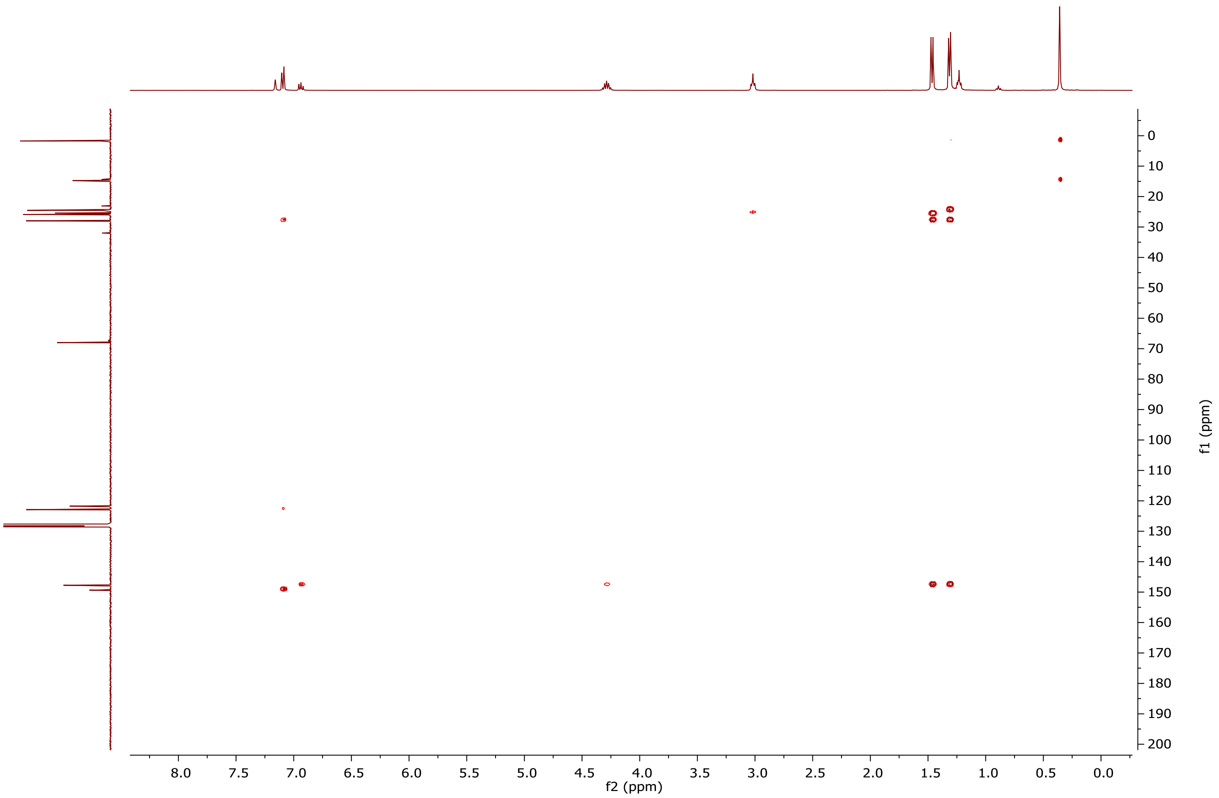

Figure S4. ^{1}H-^{13}C HMBC trace (C_6D_6, 298 K, 400.13, 100.62 MHz) for ***[{CH$_2$SiMe$_2$NDipp}$_2$AlLi(THF)$_2$] (4^{Li}·2THF)***.

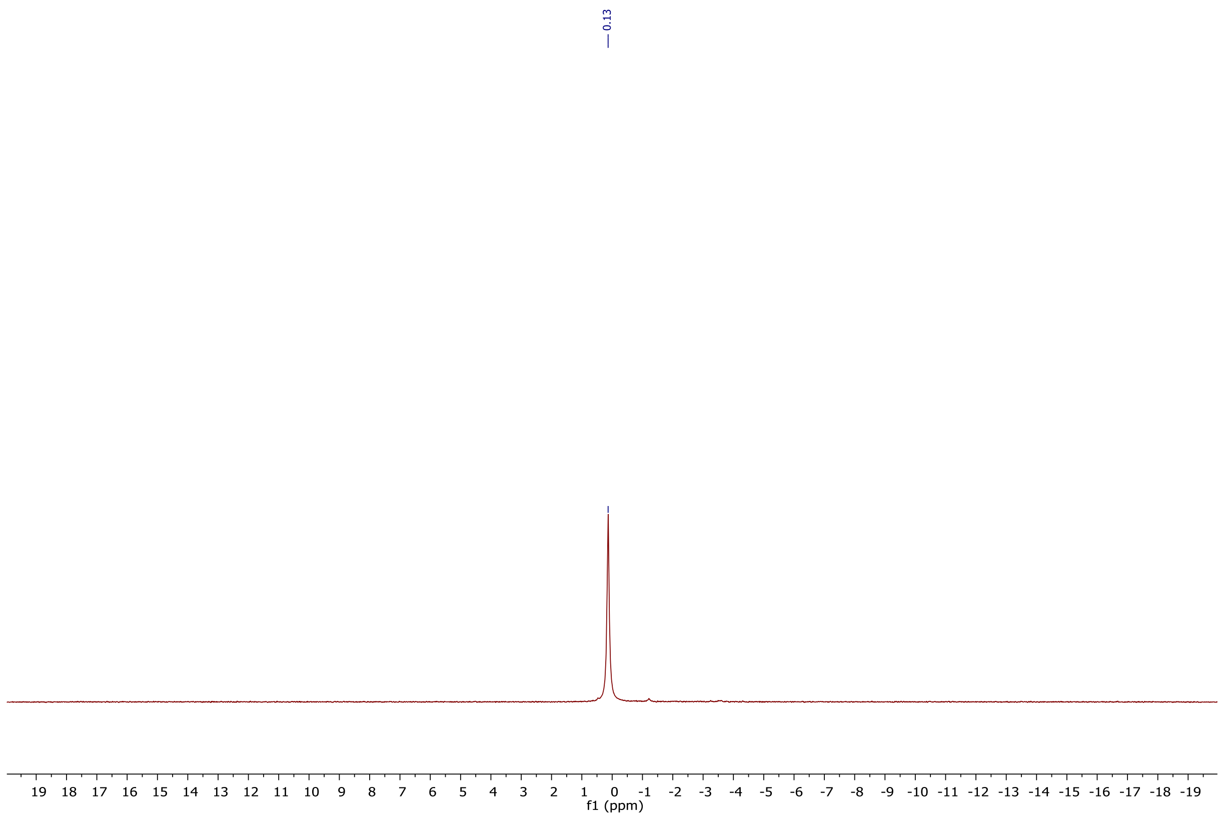

Figure S5. *^{7}Li NMR Spectrum (C_6D_6, 298 K, 194.3 MHz) for* ***$[\{CH_2SiMe_2NDipp\}_2AlLi(THF)_2]$ ($4^{Li}\cdot 2THF$)***.

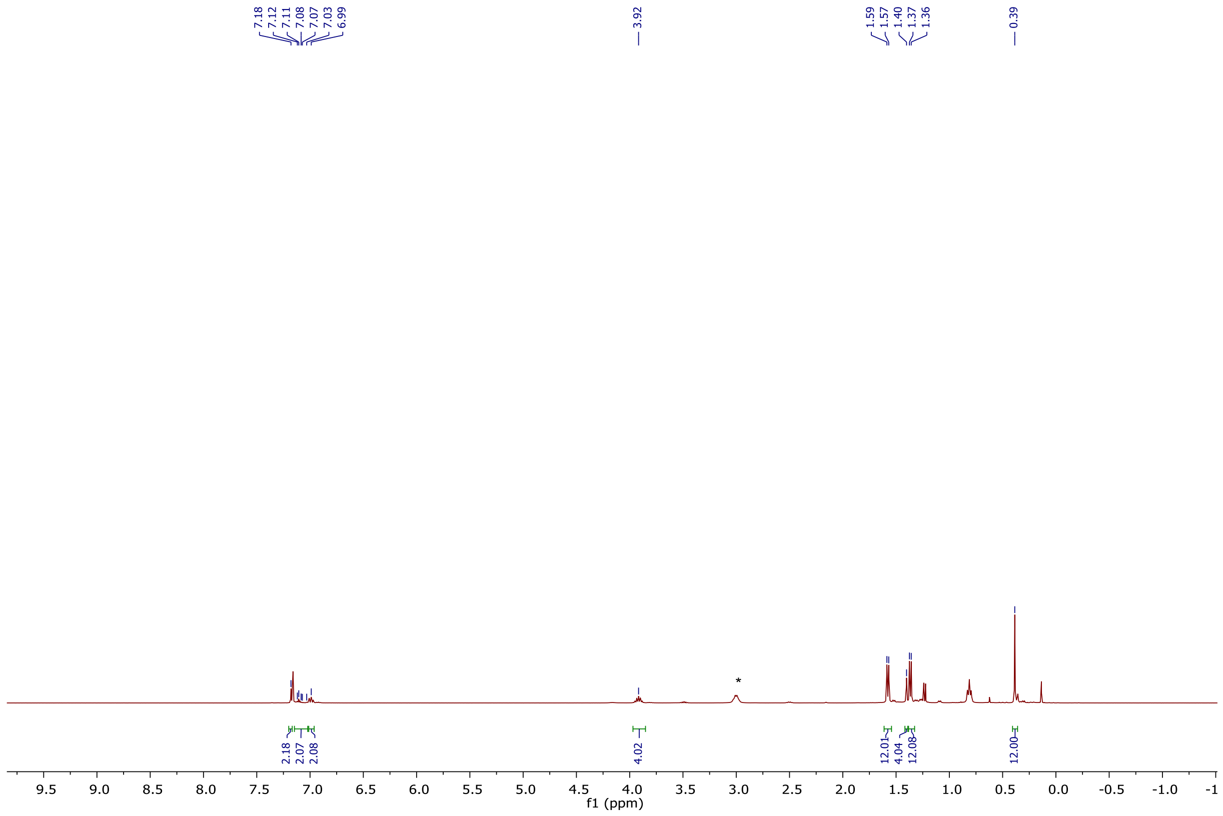

Figure S6. *^{1}H NMR Spectrum (C_6D_6, 298 K, 400.15 MHz) for* ***$[\{CH_2SiMe_2NDipp\}_2YCl_2Li]_2$****, * = residual ether.*

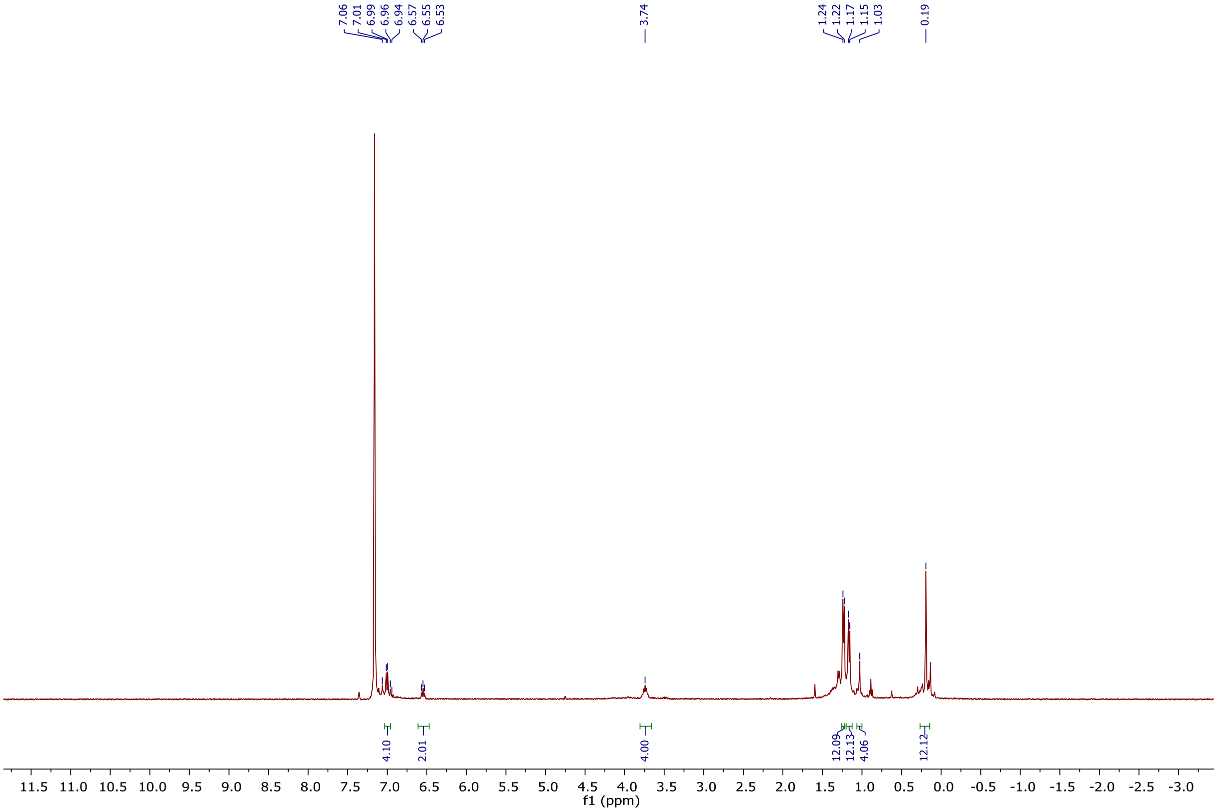

Figure S7. ^{1}H NMR Spectrum (C_6D_6, 298 K, 400.15 MHz) for **[{CH_2SiMe_2NDipp}$_2$AlLi]$_2$ (4^{Li})**.

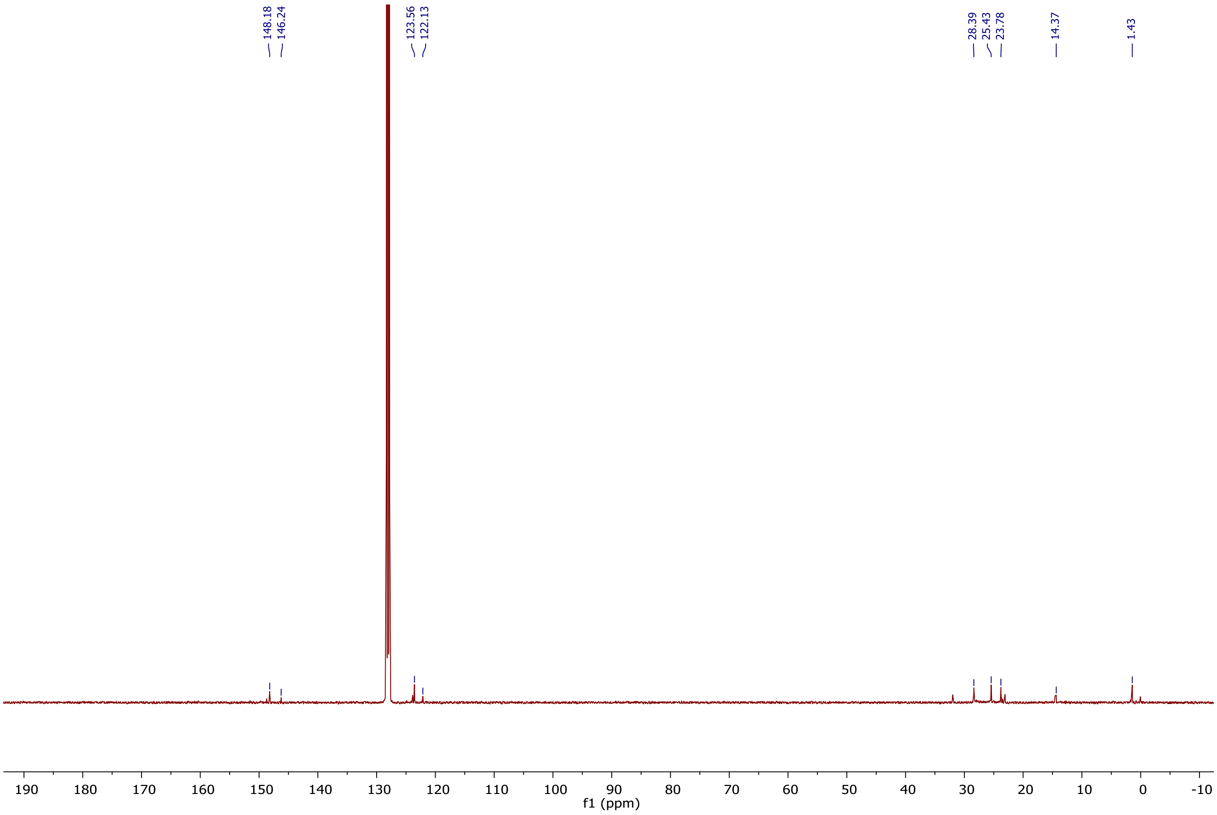

Figure S8. $^{13}C\{^{1}H\}$ NMR Spectrum (C_6D_6, 298 K, 100.62 MHz) for **[{CH_2SiMe_2NDipp}$_2$AlLi]$_2$ (4^{Li})**.

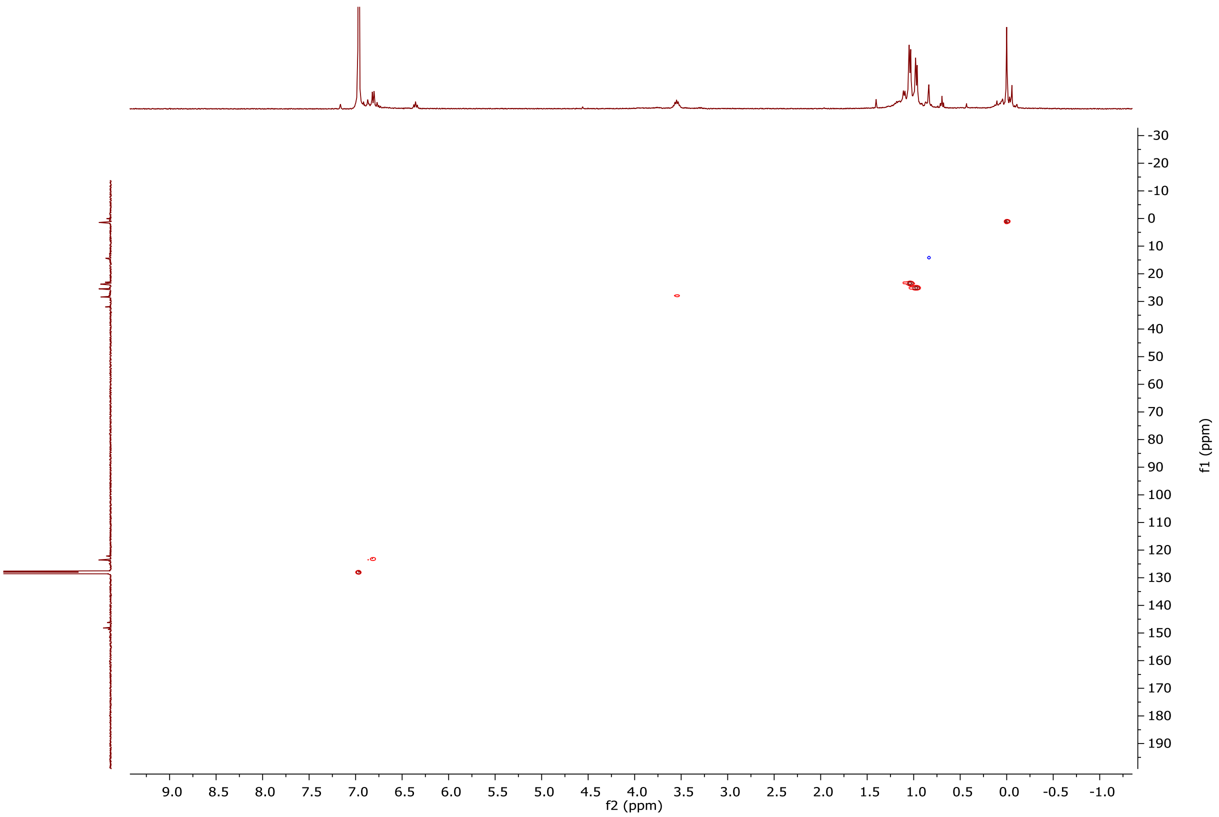

Figure S9. *¹H-¹³C HSQC trace (C_6D_6, 298 K, 400.13, 100.62 MHz) for* ***[{CH$_2$SiMe$_2$NDipp}$_2$AlLi]$_2$ (4^{Li})****.*

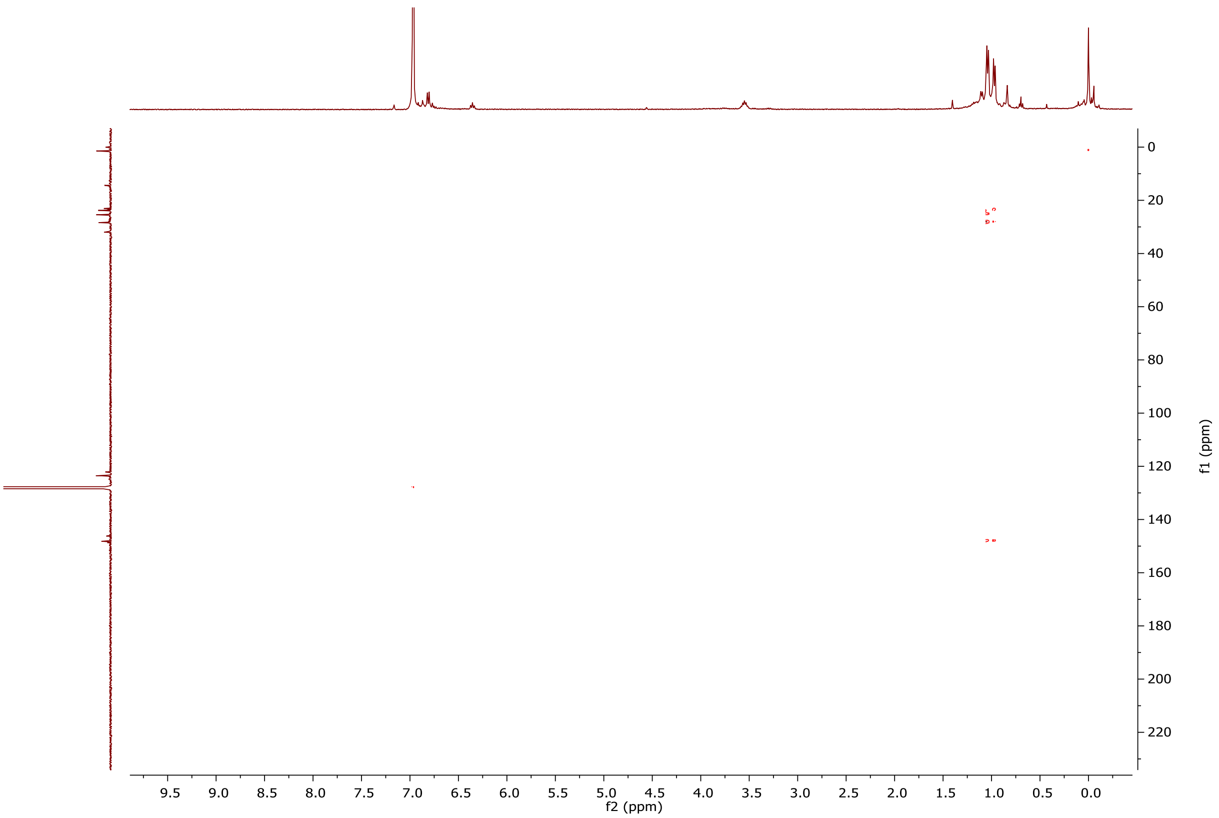

Figure S10*. ¹H-¹³C HMBC trace (C_6D_6, 298 K, 400.13, 100.62 MHz) for* ***[{CH$_2$SiMe$_2$NDipp}$_2$AlLi]$_2$ (4^{Li})****.*

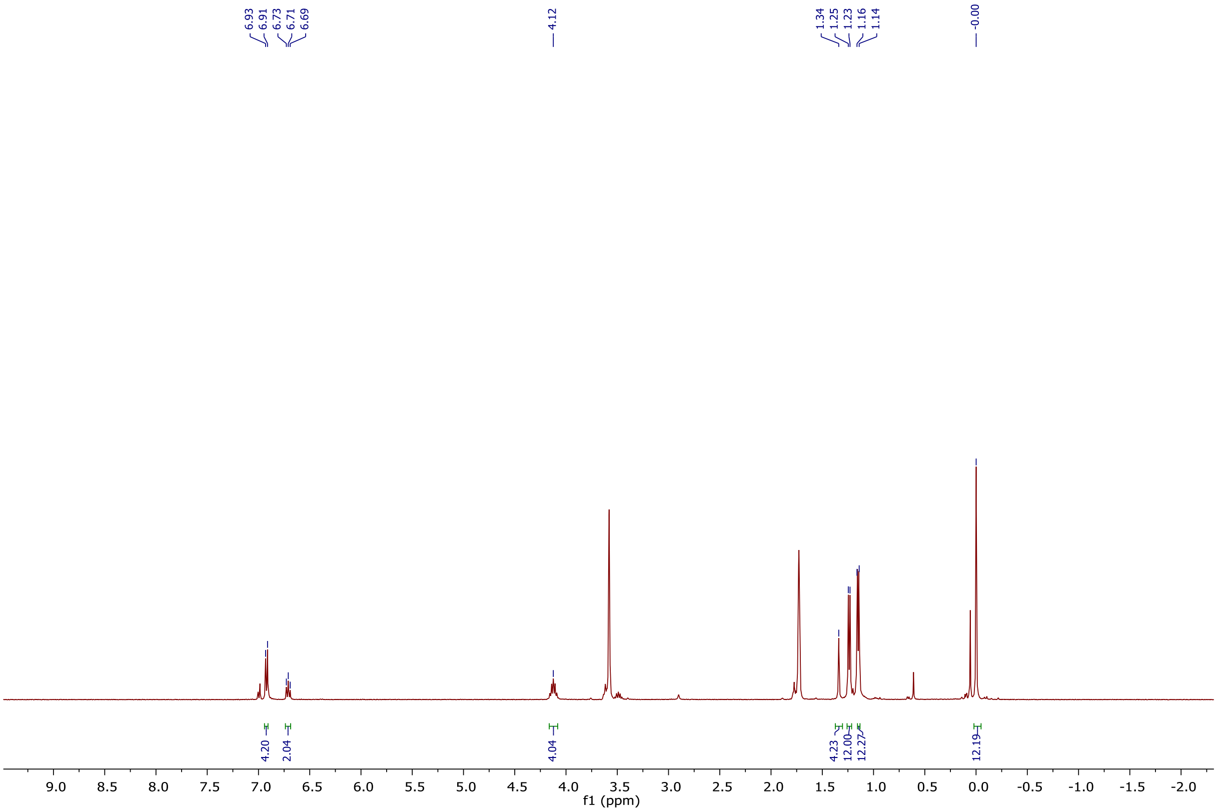

Figure S11. *^{1}H NMR Spectrum (C_4D_8O, 298 K, 400.15 MHz) for* ***$[\{CH_2SiMe_2NDipp\}_2YCl_2Rb]_n$*.**

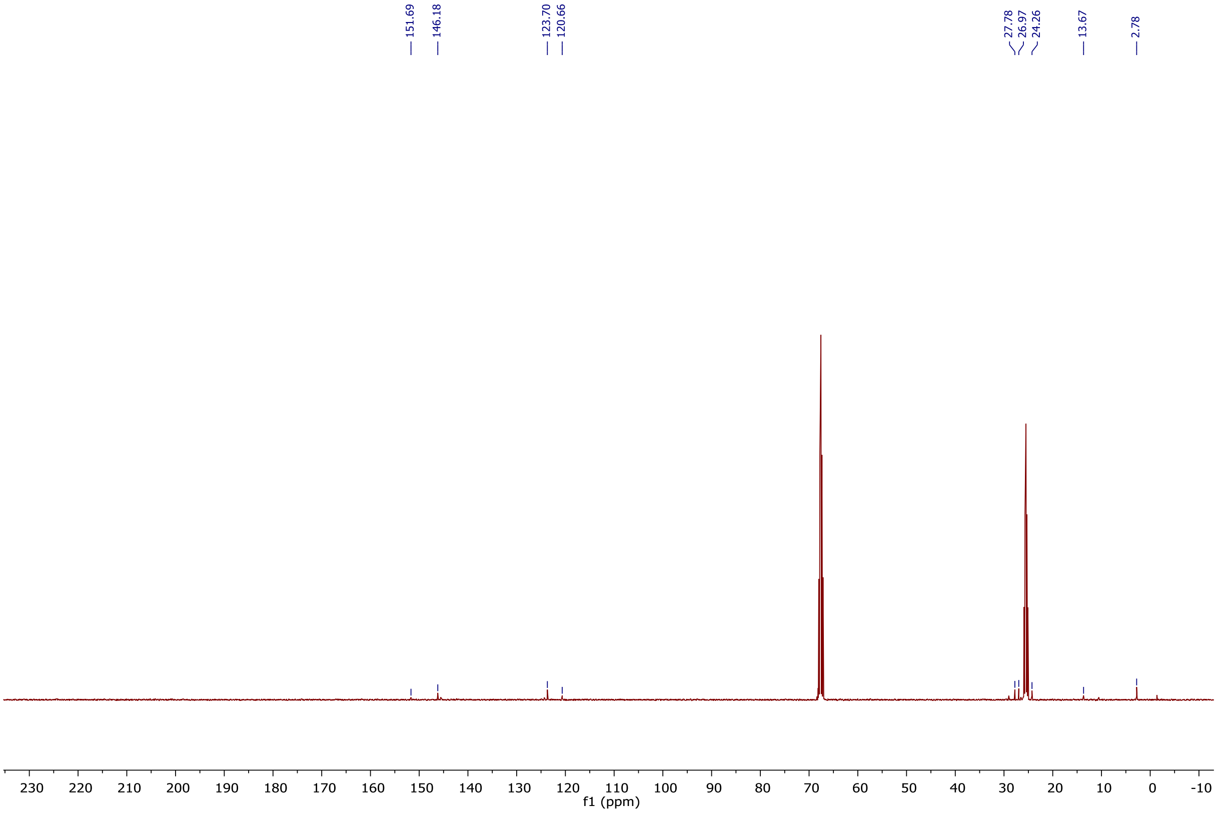

Figure S12. *$^{13}C\{^1H\}$ NMR Spectrum (C_4D_8O, 298 K, 100.62 MHz) for* ***$[\{CH_2SiMe_2NDipp\}_2YCl_2Rb]_n$*.**

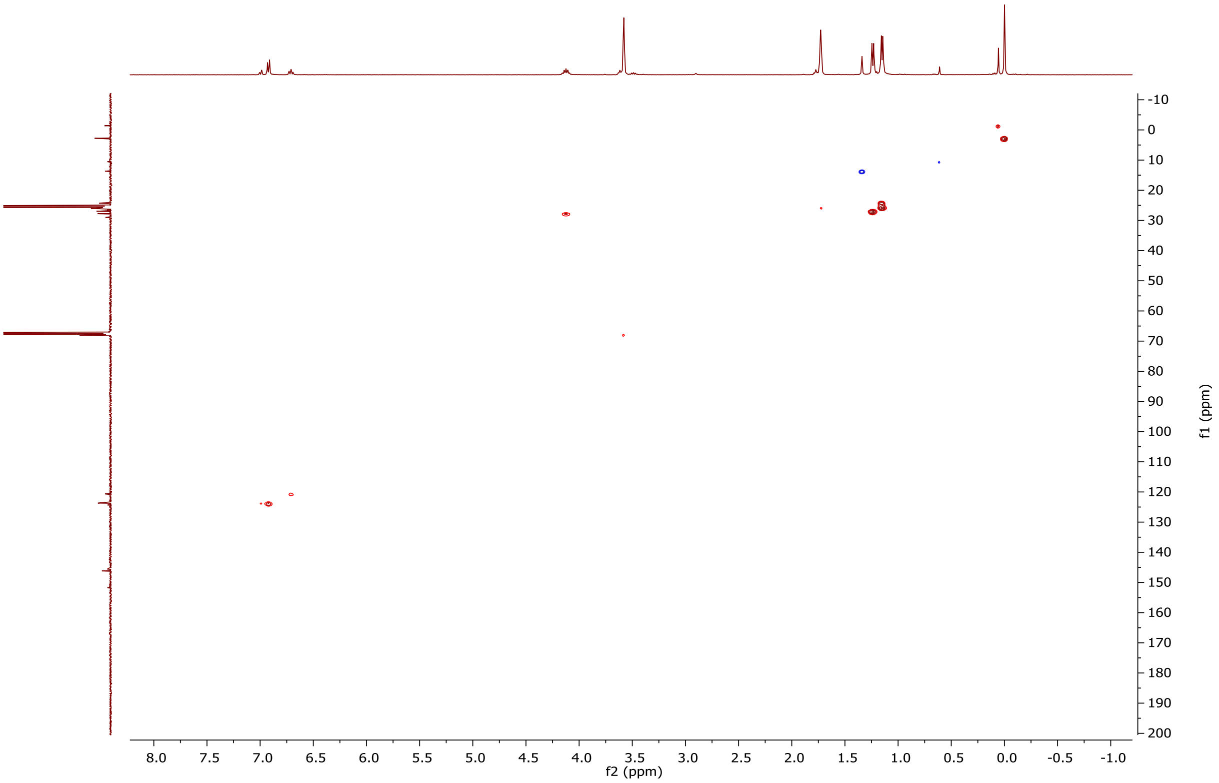

Figure S13. *^{1}H-^{13}C HSQC trace (C_4D_8O, 298 K, 400.13, 100.62 MHz) for* ***[{CH_2SiMe_2NDipp}$_2YCl_2Rb$]$_n$.***

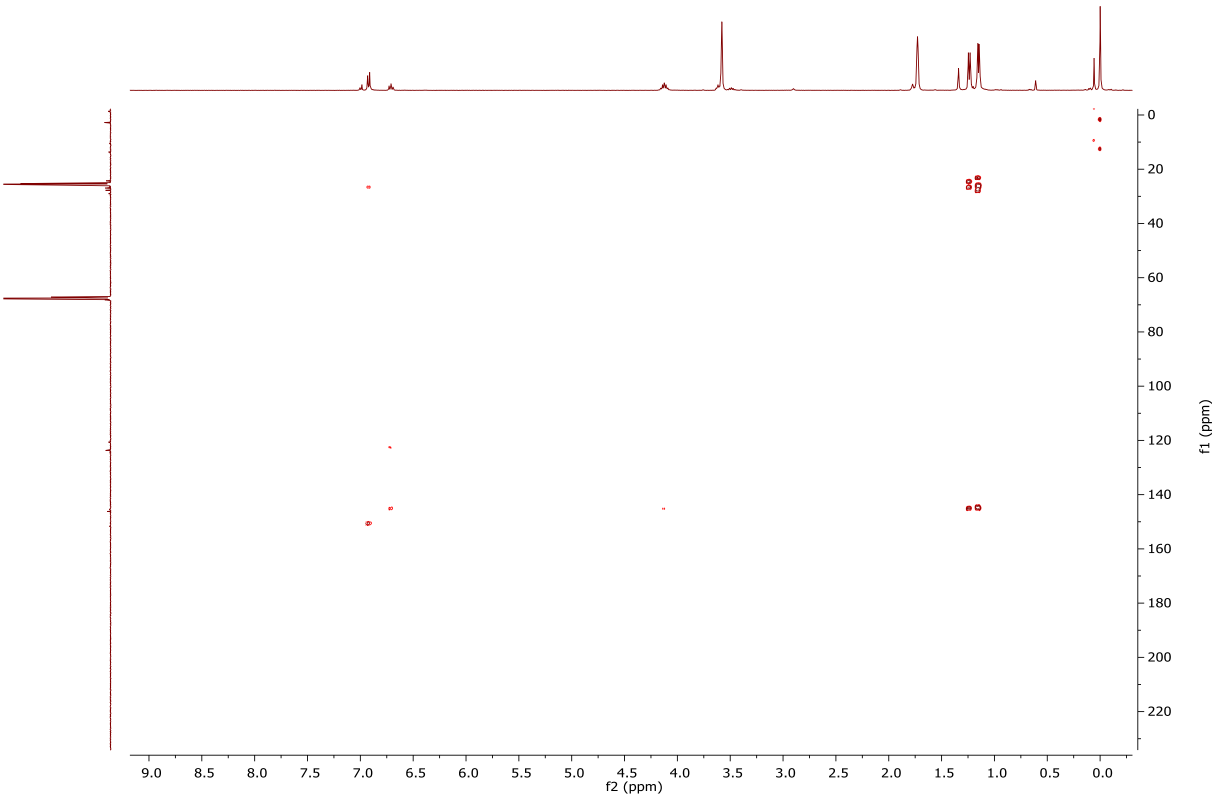

Figure S14. *^{1}H-^{13}C HMBC trace (C_4D_8O, 298 K, 400.13, 100.62 MHz) for* ***[{CH_2SiMe_2NDipp}$_2YCl_2Rb$]$_n$.***

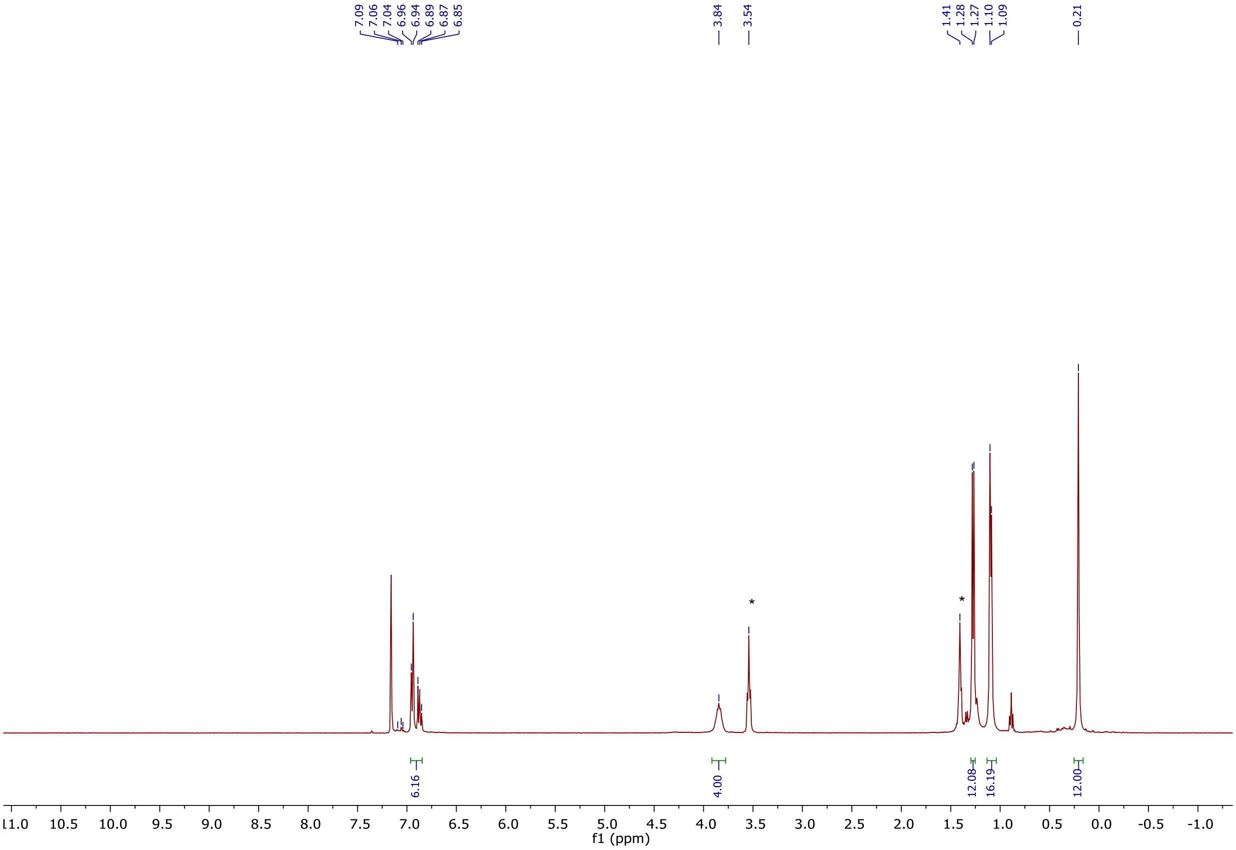

Figure S15. *^{1}H NMR Spectrum (C_6D_6, 298 K, 400.15 MHz) for* ***[{CH_2SiMe_2NDipp}$_2AlNa$]$_2$ (4^{Na})****. * = THF.*

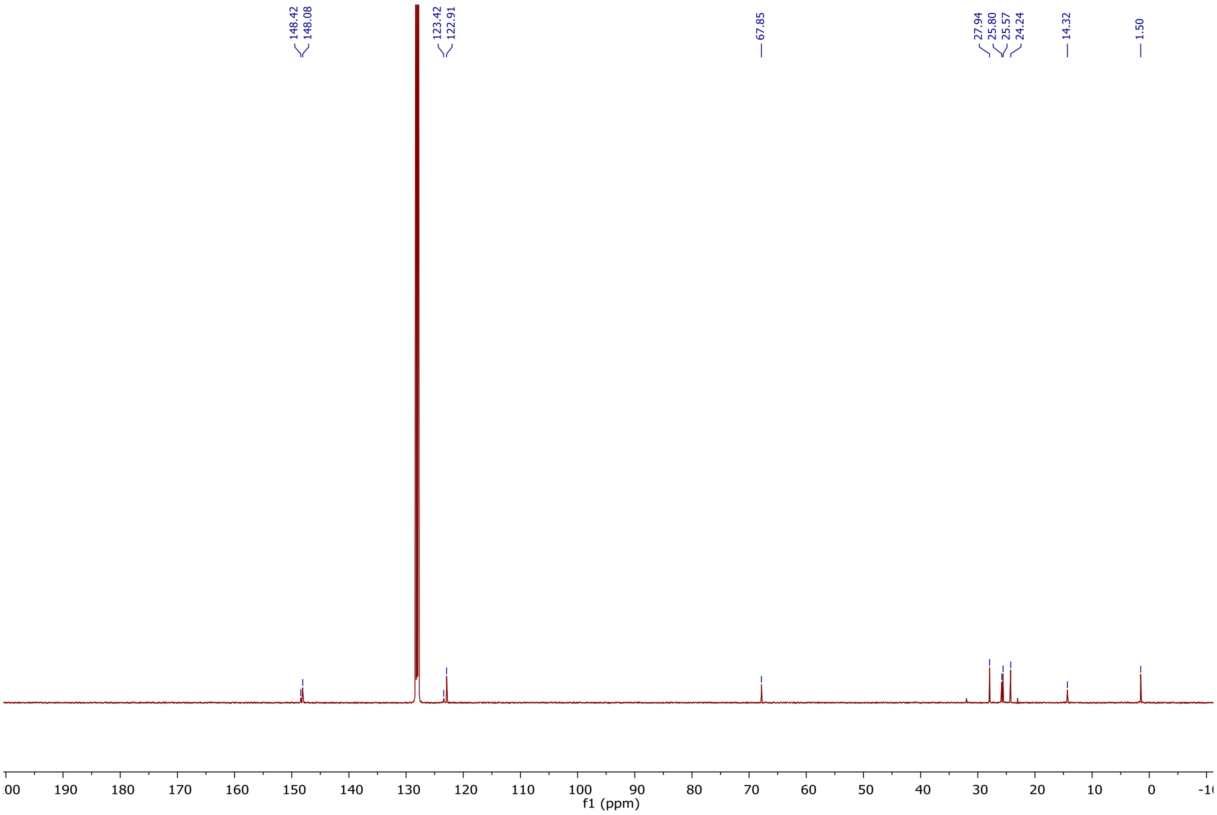

Figure S16. *^{13}C{^{1}H} NMR Spectrum (C_6D_6, 298 K, 100.62 MHz) for* ***[{CH_2SiMe_2NDipp}$_2AlNa$]$_2$ (4^{Na})****.*

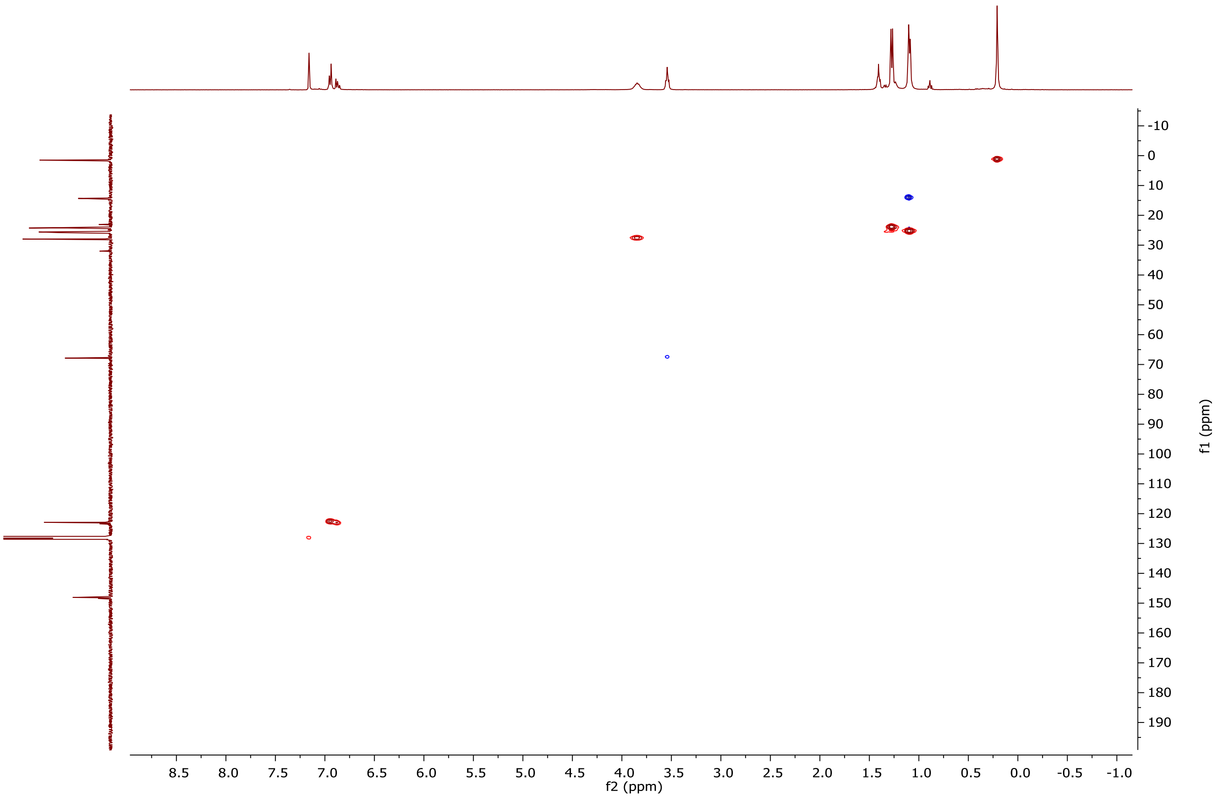

Figure S17. *^{1}H-^{13}C HSQC trace (C_6D_6, 298 K, 400.13, 100.62 MHz) for* ***$[\{CH_2SiMe_2NDipp\}_2AlNa]_2$ (4^{Na})***.

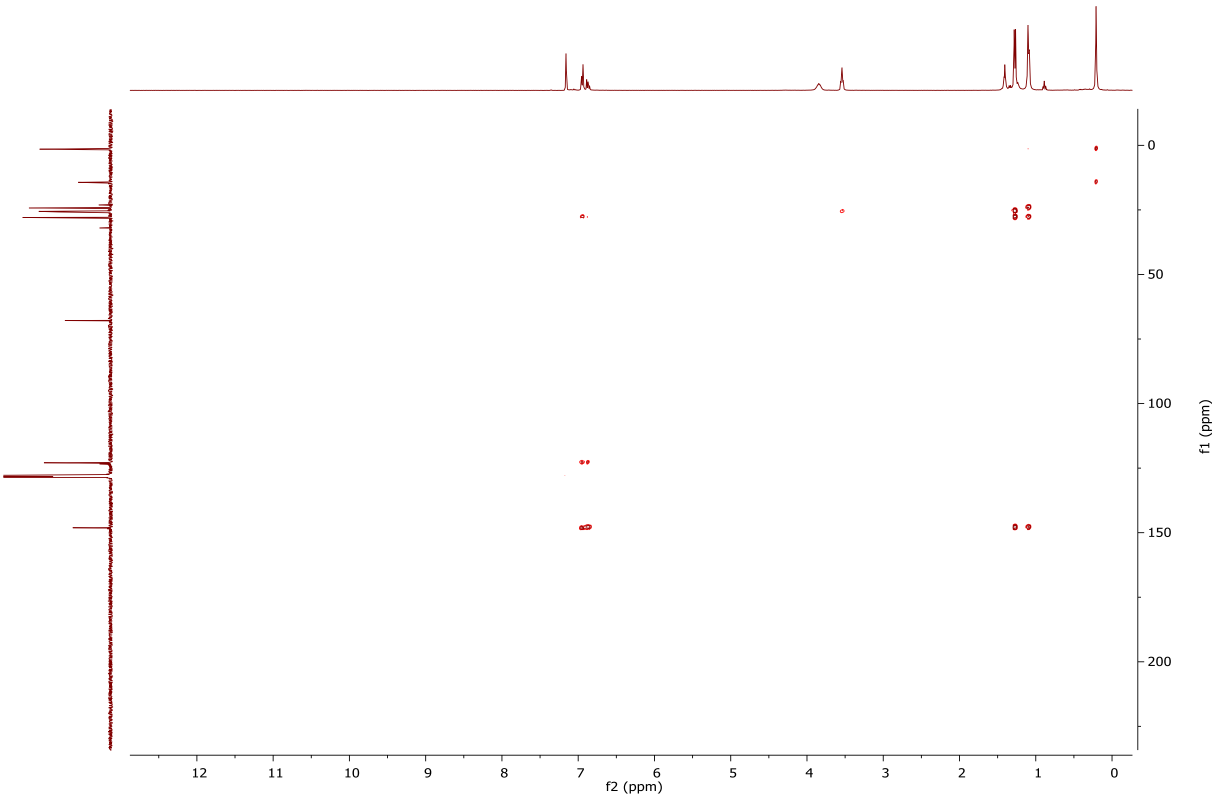

Figure S18. *^{1}H-^{13}C HMBC trace (C_6D_6, 298 K, 400.13, 100.62 MHz) for* ***$[\{CH_2SiMe_2NDipp\}_2AlNa]_2$ (4^{Na})***.

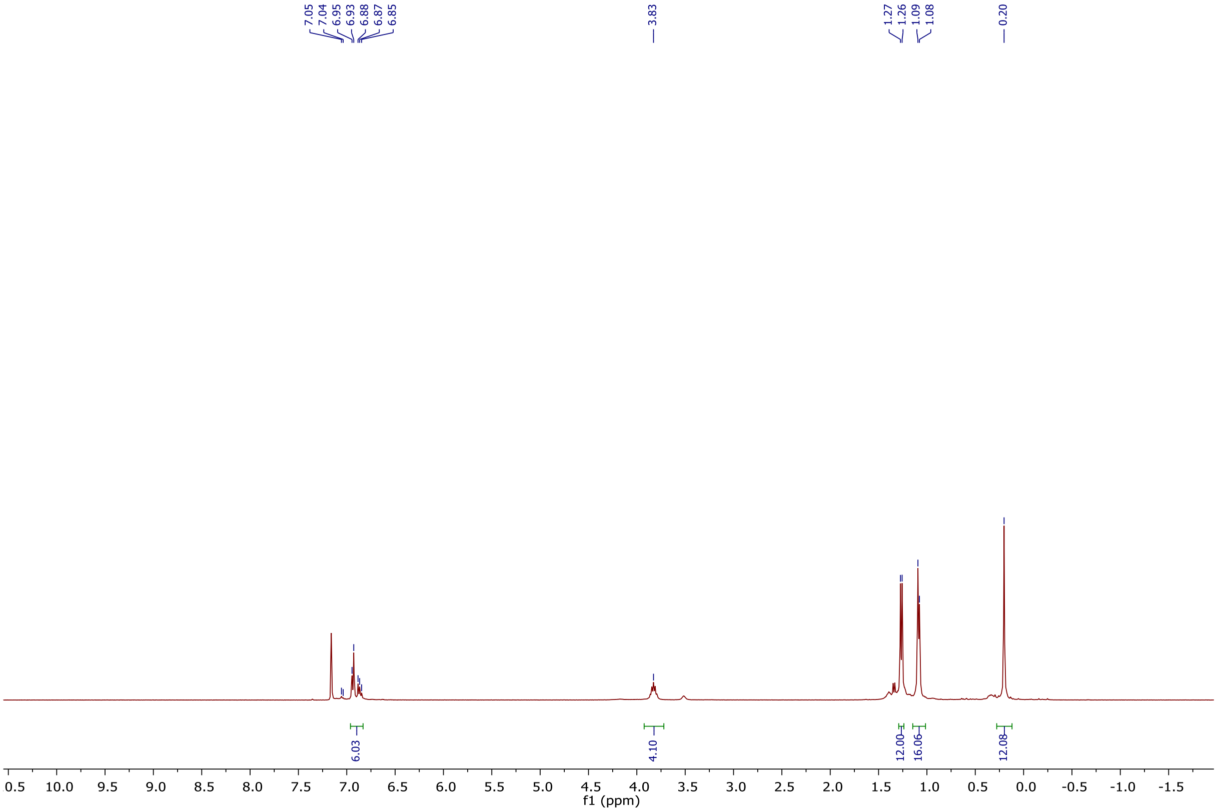

Figure S19. *^{1}H NMR Spectrum (C_6D_6, 298 K, 400.15 MHz) for* ***[{CH$_2$SiMe$_2$NDipp}$_2$AlNa]$_2$ (4^{Na})*** *after freeze-drying.*

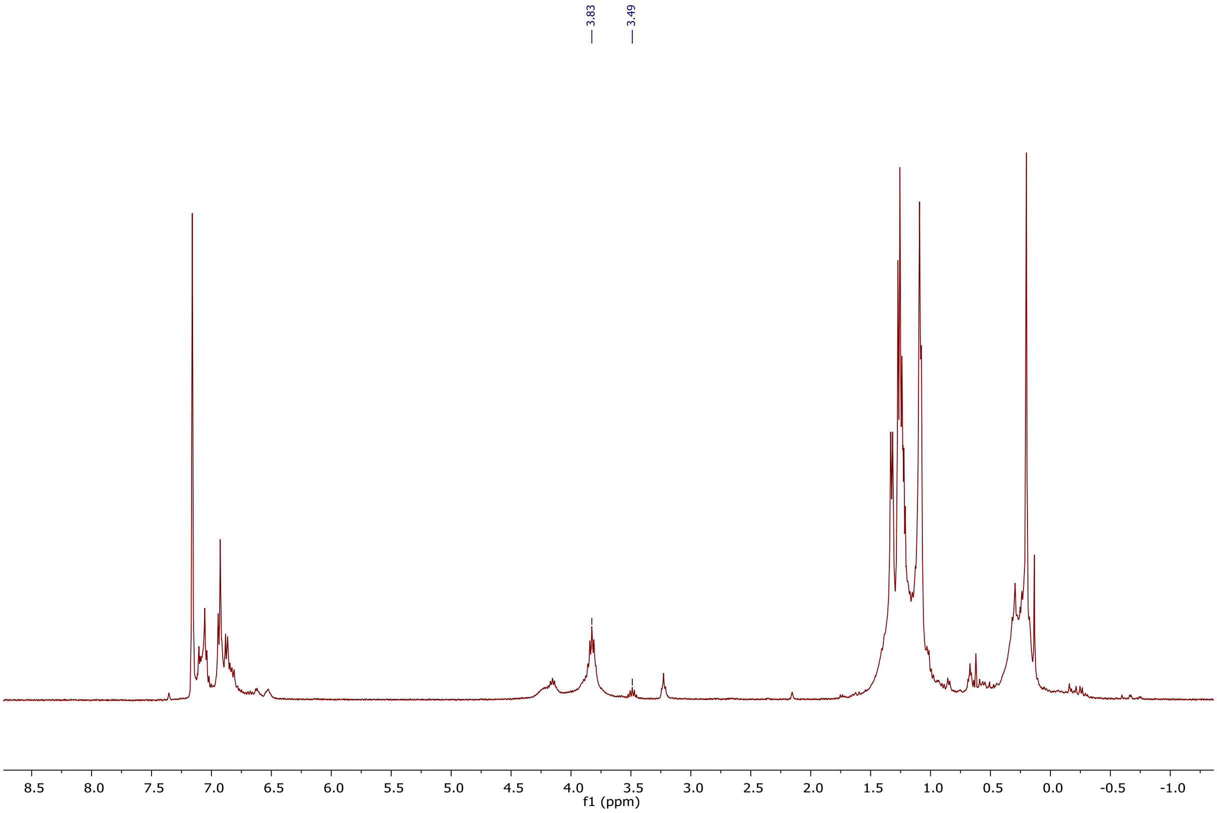

Figure S20. *^{1}H NMR Spectrum (C_6D_6, 298 K, 400.15 MHz) for* ***[{CH$_2$SiMe$_2$NDipp}$_2$AlNa]$_2$ (4^{Na})*** *sample after freeze-drying twice.*

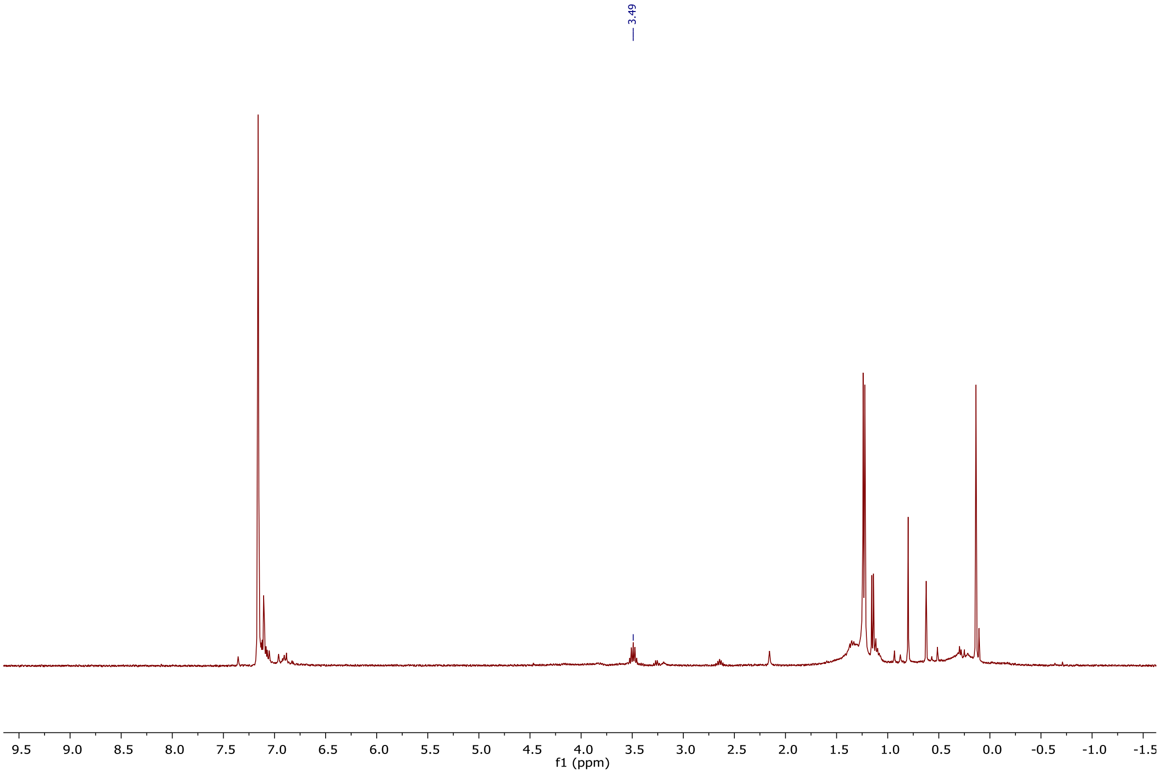

Figure S21. *^{1}H NMR Spectrum (C_6D_6, 298 K, 400.15 MHz) for* ***[{CH$_2$SiMe$_2$NDipp}$_2$AlNa]$_2$ (4^{Na})*** *sample after prolonged exposure to vacuum.*

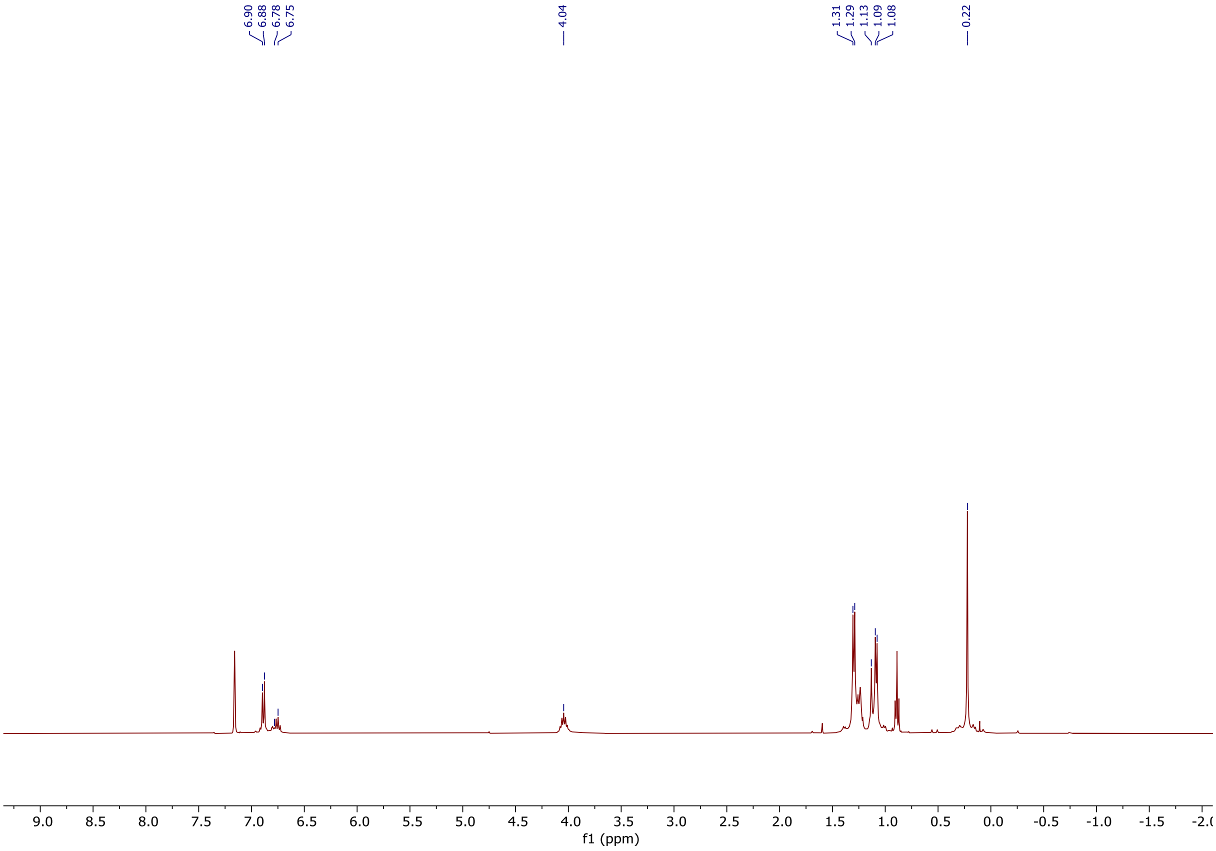

Figure S22. *^{1}H NMR Spectrum (C_6D_6, 298 K, 400.15 MHz)for* ***[{CH$_2$SiMe$_2$NDipp}$_2$AlRb]$_2$ (4^{Rb})****.*

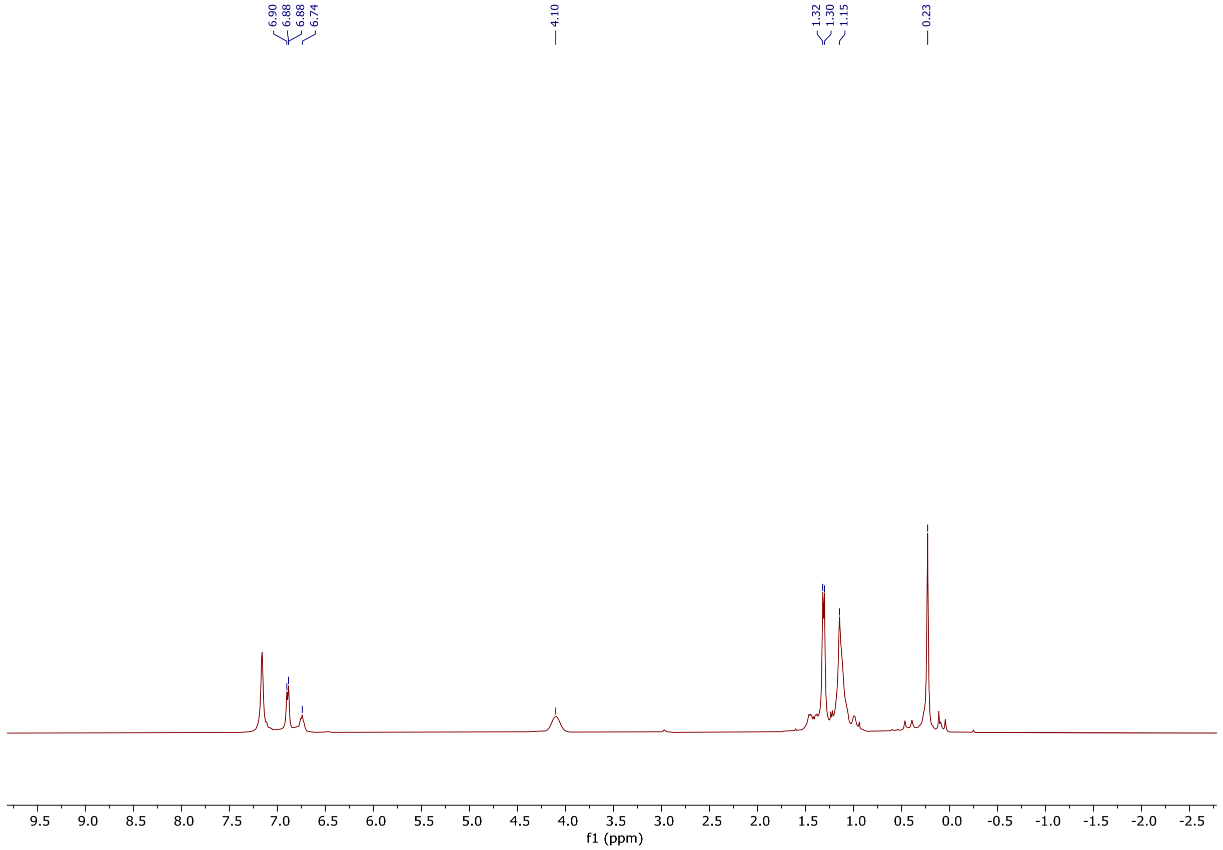

Figure S23. *^{1}H NMR Spectrum (C_6D_6, 298 K, 400.15 MHz) for* ***[{CH$_2$SiMe$_2$NDipp}$_2$AlCs]$_2$ (4^{Cs})***.

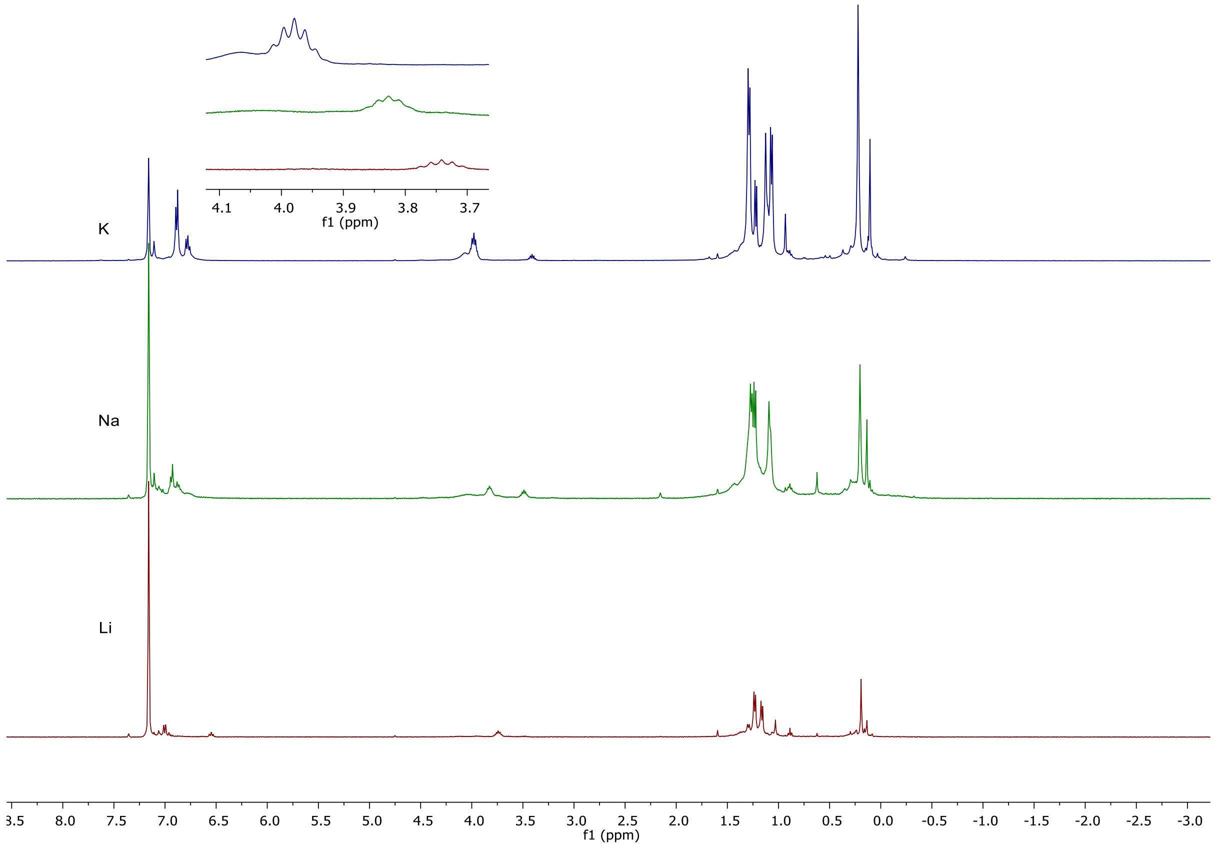

Figure S24. *Overlayed ^{1}H NMR Spectra (C_6D_6, 298 K, 400.15 MHz) for the sequential reduction of (bottom to top)* ***[{CH$_2$SiMe$_2$NDipp}$_2$AlLi]$_2$ (4^{Li}) and the formation of 4^{Na} and 4^{K}.***

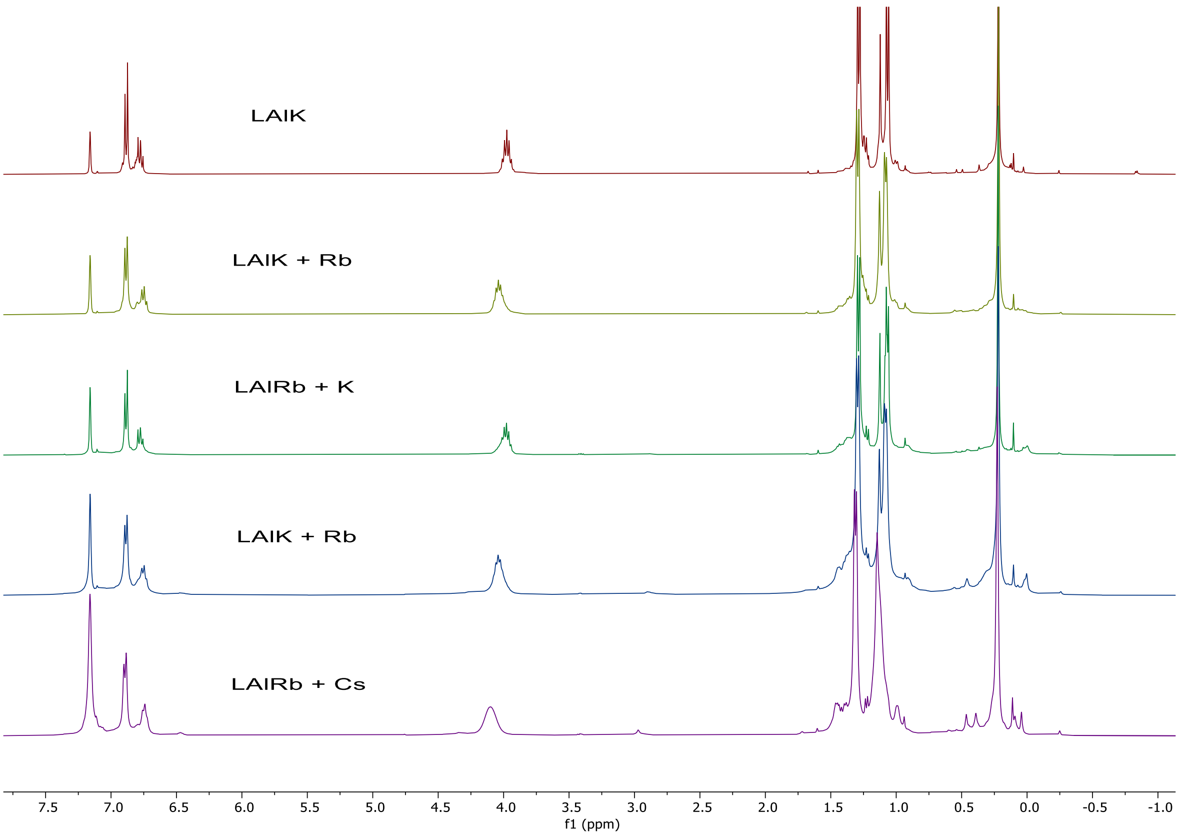

Figure S25. *Overlayed ^{1}H NMR Spectra (C_6D_6, 298 K, 400.15 MHz) for the sequential reduction of (top to bottom)* ***[{CH₂SiMe₂NDipp}₂AlK]₂ (4^K), its interconversion with (4^Rb) and the formation of (4^Cs).***

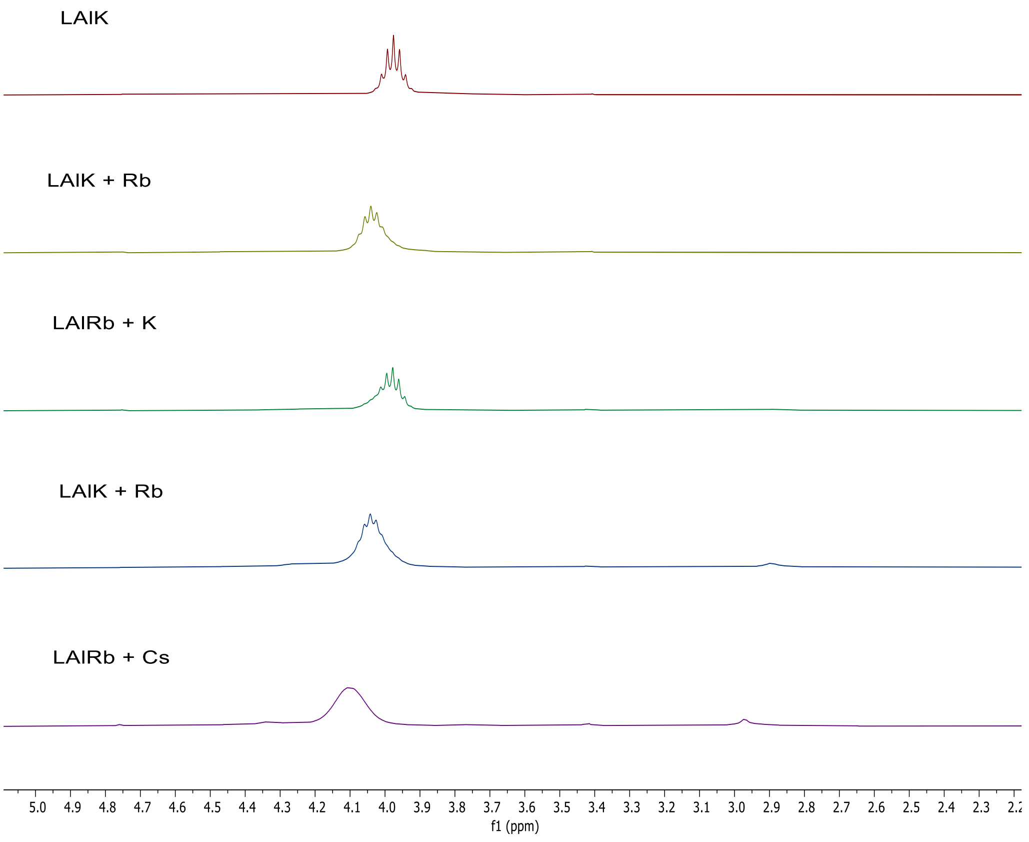

Figure S26. *Expanded Dipp-methine region from the overlayed ^{1}H NMR Spectra (C_6D_6, 298 K, 400.15 MHz) of the sequential reduction of (top to bottom)* ***[{CH₂SiMe₂NDipp}₂AlK]₂, its interconversion with (4^Rb) and the formation of (4^Cs).***

Crystallographic Details

Single Crystal X-ray diffraction data for compounds [{CH_2SiMe_2NDipp}$_2$AlLi(THF)$_2$] (**4^{Li}·2THF**) and [{CH_2SiMe_2NDipp}$_2$AlLi]$_2$ (**4^{Li}**) were collected on an Agilent SuperNova EosS2 diffractometer using Cu-Kα (1.54184 Å) radiation. Data for [{CH_2SiMe_2NDipp}$_2$YCl$_2$Li]$_2$ were collected on an Agilent Xcalibur diffractomer using Mo-Kα radiation (λ = 0.71073 Å), while those for [{CH_2SiMe_2NDipp}$_2$YCl$_2$Rb]$_n$ and [{CH_2SiMe_2NDipp}$_2$AlNa]$_2$ (**4^{Na}**) were collected on an XtaLAB Synergy, Dualflex, HyPix-Arc 100 diffractometer using Cu-Kα (1.54184 Å) radiation. In each case, the crystals were maintained at 150 K during data collection. Using Olex2,[4] the structures were solved with the olex2.solve[5] structure solution program or ShelXT and refined with the ShelXL[6] refinement package using Least−Squares minimisation.

The asymmetric unit in **4^{Li}·2THF** is comprised of one thf-coordinated lithium alumanyl molecule. Two-fold disorder, located on two of the Dipp-isopropyl groups (C13-C18), was readily modelled in a 40:60 ratio. ADP restraints were applied to fractional occupancy atoms to assist convergence. Refined as a 2-component inversion twin.

The asymmetric unit for (**4^{Li}**) is comprised of half of a dimeric unit, plus a molecule of benzene. All moieties are completed by virtue of space-group inversion symmetry. ADP restraints were employed, on merit to assist convergence.

Half of a dimer constitutes the asymmetric unit in [{CH_2SiMe_2NDipp}$_2$YCl$_2$Li]$_2$. The remainder of the molecule arises by virtue of crystallographic inversion symmetry.

The asymmetric unit for [{CH_2SiMe_2NDipp}$_2$YCl$_2$Rb]$_n$ is comprised of a rubidium dichloroyttrate unit which can be extended by virtue of space-group inversion symmetry to view the polymeric structure. All non-hydrogen atoms were refined anisotropically. Hydrogen atom positions were calculated geometrically and refined using the riding model.

The asymmetric unit in (**4^{Na}**) is comprised of one dimeric molecule. Two-fold disorder was located on two of the Dipp-isopropyl groups (C56-C58; C44-46), but readily modelled in a 35:65 and 80:20 ratio, respectively. ADP restraints were applied to fractional occupancy atoms to assist convergence. Refined as a 2-component inversion twin

Crystallographic data for all compounds have been deposited with the Cambridge Crystallographic Data Centre as supplementary publications CCDC 2452423-2452427 for **4^{Li}·2THF**, **4^{Li}**, [{CH_2SiMe_2NDipp}$_2$YCl$_2$Li]$_2$, [{CH_2SiMe_2NDipp}$_2$YCl$_2$Rb]$_n$ and **4^{Na}**, respectively. Copies of these data can be obtained free of charge on application to CCDC, 12 Union Road, Cambridge CB2 1EZ, UK [fax(+44) 1223 336033], e-mail: deposit@ccdc.cam.ac.uk.

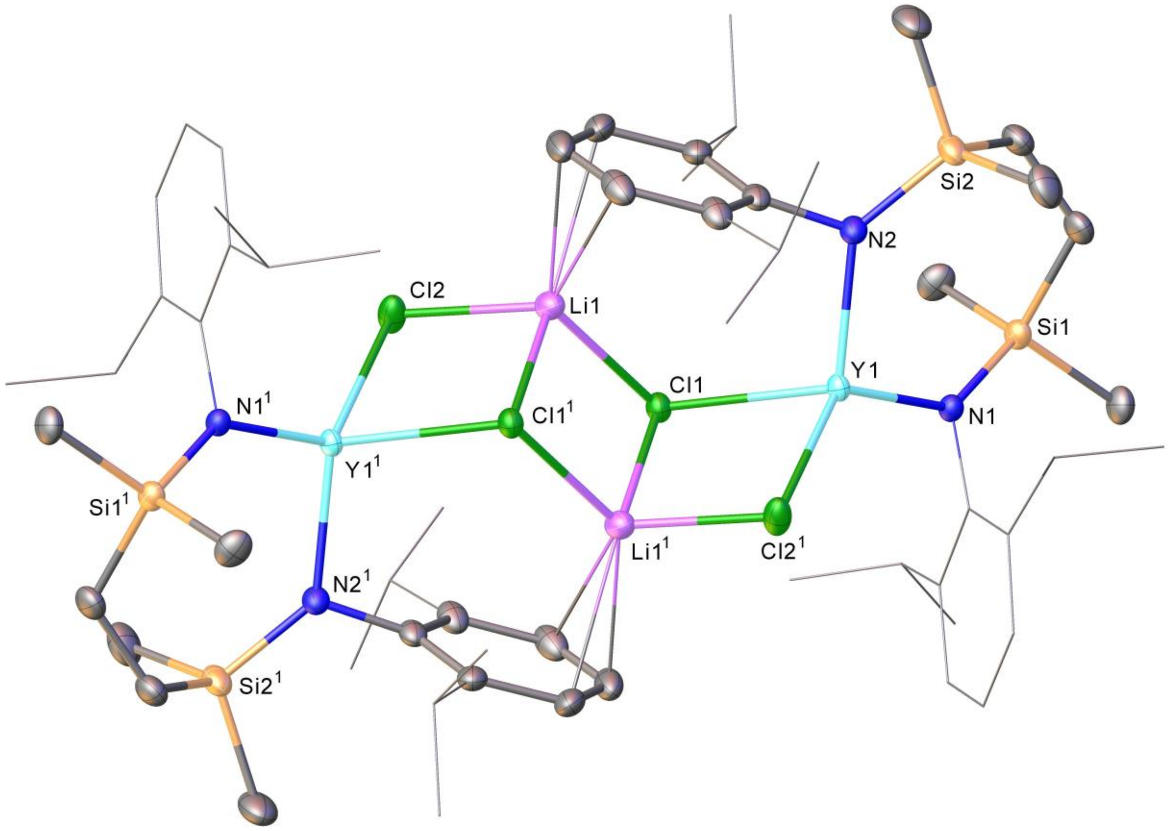

Figure S27. Molecular structure of [{CH_2SiMe_2NDipp}$_2YCl_2Li$]$_2$ (30% probability ellipsoids). For clarity, hydrogen and disordered atoms are omitted. Similarly, Dipp groups not involved in π···arene interactions are displayed as wireframe. Symmetry operations to generate primed atoms: ^{1}1-*x*, 1-*y*, 1-*z*.

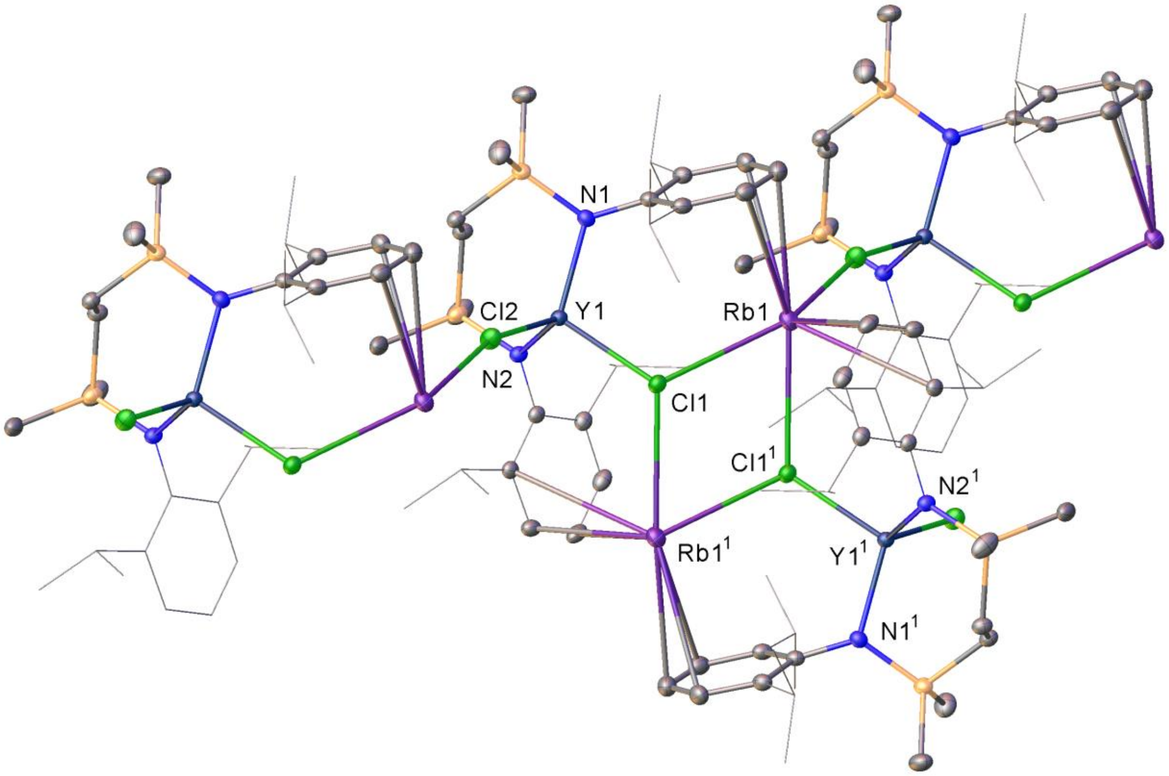

Figure S28. Molecular structure of [{CH_2SiMe_2NDipp}$_2YCl_2Rb$]$_n$ (30% probability ellipsoids). For clarity, hydrogen and disordered atoms are omitted. Similarly, Dipp groups not involved in π···arene interactions are displayed as wireframe. Symmetry operations to generate primed atoms: ^{1}1-*x*, 1-*y*, 1-*z*; ^{2}-1+*x*, +*y*, +*z*.

***Table S1**. Crystal Data and Structure Refinement for Compounds **4^{Li}·2THF, 4^{Li}** and [{CH_2SiMe_2NDipp}$_2YCl_2Li$]$_2$.*

Identification code	s25msh15 (**4^{Li}·2THF**)	s25msh03 (**4^{Li}**)	e23msh07 [{CH_2SiMe_2NDipp}$_2YCl_2Li$]$_2$
Empirical formula	$C_{38}H_{66}AlLiN_2O_2Si_2$	$C_{72}H_{112}Al_2Li_2N_4Si_4$	$C_{30}H_{50}Cl_2LiN_2Si_2Y$
Formula weight	673.02	1213.85	661.65
Crystal system	monoclinic	triclinic	triclinic
Space group	$P2_1$	P-1	P-1
a/ Å	9.89440(10)	10.0918(3)	12.1789(4)
b/ Å	18.11170(10)	11.8653(4)	12.1827(4)
c/ Å	12.41610(10)	16.1802(6)	12.3361(5)
α/ °	90	72.810(3)	88.756(3)
β/ °	110.3830(10)	84.514(3)	78.031(3)
γ/ °	90	81.842(3)	77.850(3)
U/ Å^3	2085.70(3)	1829.23(11)	1749.97(11)
Z	2	1	2
ρ_{calc}/ g cm^{-3}	1.072	1.102	1.256
μ/ mm^{-1}	1.206	1.289	1.908
F(000)	736.0	660.0	696.0
Crystal size/ mm^3	0.51 × 0.43 × 0.33	0.23 × 0.18 × 0.08	0.373 × 0.319 × 0.269
2θ range for data collection/°	7.596 to 146.426	7.856 to 146.358	5.912 to 60.874
Index ranges	$-10 \le h \le 12$,<br>$-22 \le k \le 21$,<br>$-15 \le l \le 15$	$-12 \le h \le 12$,<br>$-8 \le k \le 14$,<br>$-19 \le l \le 20$	$-15 \le h \le 17$,<br>$-16 \le k \le 16$,<br>$-12 \le l \le 16$
Reflections collected	24354	13347	16499
Independent reflections, R_{int}	7092, 0.0229	7167, 0.0368]	8868, 0.0288
Data/restraints/parameters	7092/49/462	7167/36/391	8868/0/355
Goodness–of–fit on F^2	1.086	1.029	1.032
Final R1, wR2 [I>=2σ(I)]	0.0394, 0.1066	0.0554, 0.1367	0.0385, 0.0742
Final R1, wR2 [all data]	0.0396, 0.1070	0.0715, 0.1500	0.0550, 0.0813
Largest diff. peak/hole/ e Å^{-3}	0.38/-0.29	7.856 to 146.358	0.31/-0.33

Table S2. *Crystal Data and Structure Refinement for Compounds [{CH_2SiMe_2NDipp}$_2YCl_2Rb$]n and* $\mathbf{4}^{Na}$.

Identification code	y25msh10 [{CH_2SiMe_2NDipp}$_2YCl_2Rb$]n	y25msh32 ($\mathbf{4}^{Na}$)
Empirical formula	$C_{30}H_{50}Cl_2N_2RbSi_2Y$	$C_{60}H_{100}Al_2N_4Na_2Si_4$
Formula weight	740.18	1089.73
Crystal system	triclinic	monoclinic
Space group	*P*-1	$P2_1$
a/ Å	9.73010(10)	16.4095(4)
b/ Å	12.7122(2)	13.1233(2)
c/ Å	15.8207(3)	16.8199(4)
α/ °	69.127(2)	90
β/ °	83.4070(10)	111.404(3)
γ/ °	76.4700(10)	90
U/ $Å^3$	1776.64(5)	3372.30(14)
Z	2	2
ρ_{calc}/ g cm^{-3}	1.384	1.073
μ/ mm^{-1}	6.146	1.468
F(000)	764.0	1184.0
Crystal size/ mm^3	0.15 × 0.09 × 0.07	0.21 × 0.12 × 0.05
2□ range for data collection/°	5.982 to 159.932	5.784 to 154.088
Index ranges	$-12 \le h \le 11$, $-16 \le k \le 12$, $-20 \le l \le 19$	$-20 \le h \le 20$, $-13 \le k \le 16$, $-20 \le l \le 21$
Reflections collected	26772	38324
Independent reflections, R_{int}	7453, 0.0204	12062, 0.0527
Data/restraints/parameters	7453/0/366	12062/86/684
Goodness–of–fit on F^2	1.092	1.035
Final *R*1, *wR*2 [*I*>=2σ(*I*)]	0.0271, 0.0719	0.1103, 0.3260
Final *R*1, *wR*2 [all data]	0.0281, 0.0725	0.1214, 0.3335
Largest diff. peak/hole/ e $Å^{-3}$	0.38/-0.29	7.856 to 146.358

Computational Details

DFT calculations were performed with Gaussian 16 (C.01).[7] In this study, four different methodologies have been assessed, A-D, which are described in Table S3 for reactions **1-6** (Scheme S1) and highlight the different basis set and solvation correction combinations for each method. Initial BP86 optimizations were performed using the 'grid = ultrafine' option,[8] with all stationary points being fully characterized via analytical frequency calculations as minima with all positive eigenvalues. Dispersion corrections to the BP86 results employed Grimme's D3 parameter set with Becke-Johnson damping ($D3^{BJ}$),[9] and corrections for the effect of benzene (ε = 2.2706) solvent were introduced using the polarizable continuum model (PCM) for methods A and C, conductor-like polarizable continuum model (CPCM) for method B, whilst method D uses the SMD continuum universal solvation model.[10]

Method A: BP86-$D3^{BJ}$(PCM=C_6H_6)/6-311++G**&def2-TZVPP//BP86/6-31G**&SDDALL

Method B: BP86-$D3^{BJ}$(CPCM=C_6H_6)/ZORA-def2-TZVPP/SARC//BP86/6-31G**&SDDALL. (Methodology employed in *Nat. Commun.* 2023, **14**, 8147-8152)

Method C: BP86-$D3^{BJ}$(PCM=C_6H_6)/def2-TZVPP//BP86/def2-SVP

Method D: BP86-$D3^{BJ}$(SMD=C_6H_6)/def2-TZVPP//BP86/def2-SVP

Table S3. Different basis set and solvation single point energy correction details for the four different methodology approaches used to work out the Gibbs free energy of the reactions shown in Scheme S1.

Method	Optimisation Basis Set	Single Point Energy Basis Set	Solvation Energy Single Point
A	6-31G** and SDDALL **BS1**	6-311++G** and def2-TZVPP **BS2**	PCM=C_6H_6
B	6-31G** and SDDALL **BS1**	ZORA-def2-TZVPP/SARC **BS3**	CPCM=C_6H_6
C	def2-SVP **BS4**	def2-TZVPP **BS5**	PCM=C_6H_6
D	def2-SVP **BS4**	def2-TZVPP **BS5**	SMD=C_6H_6

Optimisation calculations for method A and B, describe the Si, K, Rb and Cs centres with the Stuttgart relativistic effective core potentials (RECPs)[11] and associated basis sets (SDDALL), and the 6-31G** basis set for all other atoms (BS1).[12] A polarization function was also added to Si (ζ_d = 0.284), K (ζ_d = 1.000), Rb (ζ_d = 0.491) and Cs (ζ_d = 0.306).[13] All energies were recomputed with a larger basis set

featuring 6-311++G** basis sets on all atoms (method A, BS2),[14] except for Rb and Cs, where def2-TZVPP was employed.[15] In method B, the all-electron ZORA-def2-TZVPP basis set was applied to all atoms except for Rb and Cs atoms, where ZORA(-SARC-)TZVPP was instead employed (BS3).[16] The Gibbs solvation energies and larger basis set energy correction (computing the electronic energy of M(0) in the gas phase) was computed with ORCA 5.0.1.,[17] where a combined solvation correction was obtained implicitly using the CPCM(C_6H_6) approach and a dispersion (D3^{BJ}) correction at the same basis set level.

For methods C and D, geometry optimisations were performed using the def2-SVP[15] basis set (BS4) for all atoms, with all energies corrected by a single-point calculation with the larger basis set, def2-TZVPP (BS5).[15] The energy solvation correction was computed with Gaussian 16 using both solvent models: PCM(C_6H_6) (method C) and SMD(C_6H_6) (method D) and included the def2-TZVPP basis set.

Breakdown of Energy Contributions

Table S4 details contributions of relative energies as the successive corrections to the initial SCF energy are included. Terms used are:

$\mathbf{\Delta E_{BS1}}$	SCF energy computed with the BP86 functional and BS1
$\mathbf{\Delta G_{BS1}}$	Free energy at 298.15 K and 1 atm and BS1
$\mathbf{\Delta G_{BSI/C_6H_6}}$	Free energy corrected for Benzene solvent (PCM) with BS1
$\mathbf{\Delta G_{BS1/D3^{BJ}/C_6H_6}}$	Free energy corrected for Benzene solvent (PCM) and dispersion (D3^{BJ}) with BS1
$\mathbf{\Delta E_{BS2}}$	SCF energy computed with the BP86 functional and BS2
$\mathbf{\Delta E_{BS3}}$	SCF energy computed with the BP86 functional and BS3
$\mathbf{\Delta G_{A}}$	Overall Free energy computed at the BP86-D3^{BJ}; PCM=Benzene/BS2//BP86/BS1 level
$\mathbf{\Delta G_{B}}$	Overall Free energy computed at the BP86-D3^{BJ}; CPCM=Benzene/BS3//BP86/BS1 level
$\mathbf{\Delta E_{BS4}}$	SCF energy computed with the BP86 functional and BS4
$\mathbf{\Delta G_{BS4}}$	Free energy at 298.15 K and 1 atm and BS4
$\mathbf{\Delta G_{BS4/C_6H_6}}$	Free energy corrected for Benzene solvent (PCM) with BS1
$\mathbf{\Delta G_{BS4/D3^{BJ}/C_6H_6}}$	Free energy corrected for Benzene solvent (PCM) and dispersion (D3^{BJ}) with BS4
$\mathbf{\Delta G_{BS4/SMD/C_6H_6}}$	Free energy corrected for Benzene solvent (SMD) with BS1
$\mathbf{\Delta G_{BS4/D3^{BJ}/SMD/C_6H_6}}$	Free energy corrected for Benzene solvent (SMD) and dispersion (D3^{BJ}) with BS4
$\mathbf{\Delta E_{BS5}}$	SCF energy computed with the BP86 functional and BS5
$\mathbf{\Delta G_{C}}$	Overall Free energy computed at the BP86-D3^{BJ};PCM=Benzene/BS5//BP86/BS4 level
$\mathbf{\Delta G_{\mathit{D}}}$	Overall Free energy computed at the BP86-D3^{BJ};SMD=Benzene/BS5//BP86/BS4 level

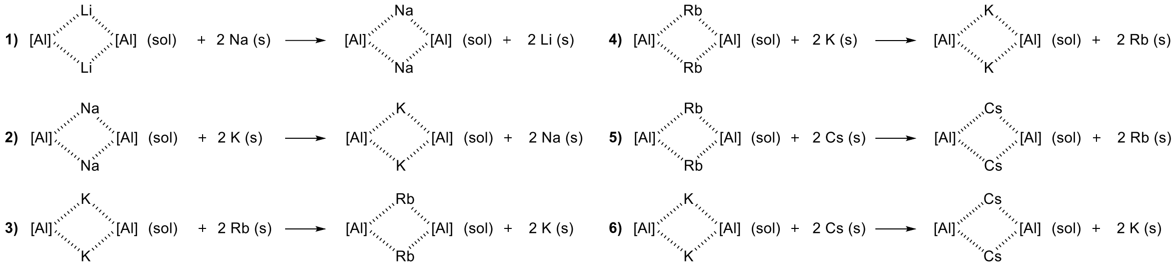

Scheme S1. Reactions of the dimeric aluminium analogues, **4^M^**, to replace the alkali metal cations,

Table S5. Relative formation energies (kcal mol^{-1}) for computed reductions of $\mathbf{4^{M}}$ (reaction: $\mathbf{4^{M}}$ → $\mathbf{4^{M'}}$), for methods A and B in solution. Data in bold are those used in the main text.

	ΔE_{BSI}	ΔG_{BSI}	$\Delta G_{BSI/C_6H_6}$	$\Delta G_{BS1/D3^{BJ}/C_6H_6}$	ΔE_{BS2}	ΔE_{BS3}	ΔG_A	ΔG_B
$\mathbf{4^{Li} \rightarrow 4^{Na}}$	23.3	18.9	12.5	5.5	21.4	13.3	3.6	8.9
$\mathbf{4^{Na} \rightarrow 4^{K}}$	−1.3	0.2	0.6	6.1	-9.8	−2.1	−2.5	−0.7
$\mathbf{4^{K} \rightarrow 4^{Rb}}$	5.0	5.1	4.5	6.3	6.1	3.2	7.4	3.4
$\mathbf{4^{Rb} \rightarrow 4^{K}}$	−5.0	−5.1	−4.5	−6.3	−6.1	−3.2	−7.4	−3.4
$\mathbf{4^{Rb} \rightarrow 4^{Cs}}$	−3.4	−4.0	−4.3	−3.6	−3.9	−0.8	−4	−1.4
$\mathbf{4^{K} \rightarrow 4^{Cs}}$	1.6	1.1	0.2	2.7	2.2	−0.5	3.3	1.9

Table S6. Relative formation energies (kcal mol^{-1}) for computed reductions of $\mathbf{4^{M}}$ (reaction: $\mathbf{4^{M}}$ → $\mathbf{4^{M'}}$), for methods C and D in solution. Data in bold are those used in the main text.

	ΔE_{BS4}	ΔG_{BS4}	$\Delta G_{BS4/C_6H_6}$	$\Delta G_{BS4/D3^{BJ}/C_6H_6}$	$\Delta G_{BS4/SMD/C_6H_6}$	$\Delta G_{BS4/D3^{BJ}/SMD/C_6H_6}$	ΔE_{BS5}	ΔG_C	ΔG_D
$\mathbf{4^{Li} \rightarrow 4^{Na}}$	18.0	17.1	9.8	3.9	14.7	8.8	20.7	6.6	11.4
$\mathbf{4^{Na} \rightarrow 4^{K}}$	−7.2	−7.0	−6.8	−2.4	−7.7	−3.3	−9.3	−4.5	−5.4
$\mathbf{4^{K} \rightarrow 4^{Rb}}$	8.1	7.6	6.4	8.5	7.7	9.7	6.6	6.9	8.2
$\mathbf{4^{Rb} \rightarrow 4^{K}}$	−8.1	−7.6	−6.4	−8.5	−7.7	−9.7	−6.6	−6.9	−8.2
$\mathbf{4^{Rb} \rightarrow 4^{Cs}}$	−1.3	−1.1	−1.2	0.4	−0.9	0.7	−3.4	−1.6	−1.3
$\mathbf{4^{K} \rightarrow 4^{Cs}}$	6.8	6.6	5.2	8.9	6.8	10.5	3.2	5.3	6.9

1. Methodology: Construction of a Combined Experimental and Computational Hess Cycle

To construct the Hess cycle of the previous reaction (**1**) (Scheme S1, Figure S29) three thermodynamic functions need to be calculated:

1. Computed Gibbs energy of the reaction in solution ($\Delta G_{rxn\ (sol.)}$)
2. Experimental Gibbs atomisation energy (ΔG_{at}) of the "metal" M(0) atoms
3. Computed Gibbs solvation energy (ΔG_{solv}) of the "metal" M(0) atoms

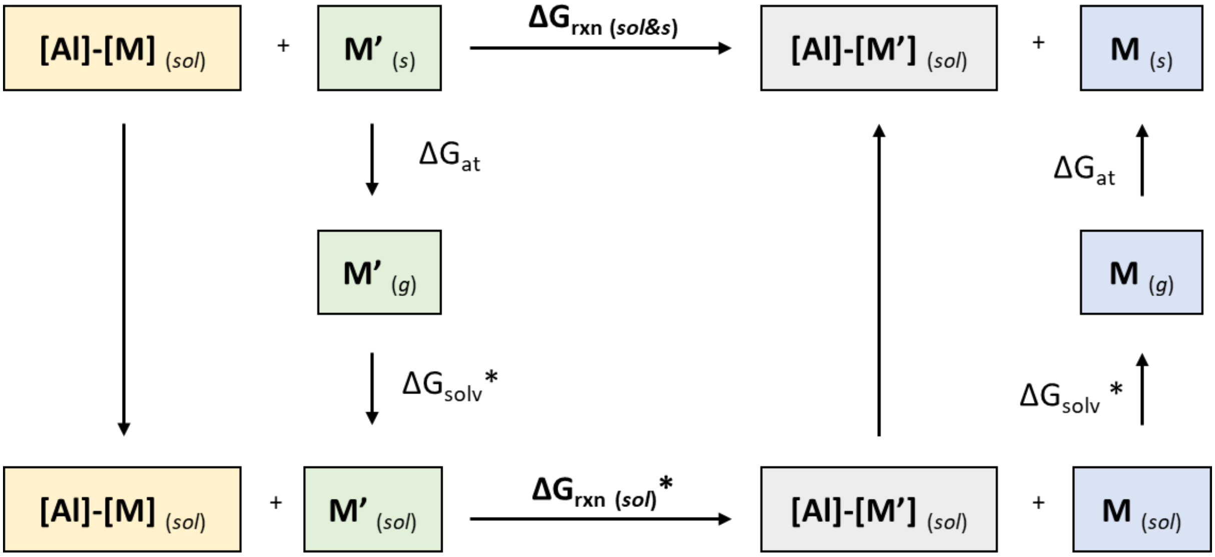

$$\Delta G_{rxn\ (sol\&s)} = 2\Delta G_{at}[M'^0] + 2\Delta G_{solv}[M'^0] + \Delta G_{rxn\ (sol)} - 2\Delta G_{at}[M^0] + 2\Delta G_{solv}[M^0] \quad (Eq. 1)$$

Figure S29. Combined computational Hess cycle with experimental data to calculate the reaction Gibbs energy, $\Delta G_{rxn\ (sol\&s)}$ (* = computed values).

We will now work through the above sequentially, using reaction 3 ($\mathbf{4^K} \rightarrow \mathbf{4}^{Rb}$) as our worked example.

$$\Delta G_{rxn\ (sol\&s)}\ [\mathbf{3}]$$
$$= 2\Delta G_{at}[Rb^0] + 2\Delta G_{solv}[Rb^0] + \Delta G_{rxn\ (sol)} - 2\Delta G_{at}[K^0] + 2\Delta G_{solv}[K^0] \quad (Eq. 2)$$

1.1. Computed Gibbs energy of the reaction in solution ($\Delta G_{rxn\ (sol)}$)

Once the Gibbs energies for the all the species involved in the reaction are obtained, the Gibbs energy of the reaction in solution can be calculated through Eq. 3.

TIP: Pay attention to the stoichiometry of the reaction: $[Al\text{-}K]_2$ (1), $[Al\text{-}Rb]_2$ (1), K^0 (2) and Rb^0 (2).

$$\Delta G_{rxn\ (sol)} = G_{(sol)}[AlRb]_2 + 2G_{(sol)}[K^0] - G_{(sol)}[AlK]_2 - 2G_{(sol)}[Rb^0] \ (Eq. 3)$$

For this reaction, the Gibbs energy in solution was 3.2 kcal mol^{-1} following method B.

1.2. Experimental Gibbs atomisation energy of M(0) atoms ($\Delta G_{at}[M^0]$)

The atomisation Gibbs energy for M^0 (K^0 and Rb^0) is calculated from the experimental enthalpies of formation (H_f) and entropies (S^o). The H_f and S for both alkali metals in different states can be found on CODATA.[18] First, the atomisation enthalpy (ΔH_{at}) and entropy (ΔS_{at}) are calculated through Eq. 4 and Eq. 5.

TIP: Pay attention to the units, H_f and S are usually expressed in kJ mol^{-1} and J mol^{-1} K^{-1}, respectively. Once ΔH_{at} and ΔS_{at} are calculated for both alkali metals, the atomisation Gibbs energy for each case can be calculated (Eq. 6). The atomisation Gibbs energies for K^0 and Rb^0 were 14.5 kcal mol^{-1} and 12.7 kcal mol^{-1}, respectively.

$$\Delta H_{at} = H_f\,[K^0{}_{(g)}] - H_f[K^0{}_{(s)}] \quad (Eq.4)$$

$$\Delta S_{at} = S\,[K^0{}_{(g)}] - S\,[K^0{}_{(s)}] \quad (Eq.5)$$

$$\Delta G_{at} = \Delta H_{at} - T\Delta S_{at} \quad (Eq.6)$$

1.3. Calculation of the solvation Gibbs energy for M(0) ($\Delta G_{solv}[M^0]$)

Solvation Gibbs energies are obtained implicitly for each metal by computing the electronic energy of M(0) in the gas phase and subtracting it from the single point energy that included solvation, dispersion (D3^{BJ}), and a larger basis set (ZORA-def2-TZVPP, with SARC-ZORA-TZVPP for Rb and larger elements). The solvation Gibbs energies for K^0 and Rb^0 are −0.03095 kcal mol^{-1} and −0.00799 kcal mol^{-1} respectively.

1.4. Calculation of the Gibbs energy for the reaction of interest ($\Delta G_{rxn\,(sol\&s)}$)

Once all the different Gibbs energies for the reaction have been obtained, the Gibbs energy for the reaction of interest can be calculated using Eq. 2 (Figure S29). The Gibbs energy for reaction **3** was −0.1 kcal mol^{-1} using method B.

Table S7. Summary of Gibbs energy (in kcal mol^{-1}) for reactions **1-6** following different methodologies (A-D) in solution and using a combined experimental and computational Hess cycle.

**Reaction**	**ΔG (*sol*)** / kcal mol^{-1}				**ΔG (*sol&s*)** / kcal mol^{-1}			
	**Method A**	**Method B**	**Method C**	**Method D**	**Method A**	**Method B**	**Method C**	**Method D**
**1**	3.6	8.9	6.6	11.4	−13.7	−14.3	−10.9	−10.2
**2**	-2.5	-0.7	−4.5	−5.4	−8.1	−8.6	−10.1	−12.4
**3**	7.4	3.4	6.9	8.2	4.5	−0.1	4.2	−0.9
**4**	−7.4	−3.4	−6.9	−8.2	−4.5	0.1	−4.2	0.9
**5**	−4.0	−1.4	−1.6	−1.3	−5.3	−3.1	−2.9	−2.2
**6**	3.3	1.9	5.3	6.9	−0.8	−3.2	−4.6	−3.0

References

1. R. J. Schwamm, M. P. Coles, M. S. Hill, M. F. Mahon, C. L. McMullin, N. A. Rajabi, A. S. S. Wilson, *Angew. Chem. Int. Ed.*, 2020, **59**, 3928-3932.
2. H.-Y. Liu, M. S. Hill, M. F. Mahon, C. L. McMullin and R. J. Schwamm, *Organometallics*, 2023, **42**, 2881-2892.
3. L. E. English, R. A. Jackson, N. J. Evans, D. Babula, H. Draper, S. Brown, J. Fletcher, D. J. Liptrot and K. G. Pearce, *RSC Mechanochem.*, 2025, Advance Article, DOI https://doi.org/10.1039/D5MR00039D.
4. O. V. Dolomanov, L. J. Bourhis, R. J. Gildea, J. A. K. Howard and H. Puschmann, *J. Appl. Cryst.* 2009, **42**, 339-341.
5. G. M. Sheldrick, *Acta Cryst.* 2015, **A71**, 3-8.
6. G. M. Sheldrick, *Acta Cryst*. 2015, **C71**, 3-8.
7. M. J. Frisch, G. W. Trucks, H. B. Schlegel, G. E. Scuseria, M. A. Robb, J. R. Cheeseman, G. Scalmani, V. Barone, G. A. Petersson, H. Nakatsuji, X. Li, M. Caricato, A. V. Marenich, J. Bloino, B. G. Janesko, R. Gomperts, B. Mennucci, H. P. Hratchian, J. V. Ortiz, A. F. Izmaylov, J. L. Sonnenberg, Williams, F. Ding, F. Lipparini, F. Egidi, J. Goings, B. Peng, A. Petrone, T. Henderson, D. Ranasinghe, V. G. Zakrzewski, J. Gao, N. Rega, G. Zheng, W. Liang, M. Hada, M. Ehara, K. Toyota, R. Fukuda, J. Hasegawa, M. Ishida, T. Nakajima, Y. Honda, O. Kitao, H. Nakai, T. Vreven, K. Throssell, J. A. Montgomery Jr., J. E. Peralta, F. Ogliaro, M. J. Bearpark, J. J. Heyd, E. N. Brothers, K. N. Kudin, V. N. Staroverov, T. A. Keith, R. Kobayashi, J. Normand, K. Raghavachari, A. P. Rendell, J. C. Burant, S. S. Iyengar, J. Tomasi, M. Cossi, J. M. Millam, M. Klene, C. Adamo, R. Cammi, J. W. Ochterski, R. L. Martin, K. Morokuma, O. Farkas, J. B. Foresman, D. J. Fox, Wallingford, CT, 2016.
8. a) A. D. Becke, *Phys. Rev. A* 1988, **38**, 3098-3100; b) J. P. Perdew, *Phys. Rev. B* 1986, **33**, 8822-8824.
9. S. Grimme, S. Ehrlich, L. Goerigk, *J. Comp. Chem*. 2011, **32**, 1456-1465.
10. J. Tomasi, B. Mennucci, R. Cammi, *Chem. Rev*. 2005, **105**, 2999-3094.
11. D. Andrae, U. Häußermann, M. Dolg, H. Stoll, H. Preuß, *Theor. Chim. Acta* 1990, **77**, 123-141.
12. a) P. C. Hariharan, J. A. Pople, *Theor. Chim. Acta* 1973, **28**, 213-222; b) W. J. Hehre, R. Ditchfield, J. A. Pople, *J. Chem. Phys.* 1972, **56**, 2257-2261.
13. A. Höllwarth; M. Böhme; S. Dapprich; A. W. Ehlers; A. Gobbi; V. Jonas; K. F. Köhler; R. Stegmann; A. Veldkamp; G. Frenking, *Chem. Phys. Lett.* 1993, **208**, 237.
14. a) R. Krishnan; J. S. Binkley; R. Seeger; J. A. Pople, *J. Chem. Phys.* 1980, **72**, 650-654; b) A. D. McLean; G. S. J. Chandler, *Chem. Phys.* 1980, **72**, 5639-5648; c) M. M. Francl; W. J Pietro; W. J. Hehre; J. S. Binkley; M. S. Gordon; D. J. DeFrees; J. A. Pople, *J. Chem. Phys.*1982 **77**, 3654-3665; c) T. Clark; J. Chandrasekhar; G. W. Spitznagel; P. v. R. Schleyer, *J. Comput. Chem.* 1983, **4**, 294-301; d) G. W. Spitznagel; T. Clark; P. v. R. Schleyer; J. W. Hehre, *J.*

Comput. Chem. 1987, **8**, 1109-1116. e) J.- M. Blaudeau; M. P. McGrath; L. A. Curtiss; L. Radom, J. Chem. Phys. 1997, **107**, 5016-5021.

15. a) T. Leininger; A. Nicklass; W. Küchle; H. Stoll, M. Dolg; A. Bergner, *Chem. Phys. Lett.* 1996, **255**, 274-280; b) F. Weigend; R. Ahlrichs, *Phys. Chem. Chem. Phys*. 2005 **7**, 3297.
16. a) D. A. Pantazis; X.-Y. Chen; C. R. Landis; F. Neese, *J. Chem. Theory Comput.* 2008, **4**, 908; b) M. Buhl; C. Reimann; D. A. Pantazis; T. Bredow; F. Neese, *J. Chem. Theory Comput.* 2008, **4**, 1449; c) D. A Pantazis; F. Neese, *J. Chem. Theory Comput.* 2009, **5**, 2229; d) D. A. Pantazis; F. Neese, *J. Chem. Theory Comput.* 2011, **7**, 677; e) D. A. Pantazis; F. Neese, *Theor. Chem. Acc.*, 2012, **131**, 1292; f) J. D. Rolfes; F. Neese; D. A. Pantazis, *J. Comput. Chem.* 2020, **41**, 1842.
17. F. Nesse, *IREs Comput Mol Sci. 2022*; **12**:e1606.
18. J. D. Cox, D. D. Wagman, V. A. Medvedav, in *CODATA Key Values for Thermodynamics*, Hemisphere Publishing Corp., New York, **1989**.

Computed Hartree Energies and Cartesian Coordinates

4^{Li}

Method A & B

SCF (BP86/BS1) Energy = -2600.70254436

Enthalpy 0K = -2599.268021

Enthalpy 298K = -2599.172131

Free Energy 298K = -2599.401113

Lowest Frequency = 10.1410 cm^{-1}

Second Frequency = 17.3134 cm^{-1}

SCF (BP86-$D3^{BJ}$) Energy = -2601.18706239

SCF (C_6H_6) Energy = -2600.71147739

SCF (BS2) Energy = -4224.67523217

SCF (BP86-$D3^{BJ}$(C_6H_6)/BS3) Energy = -4232.58121678

Si	1.78500	-4.07327	3.06409
Si	0.44355	-1.76092	5.97617
Al	0.62657	-0.89308	2.68086
N	0.88845	-2.77239	2.20285
N	0.70796	-0.64750	4.58804
C	1.09235	-4.40054	4.81095
H	1.49996	-5.39766	5.07962
H	-0.00407	-4.53783	4.75708
C	1.47253	-3.36683	5.89279
H	1.33312	-3.80948	6.90145
H	2.54727	-3.11134	5.83649
C	3.64018	-3.66812	3.32260
H	3.78650	-2.61578	3.61868
H	4.04296	-4.30048	4.13417
H	4.25542	-3.85174	2.42667
C	1.62265	-5.72152	2.11538
H	2.00172	-5.67375	1.08232
H	2.19482	-6.50251	2.64667
H	0.57124	-6.05307	2.06924
C	0.97026	-0.91221	7.60114
H	0.53768	0.09305	7.72211
H	0.65670	-1.52788	8.46254
H	2.06766	-0.80614	7.64672
C	-1.37395	-2.34693	6.13951
H	-1.79596	-2.65326	5.16697
H	-1.41895	-3.22346	6.81086
H	-2.03247	-1.57123	6.56185
C	0.33300	-3.05616	0.93128
C	-1.09968	-3.12438	0.76342
C	-1.64980	-3.33194	-0.52341
H	-2.73866	-3.37214	-0.63550
C	-0.83841	-3.50208	-1.65670
H	-1.27598	-3.64626	-2.64936
C	0.55894	-3.45544	-1.49755
H	1.19087	-3.58177	-2.38033
C	1.16074	-3.22066	-0.24210

C	-2.04558	-3.08601	1.96399
H	-1.44548	-2.76010	2.83187
C	-2.56596	-4.51045	2.27240
H	-1.73512	-5.21452	2.44657
H	-3.20320	-4.50308	3.17397
H	-3.16927	-4.90309	1.43440
C	-3.21590	-2.09766	1.79607
H	-3.88798	-2.38307	0.96735
H	-3.82630	-2.07057	2.71524
H	-2.84746	-1.07671	1.59992
C	2.68320	-3.12171	-0.14155
H	2.94874	-3.41444	0.88481
C	3.16425	-1.66380	-0.31277
H	2.65969	-0.99458	0.40790
H	4.25366	-1.59059	-0.14799
H	2.95067	-1.29556	-1.33122
C	3.43661	-4.06540	-1.10009
H	3.33621	-3.75655	-2.15493
H	4.51455	-4.05417	-0.86391
H	3.08145	-5.10694	-1.01649
C	0.93899	0.73874	4.91946
C	2.27157	1.26788	4.89815
C	2.48369	2.63692	5.15656
H	3.50697	3.03052	5.13370
C	1.42329	3.49898	5.46026
H	1.60830	4.55834	5.66897
C	0.12383	2.97929	5.51408
H	-0.70971	3.64446	5.77079
C	-0.14212	1.62053	5.25425
C	3.48747	0.38025	4.62747
H	3.11202	-0.65690	4.57409
C	4.14941	0.71275	3.27190
H	4.51614	1.75502	3.25252
H	5.01104	0.04898	3.07803
H	3.42882	0.59420	2.44222
C	4.52524	0.44779	5.77021
H	4.06891	0.20439	6.74442
H	5.34722	-0.26704	5.58827
H	4.97546	1.45276	5.85603
C	-1.58950	1.13426	5.35507
H	-1.58363	0.05908	5.11601
C	-2.50738	1.81838	4.32180
H	-2.14187	1.63615	3.29808
H	-3.53835	1.42842	4.39343
H	-2.55368	2.91155	4.48077
C	-2.16843	1.31604	6.77677
H	-2.21567	2.38318	7.05811
H	-3.19622	0.91427	6.83136
H	-1.56094	0.79950	7.53773
Li	-0.36337	-1.30891	-0.94692
Si	-1.78500	4.07327	-3.06409
Si	-0.44355	1.76092	-5.97617
Al	-0.62657	0.89308	-2.68086

```
N  -0.88845  2.77239 -2.20285
N  -0.70796  0.64750 -4.58804
C  -1.09235  4.40054 -4.81095
H  -1.49996  5.39766 -5.07962
H   0.00407  4.53783 -4.75708
C  -1.47253  3.36683 -5.89279
H  -1.33312  3.80948 -6.90145
H  -2.54727  3.11134 -5.83649
C  -3.64018  3.66812 -3.32260
H  -3.78650  2.61578 -3.61868
H  -4.04296  4.30048 -4.13417
H  -4.25542  3.85174 -2.42667
C  -1.62265  5.72152 -2.11538
H  -2.00172  5.67375 -1.08232
H  -2.19482  6.50251 -2.64667
H  -0.57124  6.05307 -2.06924
C  -0.97026  0.91221 -7.60114
H  -0.53768 -0.09305 -7.72211
H  -0.65670  1.52788 -8.46254
H  -2.06766  0.80614 -7.64672
C   1.37395  2.34693 -6.13951
H   1.79596  2.65326 -5.16697
H   1.41895  3.22346 -6.81086
H   2.03247  1.57123 -6.56185
C  -0.33300  3.05616 -0.93128
C   1.09968  3.12438 -0.76342
C   1.64980  3.33194  0.52341
H   2.73866  3.37214  0.63550
C   0.83841  3.50208  1.65670
H   1.27598  3.64626  2.64936
C  -0.55894  3.45544  1.49755
H  -1.19087  3.58177  2.38033
C  -1.16074  3.22066  0.24210
C   2.04558  3.08601 -1.96399
H   1.44548  2.76010 -2.83187
C   2.56596  4.51045 -2.27240
H   1.73512  5.21452 -2.44657
H   3.20320  4.50308 -3.17397
H   3.16927  4.90309 -1.43440
C   3.21590  2.09766 -1.79607
H   3.88798  2.38307 -0.96735
H   3.82630  2.07057 -2.71524
H   2.84746  1.07671 -1.59992
C  -2.68320  3.12171  0.14155
H  -2.94874  3.41444 -0.88481
C  -3.16425  1.66380  0.31277
H  -2.65969  0.99458 -0.40790
H  -4.25366  1.59059  0.14799
H  -2.95067  1.29556  1.33122
C  -3.43661  4.06540  1.10009
H  -3.33621  3.75655  2.15493
H  -4.51455  4.05417  0.86391
H  -3.08145  5.10694  1.01649
C  -0.93899 -0.73874 -4.91946
C  -2.27157 -1.26788 -4.89815
C  -2.48369 -2.63692 -5.15656
H  -3.50697 -3.03052 -5.13370
C  -1.42329 -3.49898 -5.46026
H  -1.60830 -4.55834 -5.66897
C  -0.12383 -2.97929 -5.51408
H   0.70971 -3.64446 -5.77079
C   0.14212 -1.62053 -5.25425
C  -3.48747 -0.38025 -4.62747
H  -3.11202  0.65690 -4.57409
C  -4.14941 -0.71275 -3.27190
H  -4.51614 -1.75502 -3.25252
H  -5.01104 -0.04898 -3.07803
H  -3.42882 -0.59420 -2.44222
C  -4.52524 -0.44779 -5.77021
H  -4.06891 -0.20439 -6.74442
H  -5.34722  0.26704 -5.58827
H  -4.97546 -1.45276 -5.85603
C   1.58950 -1.13426 -5.35507
H   1.58363 -0.05908 -5.11601
C   2.50738 -1.81838 -4.32180
H   2.14187 -1.63615 -3.29808
H   3.53835 -1.42842 -4.39343
H   2.55368 -2.91155 -4.48077
C   2.16843 -1.31604 -6.77677
H   2.21567 -2.38318 -7.05811
H   3.19622 -0.91427 -6.83136
H   1.56094 -0.79950 -7.53773
Li  0.36337  1.30891  0.94692
```

Method C + D
SCF (BP86/BS4) Energy = -4221.59306500
Enthalpy 0K = -4220.167277
Enthalpy 298K = -4220.071289
Free Energy 298K = -4220.302707
Lowest Frequency = 5.6103 cm^{-1}
Second Frequency = 13.4649 cm^{-1}
SCF (BP86-$D3^{BJ}$) Energy = -4222.08113535
SCF (PCM=C_6H_6) Energy = -4221.601678
SCF (SMD=C_6H_6) Energy = -4221.623735
SCF (BS5) Energy = -4224.97550728

```
Si  1.80774 -4.05689  3.06738
Si  0.34362 -1.77321  5.92678
Al  0.59338 -0.91287  2.67439
N   0.88162 -2.78173  2.21034
N   0.65103 -0.64604  4.56925
C   1.15794 -4.38400  4.82510
H   1.64262 -5.35103  5.10182
H   0.06917 -4.61058  4.78157
C   1.45955 -3.31718  5.89721
H   1.32733 -3.75582  6.91502
H   2.52532 -2.99812  5.85849
C   3.65981 -3.64545  3.30319
```

H	3.81060	-2.59382	3.62373
H	4.07821	-4.29836	4.09948
H	4.27205	-3.80699	2.39262
C	1.65805	-5.70976	2.13867
H	2.02847	-5.66659	1.09493
H	2.25071	-6.48297	2.67308
H	0.60560	-6.06147	2.10203
C	0.68984	-0.91384	7.58441
H	0.07296	-0.00643	7.73868
H	0.47682	-1.61686	8.41803
H	1.75346	-0.60595	7.66320
C	-1.43960	-2.46145	5.95280
H	-1.75816	-2.82267	4.95306
H	-1.49170	-3.32601	6.64966
H	-2.18840	-1.71769	6.29385
C	0.34735	-3.05067	0.92938
C	-1.08676	-3.09189	0.73351
C	-1.61696	-3.30181	-0.56180
H	-2.70922	-3.32826	-0.69671
C	-0.78772	-3.48831	-1.68103
H	-1.21458	-3.63833	-2.68450
C	0.60642	-3.45010	-1.49975
H	1.25628	-3.57946	-2.37671
C	1.19088	-3.21497	-0.23466
C	-2.04888	-3.02147	1.91906
H	-1.51086	-2.49682	2.73863
C	-2.36055	-4.44780	2.42484
H	-1.43433	-4.99177	2.69884
H	-3.01663	-4.41547	3.32020
H	-2.88102	-5.03987	1.64179
C	-3.34259	-2.23864	1.63950
H	-3.99939	-2.75248	0.90562
H	-3.93066	-2.12921	2.57425
H	-3.12710	-1.22147	1.25293
C	2.71088	-3.09316	-0.12791
H	2.97656	-3.35615	0.91171
C	3.16439	-1.63195	-0.32869
H	2.64677	-0.95421	0.38483
H	4.25816	-1.53045	-0.16468
H	2.94061	-1.28266	-1.35850
C	3.48488	-4.04756	-1.05543
H	3.38671	-3.76762	-2.12541
H	4.56812	-4.01592	-0.81499
H	3.14402	-5.09841	-0.94815
C	0.95580	0.72201	4.89392
C	2.30010	1.21382	4.77175
C	2.57636	2.57180	5.04132
H	3.61311	2.93643	4.94858
C	1.57079	3.46102	5.43912
H	1.80664	4.51623	5.65127
C	0.25996	2.98176	5.58211
H	-0.53168	3.67447	5.91097
C	-0.06849	1.63601	5.32468
C	3.46995	0.29667	4.41123
H	3.03054	-0.70029	4.19671
C	4.22133	0.75895	3.14721
H	4.69907	1.75205	3.29058
H	5.02607	0.04105	2.88020
H	3.53151	0.83784	2.28033
C	4.43894	0.12226	5.60067
H	3.91072	-0.25169	6.50180
H	5.24493	-0.60121	5.35240
H	4.92453	1.08344	5.87479
C	-1.51987	1.18349	5.49566
H	-1.50100	0.08042	5.57474
C	-2.36415	1.50739	4.24848
H	-1.90217	1.07329	3.33655
H	-3.39392	1.10048	4.34237
H	-2.44923	2.60594	4.10126
C	-2.19671	1.73148	6.76708
H	-2.36291	2.82871	6.71394
H	-3.19349	1.26199	6.90762
H	-1.59440	1.52978	7.67694
Li	-0.31321	-1.27923	-0.93198
Si	-1.80774	4.05689	-3.06738
Si	-0.34362	1.77321	-5.92678
Al	-0.59338	0.91287	-2.67439
N	-0.88162	2.78173	-2.21034
N	-0.65103	0.64604	-4.56925
C	-1.15794	4.38400	-4.82510
H	-1.64262	5.35103	-5.10182
H	-0.06917	4.61058	-4.78157
C	-1.45955	3.31718	-5.89721
H	-1.32733	3.75582	-6.91502
H	-2.52532	2.99812	-5.85849
C	-3.65981	3.64545	-3.30319
H	-3.81060	2.59382	-3.62373
H	-4.07821	4.29836	-4.09948
H	-4.27205	3.80699	-2.39262
C	-1.65805	5.70976	-2.13867
H	-2.02847	5.66659	-1.09493
H	-2.25071	6.48297	-2.67308
H	-0.60560	6.06147	-2.10203
C	-0.68984	0.91384	-7.58441
H	-0.07296	0.00643	-7.73868
H	-0.47682	1.61686	-8.41803
H	-1.75346	0.60595	-7.66320
C	1.43960	2.46145	-5.95280
H	1.75816	2.82267	-4.95306
H	1.49170	3.32601	-6.64966
H	2.18840	1.71769	-6.29385
C	-0.34735	3.05067	-0.92938
C	1.08676	3.09189	-0.73351
C	1.61696	3.30181	0.56180
H	2.70922	3.32826	0.69671
C	0.78772	3.48831	1.68103
H	1.21458	3.63833	2.68450
C	-0.60642	3.45010	1.49975
H	-1.25628	3.57946	2.37671
C	-1.19088	3.21497	0.23466
C	2.04888	3.02147	-1.91906
H	1.51086	2.49682	-2.73863

C 2.36055 4.44780 -2.42484
H 1.43433 4.99177 -2.69884
H 3.01663 4.41547 -3.32020
H 2.88102 5.03987 -1.64179
C 3.34259 2.23864 -1.63950
H 3.99939 2.75248 -0.90562
H 3.93066 2.12921 -2.57425
H 3.12710 1.22147 -1.25293
C -2.71088 3.09316 0.12791
H -2.97656 3.35615 -0.91171
C -3.16439 1.63195 0.32869
H -2.64677 0.95421 -0.38483
H -4.25816 1.53045 0.16468
H -2.94061 1.28266 1.35850
C -3.48488 4.04756 1.05543
H -3.38671 3.76762 2.12541
H -4.56812 4.01592 0.81499
H -3.14402 5.09841 0.94815
C -0.95580 -0.72201 -4.89392
C -2.30010 -1.21382 -4.77175
C -2.57636 -2.57180 -5.04132
H -3.61311 -2.93643 -4.94858
C -1.57079 -3.46102 -5.43912
H -1.80664 -4.51623 -5.65127
C -0.25996 -2.98176 -5.58211
H 0.53168 -3.67447 -5.91097
C 0.06849 -1.63601 -5.32468
C -3.46995 -0.29667 -4.41123
H -3.03054 0.70029 -4.19671
C -4.22133 -0.75895 -3.14721
H -4.69907 -1.75205 -3.29058
H -5.02607 -0.04105 -2.88020
H -3.53151 -0.83784 -2.28033
C -4.43894 -0.12226 -5.60067
H -3.91072 0.25169 -6.50180
H -5.24493 0.60121 -5.35240
H -4.92453 -1.08344 -5.87479
C 1.51987 -1.18349 -5.49566
H 1.50100 -0.08042 -5.57474
C 2.36415 -1.50739 -4.24848
H 1.90217 -1.07329 -3.33655
H 3.39392 -1.10048 -4.34237
H 2.44923 -2.60594 -4.10126
C 2.19671 -1.73148 -6.76708
H 2.36291 -2.82871 -6.71394
H 3.19349 -1.26199 -6.90762
H 1.59440 -1.52978 -7.67694
Li 0.31321 1.27923 0.93198

Li(0)
Method A & B
SCF (BP86/BS1) Energy = -7.47902967105
Enthalpy 0K = -7.479030
Enthalpy 298K = -7.476669
Free Energy 298K = -7.492432
SCF (BP86-$D3^{BJ}$) Energy = -7.47902967105
SCF (C_6H_6) Energy = -7.48809027803
SCF (BS2) Energy = -7.47933192055
SCF (BP86-$D3^{BJ}$(C_6H_6)/BS3) Energy = -7.48312598

Li 0.00000 0.00000 0.00000

Method C & D
SCF (BP86/BS4) Energy = -7.47319055602
Enthalpy 0K = -7.473191
Enthalpy 298K = -7.470830
Free Energy 298K = -7.486593
SCF (BP86-$D3^{BJ}$) Energy = -7.47319055602
SCF (PCM=C_6H_6) Energy = -7.483692
SCF (SMD=C_6H_6) Energy = -7.474213
SCF (BS5) Energy = -7.48081937506

Li 0.00000 0.00000 0.00000

4^{Na}
Method A & B
SCF (BP86/BS1) Energy = -2586.08782263
Enthalpy 0K = -2584.657160
Enthalpy 298K = -2584.559376
Free Energy 298K = -2584.796723
Lowest Frequency = 4.9857 cm^{-1}
Second Frequency = 11.1229 cm^{-1}
SCF (BP86-$D3^{BJ}$) Energy = -2586.58338675
SCF (C_6H_6) Energy = -2586.09831909
SCF (BS2) Energy = -4534.23907500
SCF (BP86-$D3^{BJ}$(C_6H_6)/BS3) Energy = -4542.84862586

Na 0.32053 1.76070 0.14887
Si -5.24008 2.27897 1.33793
Si -5.88431 -0.54302 -1.35368
Al -2.89506 0.14780 0.09398
N -3.61189 1.83981 0.72331
N -4.28782 -0.95428 -0.64301
C -2.56476 2.80798 0.79915
C -2.20145 3.56396 -0.36937
C -1.06041 4.39414 -0.34105
H -0.77810 4.94777 -1.24386
C -0.28585 4.53944 0.82083
H 0.60456 5.17492 0.81752
C -0.67171 3.85119 1.98210
H -0.08644 3.98027 2.90022
C -1.79574 2.99688 2.00007

C	-3.04547	3.50554	-1.64277
H	-3.93705	2.90481	-1.39224
C	-3.51883	4.90855	-2.08395
H	-4.04939	5.43059	-1.26998
H	-4.20580	4.82761	-2.94436
H	-2.67358	5.54791	-2.39572
C	-2.30904	2.79077	-2.79562
H	-1.38146	3.32481	-3.07021
H	-2.94503	2.73806	-3.69693
H	-2.03313	1.75771	-2.51378
C	-2.15319	2.28887	3.30633
H	-3.06446	1.70267	3.10864
C	-2.45282	3.28928	4.44553
H	-1.56034	3.88484	4.70872
H	-2.76955	2.74987	5.35553
H	-3.25516	3.99448	4.17203
C	-1.05797	1.29191	3.73564
H	-0.89791	0.53014	2.95294
H	-1.34721	0.76789	4.66367
H	-0.09464	1.79914	3.92542
C	-5.69972	1.31558	2.92555
H	-6.78240	1.40947	3.12461
H	-5.16472	1.69062	3.81357
H	-5.47090	0.24099	2.82315
C	-5.30114	4.15013	1.70236
H	-5.22978	4.73805	0.77137
H	-4.48436	4.47840	2.36514
H	-6.25818	4.40671	2.18943
C	-5.72200	0.50782	-2.94388
H	-6.71088	0.90169	-3.24036
H	-5.33000	-0.08100	-3.78913
H	-5.05011	1.36968	-2.79395
C	-6.83560	-2.13889	-1.78402
H	-7.11551	-2.69771	-0.87507
H	-6.24601	-2.81798	-2.42075
H	-7.76487	-1.88483	-2.32364
C	-3.86424	-2.32351	-0.75836
C	-3.18755	-2.80102	-1.93122
C	-2.76961	-4.14666	-1.99247
H	-2.26559	-4.50603	-2.89798
C	-3.00158	-5.03716	-0.93719
H	-2.68401	-6.08321	-1.01261
C	-3.65671	-4.57364	0.21097
H	-3.84504	-5.26732	1.03885
C	-4.09422	-3.23948	0.32197
C	-2.93936	-1.90562	-3.14736
H	-3.33772	-0.90841	-2.89662
C	-3.68461	-2.42354	-4.39946
H	-3.30493	-3.41208	-4.71434
H	-3.54818	-1.72964	-5.24807
H	-4.76657	-2.52833	-4.21463
C	-1.43716	-1.73691	-3.45833
H	-0.90747	-1.25227	-2.61769
H	-1.29040	-1.09964	-4.34835
H	-0.94908	-2.70711	-3.65916
C	-4.80760	-2.79616	1.60008
H	-5.23712	-1.80130	1.38815
C	-5.96117	-3.73881	2.00509
H	-6.68102	-3.87542	1.18056
H	-6.50731	-3.32588	2.87132
H	-5.59522	-4.73941	2.29706
C	-3.80640	-2.62969	2.76597
H	-4.31833	-2.28725	3.68330
H	-3.02476	-1.88874	2.51366
H	-3.30280	-3.58659	2.99502
Na	-0.30205	-1.74554	0.14023
Si	5.24536	-2.23617	1.37831
Si	5.88604	0.56110	-1.33641
Al	2.89439	-0.15184	0.11097
N	3.61588	-1.84236	0.74173
N	4.28243	0.95599	-0.63019
C	2.58636	-2.83038	0.75077
C	1.82366	-3.11804	1.93644
C	0.69206	-3.95704	1.85219
H	0.10595	-4.15379	2.75729
C	0.29929	-4.54921	0.64050
H	-0.59760	-5.17348	0.58849
C	1.07851	-4.32504	-0.50490
H	0.79802	-4.81158	-1.44632
C	2.22329	-3.49918	-0.47014
C	2.19338	-2.51747	3.29191
H	3.18934	-2.06236	3.17566
C	2.27421	-3.57787	4.41186
H	2.94580	-4.41044	4.14268
H	2.65428	-3.11985	5.34166
H	1.28405	-4.00946	4.64293
C	1.22738	-1.38242	3.68866
H	0.19250	-1.75662	3.79261
H	1.52024	-0.93346	4.65434
H	1.23117	-0.58354	2.92592
C	3.08360	-3.37608	-1.72771
H	3.93677	-2.73148	-1.45413
C	3.64276	-4.75124	-2.15933
H	2.83646	-5.43886	-2.47164
H	4.33113	-4.63530	-3.01478
H	4.19603	-5.23712	-1.33810
C	2.33662	-2.69939	-2.89573
H	1.99482	-1.68587	-2.61722
H	2.99305	-2.60536	-3.77872
H	1.44931	-3.28287	-3.20058
C	5.32741	-4.08729	1.82690
H	6.30864	-4.32051	2.27606
H	4.54766	-4.37992	2.54828
H	5.20777	-4.72066	0.93146
C	5.68911	-1.18946	2.91738
H	5.43650	-0.12672	2.76267
H	5.16574	-1.52937	3.82633
H	6.77425	-1.24993	3.11589
C	6.82895	2.16843	-1.74287
H	7.76370	1.92696	-2.27879
H	6.23837	2.84881	-2.37750
H	7.09864	2.72052	-0.82683

```
C   5.75075 -0.46744 -2.94377
H   5.10012 -1.34827 -2.81339
H   5.35017  0.12550 -3.78188
H   6.75097 -0.83199 -3.23997
C   3.83746  2.31759 -0.75920
C   4.06086  3.25002  0.30852
C   3.59404  4.57345  0.18908
H   3.77713  5.27942  1.00764
C   2.91523  5.01105 -0.95554
H   2.57242  6.04862 -1.03664
C   2.69317  4.10595 -2.00029
H   2.17382  4.44588 -2.90460
C   3.14160  2.77020 -1.93098
C   4.79612  2.83404  1.58333
H   5.25951  1.85474  1.37017
C   5.91691  3.81690  1.98405
H   5.51680  4.80183  2.28441
H   6.48421  3.42019  2.84418
H   6.62500  3.98413  1.15500
C   3.80592  2.63263  2.75305
H   3.04751  1.86717  2.50248
H   4.33209  2.30559  3.66789
H   3.27241  3.57247  2.98507
C   2.91124  1.86237 -3.14130
H   3.32045  0.87201 -2.88076
C   3.66062  2.38309 -4.39004
H   4.73889  2.50664 -4.19536
H   3.54324  1.68180 -5.23542
H   3.26822  3.36361 -4.71433
C   1.41436  1.67302 -3.46500
H   1.28437  1.02019 -4.34625
H   0.87951  1.19689 -2.62277
H   0.91823  2.63425 -3.68803
C   7.01016 -0.44009 -0.15884
H   7.12343  0.11529  0.79145
H   8.00748 -0.38860 -0.64404
C   6.63824 -1.91615  0.10978
H   6.40241 -2.44238 -0.83463
H   7.51498 -2.45250  0.52988
C  -7.00693  0.45109 -0.16874
H  -7.10532 -0.09969  0.78595
H  -8.00958  0.39191 -0.64172
C  -6.63747  1.92992  0.08414
H  -6.41181  2.44807 -0.86720
H  -7.51187  2.46854  0.50654
```

Method C & D

SCF (BP86/BS4) Energy = -4531.06159094

Enthalpy 0K = -4529.638470

Enthalpy 298K = -4529.541179

Free Energy 298K = -4529.776106

Lowest Frequency = 6.5395 cm^{-1}

Second Frequency = 12.4047 cm^{-1}

SCF (BP86-D3^{BJ}) Energy = -4531.559080

SCF (PCM=C_6H_6) Energy = -4531.070453

SCF (SMD=C_6H_6) Energy = -4531.092240

SCF (BS5) Energy = -4534.546354

```
Na  0.29857  1.65594  0.11455
Si  5.93175  0.39043 -1.24946
Si  5.09820 -2.33508  1.46795
Al  2.89140 -0.19249  0.10750
N   4.32824  0.84871 -0.59561
N   3.51577 -1.89743  0.76292
C   3.94796  2.22564 -0.75533
C   4.20524  3.16970  0.29766
C   3.83207  4.51896  0.13253
H   4.04528  5.23892  0.93948
C   3.20769  4.96910 -1.03977
H   2.93799  6.03116 -1.15713
C   2.94345  4.05170 -2.06488
H   2.45936  4.40381 -2.99142
C   3.30304  2.69073 -1.95254
C   4.86913  2.73192  1.60432
H   5.33826  1.74725  1.39712
C   5.97470  3.69027  2.08750
H   6.72901  3.87838  1.29567
H   6.50186  3.26137  2.96578
H   5.56931  4.67539  2.40358
C   3.81347  2.50942  2.70873
H   3.26884  3.45138  2.93615
H   4.28241  2.15052  3.64998
H   3.05801  1.75470  2.39401
C   3.03103  1.76586 -3.14146
H   3.40685  0.76313 -2.85461
C   3.79337  2.21808 -4.40585
H   3.42622  3.19918 -4.77650
H   3.66258  1.48365 -5.22911
H   4.88045  2.32440 -4.21291
C   1.52687  1.61806 -3.44286
H   0.98781  1.17272 -2.57855
H   1.35990  0.94748 -4.31239
H   1.05310  2.59456 -3.67957
C   5.81035 -0.63660 -2.85177
H   6.81533 -1.02327 -3.12809
H   5.43723 -0.03675 -3.70636
H   5.13670 -1.51021 -2.73430
C   6.94050  1.95345 -1.63629
H   7.16931  2.53220 -0.71736
H   6.41026  2.63548 -2.33199
H   7.90597  1.66881 -2.10702
C   5.52173 -1.28174  3.00054
H   6.59137 -1.41622  3.27104
H   4.91764 -1.55295  3.89078
H   5.35931 -0.20123  2.80411
C   5.10182 -4.17453  1.94276
H   4.98053 -4.81984  1.04738
H   4.29299 -4.43778  2.65372
```

H	6.07003	-4.43805	2.41973
C	2.45311	-2.84456	0.72726
C	1.65460	-3.13429	1.89224
C	0.50382	-3.94380	1.76808
H	-0.11109	-4.14427	2.66037
C	0.11855	-4.49832	0.53550
H	-0.79642	-5.10393	0.45388
C	0.92490	-4.27035	-0.59060
H	0.64501	-4.73081	-1.55241
C	2.09251	-3.47702	-0.51703
C	2.00004	-2.55355	3.26311
H	3.03777	-2.17548	3.19348
C	1.95097	-3.59958	4.39437
H	0.91626	-3.94976	4.59669
H	2.33415	-3.16176	5.34013
H	2.56594	-4.49296	4.15953
C	1.11241	-1.33894	3.59475
H	1.20701	-0.55785	2.80995
H	1.40167	-0.88327	4.56558
H	0.04159	-1.62868	3.66551
C	2.97831	-3.35409	-1.75716
H	3.84161	-2.72640	-1.45397
C	3.52874	-4.72975	-2.19113
H	4.05731	-5.23723	-1.35806
H	4.24641	-4.61507	-3.03093
H	2.71928	-5.40836	-2.53611
C	2.27221	-2.64376	-2.92802
H	2.95575	-2.53725	-3.79671
H	1.93429	-1.62566	-2.63587
H	1.38037	-3.20853	-3.27465
Na	-0.29918	-1.65705	0.11353
Si	-5.93160	-0.39027	-1.24989
Si	-5.09860	2.33503	1.46800
Al	-2.89152	0.19253	0.10783
N	-4.32815	-0.84856	-0.59587
N	-3.51601	1.89738	0.76338
C	-3.94769	-2.22542	-0.75574
C	-3.30251	-2.69025	-1.95291
C	-2.94266	-4.05115	-2.06535
H	-2.45838	-4.40306	-2.99186
C	-3.20692	-4.96871	-1.04040
H	-2.93705	-6.03072	-1.15787
C	-3.83157	-4.51884	0.13185
H	-4.04481	-5.23893	0.93868
C	-4.20498	-3.16965	0.29709
C	-3.03072	-1.76527	-3.14180
H	-3.40689	-0.76266	-2.85497
C	-3.79292	-2.21776	-4.40618
H	-4.87995	-2.32456	-4.21320
H	-3.66249	-1.48323	-5.22941
H	-3.42537	-3.19868	-4.77691
C	-1.52662	-1.61695	-3.44325
H	-1.05231	-2.59341	-3.67905
H	-1.36001	-0.94710	-4.31342
H	-0.98776	-1.17040	-2.57944
C	-4.86919	-2.73216	1.60369

H	-5.33842	-1.74753	1.39653
C	-5.97472	-3.69075	2.08650
H	-5.56925	-4.67584	2.40252
H	-6.50213	-3.26205	2.96473
H	-6.72884	-3.87885	1.29447
C	-3.81379	-2.50966	2.70835
H	-3.05838	-1.75478	2.39389
H	-4.28299	-2.15096	3.64955
H	-3.26907	-3.45158	2.93575
C	-6.94022	-1.95330	-1.63703
H	-7.90559	-1.66868	-2.10795
H	-6.40980	-2.63527	-2.33266
H	-7.16920	-2.53212	-0.71819
C	-5.81004	0.63694	-2.85207
H	-5.13635	1.51051	-2.73445
H	-5.43689	0.03718	-3.70671
H	-6.81498	1.02370	-3.12840
C	-5.10234	4.17449	1.94279
H	-6.07067	4.43802	2.41952
H	-4.29369	4.43776	2.65393
H	-4.98085	4.81977	1.04742
C	-5.52254	1.28166	3.00047
H	-5.35996	0.20117	2.80408
H	-4.91875	1.55290	3.89090
H	-6.59227	1.41604	3.27063
C	-2.45319	2.84434	0.72807
C	-2.09212	3.47674	-0.51611
C	-0.92417	4.26960	-0.58944
H	-0.64390	4.72996	-1.55117
C	-0.11793	4.49717	0.53682
H	0.79735	5.10234	0.45537
C	-0.50373	3.94285	1.76932
H	0.11105	4.14309	2.66175
C	-1.65489	3.13384	1.89325
C	-2.97779	3.35426	-1.75639
H	-3.84138	2.72688	-1.45339
C	-3.52763	4.73019	-2.19027
H	-2.71786	5.40852	-2.53506
H	-4.24523	4.61590	-3.03017
H	-4.05610	5.23779	-1.35720
C	-2.27182	2.64381	-2.92725
H	-1.93437	1.62552	-2.63521
H	-2.95527	2.53771	-3.79605
H	-1.37969	3.20824	-3.27365
C	-2.00104	2.55355	3.26413
H	-3.03874	2.17548	3.19409
C	-1.95254	3.60005	4.39500
H	-2.56737	4.49335	4.15946
H	-2.33622	3.16263	5.34073
H	-0.91794	3.95030	4.59769
C	-1.11364	1.33907	3.59685
H	-1.40344	0.88394	4.56777
H	-1.20778	0.55753	2.81247
H	-0.04284	1.62880	3.66805
C	-6.54575	2.09072	0.25300
H	-6.32485	2.64045	-0.69001

H -7.38806 2.65369 0.72208
C -6.98418 0.64098 -0.04131
H -7.11353 0.06610 0.90351
H -7.99482 0.64002 -0.51553
C 6.54563 -2.09075 0.25330
H 6.32491 -2.64042 -0.68979
H 7.38783 -2.65376 0.72252
C 6.98415 -0.64100 -0.04085
H 7.11340 -0.06620 0.90403
H 7.99485 -0.64004 -0.51493

Na(0)
Method A & B
SCF (BP86/BS1) Energy = -0.190251935461
Enthalpy 0K = -0.190252
Enthalpy 298K = -0.187891
Free Energy 298K = -0.205335
SCF (BP86-$D3^{BJ}$) Energy = -0.190252
SCF (C_6H_6) Energy = -0.194926
SCF (BS2) Energy = -162.278274773
SCF (BP86-$D3^{BJ}$(C_6H_6)/BS3) Energy = -162.62744579

Na 0.00000 0.00000 0.00000

Method C & D
SCF (BP86/BS4) Energy = -162.221825680
Enthalpy 0K = -162.221826
Enthalpy 298K = -162.219465
Free Energy 298K = -162.236909
SCF (BP86-$D3^{BJ}$) Energy = -162.221825680
SCF (PCM=C_6H_6) Energy = -162.226635
SCF (SMD=C_6H_6) Energy = -162.220913
SCF (BS5) Energy = -162.282753971

Na 0.00000 0.00000 0.00000

4^K
Method A & B
SCF (BP86/BS1) Energy = -2642.35511332
Enthalpy 0K = -2640.924883
Enthalpy 298K = -2640.827075
Free Energy 298K = -2641.063220
Lowest Frequency = 12.6229 cm^{-1}
Second Frequency = 16.0088 cm^{-1}
SCF (BP86-$D3^{BJ}$) Energy = -2642.841997
SCF (C_6H_6) Energy = -2642.360885
SCF (BS2) Energy = -5409.62525
SCF (BP86-$D3^{BJ}$(C_6H_6)/BS3) Energy = -5424.87168055

K 0.00020 2.24091 0.00016
Si 5.78728 1.43867 -1.35743
Si 5.78742 -1.43954 1.35705
Al 3.00297 0.00022 0.00058
N 4.12416 1.43992 -0.68962
N 4.12421 -1.44012 0.68943
C 3.36341 2.64477 -0.80496
C 3.27844 3.55091 0.30831
C 2.46108 4.69641 0.22202
H 2.40983 5.38064 1.07735
C 1.73577 4.99554 -0.94063
H 1.12402 5.90204 -0.99823
C 1.82550 4.12524 -2.03657
H 1.27531 4.36175 -2.95566
C 2.61901 2.95849 -1.99534
C 4.06840 3.30139 1.59345
H 4.74644 2.45734 1.37761
C 4.92659 4.51679 2.00626
H 5.59175 4.83860 1.18741
H 5.55479 4.26384 2.87821
H 4.30494 5.38451 2.29137
C 3.14222 2.86787 2.75216
H 2.40285 3.65576 2.98809
H 3.72390 2.66878 3.66979
H 2.59029 1.94482 2.49340
C 2.66213 2.06661 -3.23730
H 3.31130 1.21084 -2.98941
C 3.26799 2.80285 -4.45386
H 2.64397 3.66219 -4.75857
H 3.34096 2.12250 -5.32081
H 4.27834 3.18683 -4.23620
C 1.27127 1.49791 -3.59305
H 0.87161 0.88664 -2.76432
H 1.33268 0.84763 -4.48338
H 0.54414 2.29928 -3.81940
C 5.93263 0.37592 -2.94173
H 6.99556 0.22136 -3.20071
H 5.44495 0.85436 -3.80693
H 5.47366 -0.61793 -2.80418
C 6.32342 3.22273 -1.77247
H 6.43523 3.82882 -0.85731
H 5.59907 3.73846 -2.42356
H 7.29884 3.21206 -2.28980
C 5.93330 -0.37739 2.94173
H 6.99633 -0.22366 3.20080
H 5.44524 -0.85579 3.80675
H 5.47502 0.61683 2.80452
C 6.32316 -3.22384 1.77152
H 6.43465 -3.82984 0.85626
H 5.59874 -3.73943 2.42265
H 7.29864 -3.21354 2.28873
C 3.36318 -2.64479 0.80486
C 2.61881 -2.95836 1.99530

C	1.82512	-4.12500	2.03662
H	1.27498	-4.36141	2.95577
C	1.73519	-4.99533	0.94073
H	1.12332	-5.90174	0.99843
C	2.46043	-4.69634	-0.22200
H	2.40903	-5.38059	-1.07729
C	3.27793	-3.55096	-0.30839
C	2.66222	-2.06654	3.23731
H	3.31132	-1.21074	2.98929
C	3.26841	-2.80287	4.45364
H	2.64445	-3.66223	4.75844
H	3.34162	-2.12259	5.32063
H	4.27870	-3.18685	4.23572
C	1.27145	-1.49792	3.59353
H	0.87143	-0.88672	2.76493
H	1.33319	-0.84757	4.48379
H	0.54448	-2.29933	3.82023
C	4.06773	-3.30149	-1.59362
H	4.74643	-2.45802	-1.37760
C	4.92488	-4.51729	-2.00735
H	5.59003	-4.84005	-1.18887
H	5.55303	-4.26428	-2.87932
H	4.30255	-5.38439	-2.29283
C	3.14142	-2.86673	-2.75178
H	3.72288	-2.66758	-3.66954
H	2.59024	-1.94342	-2.49236
H	2.40141	-3.65400	-2.98780
K	0.00008	-2.24060	-0.00010
Si	-5.78759	-1.43904	-1.35734
Si	-5.78716	1.43927	1.35703
Al	-3.00305	-0.00008	-0.00011
N	-4.12448	-1.43996	-0.68955
N	-4.12397	1.44019	0.68941
C	-3.36357	-2.64474	-0.80471
C	-2.61922	-2.95864	-1.99508
C	-1.82559	-4.12532	-2.03612
H	-1.27547	-4.36198	-2.95522
C	-1.73566	-4.99536	-0.94000
H	-1.12380	-5.90180	-0.99746
C	-2.46089	-4.69604	0.22265
H	-2.40947	-5.38005	1.07815
C	-3.27839	-3.55063	0.30874
C	-2.66250	-2.06706	-3.23726
H	-3.31172	-1.21126	-2.98954
C	-3.26835	-2.80361	-4.45361
H	-4.27868	-3.18760	-4.23585
H	-3.34139	-2.12348	-5.32072
H	-2.64430	-3.66301	-4.75815
C	-1.27168	-1.49834	-3.59321
H	-0.54454	-2.29973	-3.81946
H	-1.33321	-0.84824	-4.48367
H	-0.87203	-0.88688	-2.76463
C	-4.06828	-3.30091	1.59387
H	-4.74649	-2.45705	1.37786
C	-4.92619	-4.51637	2.00710
H	-4.30435	-5.38389	2.29239
H	-5.55436	-4.26329	2.87904
H	-5.59136	-4.83853	1.18840
C	-3.14204	-2.86691	2.75236
H	-2.59025	-1.94389	2.49325
H	-3.72366	-2.66759	3.66999
H	-2.40255	-3.65463	2.98848
C	-6.32356	-3.22324	-1.77199
H	-7.29895	-3.21277	-2.28938
H	-5.59913	-3.73902	-2.42295
H	-6.43535	-3.82915	-0.85672
C	-5.93305	-0.37659	-2.94185
H	-5.47399	0.61725	-2.80449
H	-5.44548	-0.85516	-3.80704
H	-6.99600	-0.22198	-3.20074
C	-6.32317	3.22344	1.77179
H	-7.29861	3.21285	2.28910
H	-5.59884	3.73927	2.42282
H	-6.43498	3.82938	0.85653
C	-5.93289	0.37671	2.94143
H	-5.47355	-0.61702	2.80427
H	-5.44576	0.85545	3.80678
H	-6.99590	0.22186	3.19993
C	-3.36306	2.64490	0.80487
C	-3.27776	3.55102	-0.30841
C	-2.46034	4.69645	-0.22198
H	-2.40887	5.38066	-1.07732
C	-1.73526	4.99556	0.94082
H	-1.12346	5.90202	0.99855
C	-1.82525	4.12527	2.03674
H	-1.27524	4.36178	2.95595
C	-2.61883	2.95856	1.99537
C	-4.06747	3.30147	-1.59369
H	-4.74582	2.45768	-1.37783
C	-4.92514	4.51702	-2.00708
H	-4.30316	5.38445	-2.29234
H	-5.55321	4.26400	-2.87910
H	-5.59040	4.83930	-1.18849
C	-3.14106	2.86736	-2.75201
H	-2.58946	1.94424	-2.49282
H	-3.72251	2.66818	-3.66977
H	-2.40139	3.65498	-2.98792
C	-2.66228	2.06675	3.23738
H	-3.31142	1.21098	2.98936
C	-3.26836	2.80306	4.45376
H	-4.27865	3.18708	4.23589
H	-3.34155	2.12276	5.32073
H	-2.64436	3.66240	4.75855
C	-1.27151	1.49804	3.59350
H	-1.33322	0.84760	4.48370
H	-0.87152	0.88692	2.76483
H	-0.54451	2.29941	3.82026
C	-7.08878	0.76118	0.13354
H	-7.02235	1.32772	-0.81476
H	-8.05809	1.06267	0.58347
C	-7.08918	-0.76051	-0.13412
H	-7.02331	-1.32717	0.81415

H	-8.05856	-1.06140	-0.58432
C	7.08908	-0.76111	0.13384
H	7.02320	-1.32778	-0.81442
H	8.05841	-1.06207	0.58410
C	7.08883	0.76055	-0.13393
H	7.02239	1.32723	0.81429
H	8.05820	1.06193	-0.58383

Method C & D

SCF (BP86/BS4) Energy = -5406.39651910

Enthalpy 0K = -5404.974491

Enthalpy 298K = -5404.877017

Free Energy 298K = -5405.112147

Lowest Frequency = 12.7120 cm^{-1}

Second Frequency = 13.2143 cm^{-1}

SCF (BP86-D3^{BJ}) Energy = -5406.887020

SCF (PCM=C_6H_6) Energy = -5406.401534

SCF (SMD=C_6H_6) Energy = -5406.426905

SCF (BS5) Energy = -5409.919190

K	-0.00002	2.20256	0.00007
Si	5.76648	1.44971	-1.33824
Si	5.76683	-1.44967	1.33855
Al	3.00077	0.00003	0.00046
N	4.11136	1.44051	-0.66862
N	4.11183	-1.44041	0.66865
C	3.34091	2.63710	-0.77331
C	3.25000	3.53826	0.34704
C	2.43089	4.68463	0.26402
H	2.37141	5.36711	1.12786
C	1.70653	4.98803	-0.89950
H	1.08659	5.89672	-0.95255
C	1.79860	4.12308	-2.00043
H	1.24070	4.36362	-2.92117
C	2.59451	2.95604	-1.96380
C	4.02818	3.27414	1.63682
H	4.75068	2.46672	1.39568
C	4.82768	4.50030	2.12004
H	5.48981	4.89624	1.32252
H	5.46472	4.22940	2.98833
H	4.16638	5.33075	2.44873
C	3.10164	2.74110	2.75002
H	2.31470	3.48265	3.01071
H	3.67297	2.51765	3.67625
H	2.59348	1.80323	2.43047
C	2.63512	2.06644	-3.20747
H	3.30299	1.21697	-2.96201
C	3.22114	2.80329	-4.43027
H	2.57452	3.64881	-4.74972
H	3.31440	2.11358	-5.29603
H	4.22741	3.21763	-4.21630
C	1.25189	1.47115	-3.53781
H	0.87455	0.85605	-2.69276
H	1.30847	0.80791	-4.42677
H	0.49999	2.25899	-3.76174
C	5.91702	0.41309	-2.93300
H	6.98707	0.27733	-3.20212
H	5.41548	0.89630	-3.79637
H	5.47175	-0.59607	-2.80759
C	6.29186	3.23472	-1.72974
H	6.35808	3.84850	-0.80692
H	5.58300	3.74424	-2.41415
H	7.29299	3.23975	-2.21179
C	5.91709	-0.41308	2.93336
H	6.98710	-0.27693	3.20241
H	5.41582	-0.89653	3.79677
H	5.47140	0.59591	2.80804
C	6.29239	-3.23463	1.72987
H	6.35921	-3.84808	0.80686
H	5.58340	-3.74460	2.41381
H	7.29333	-3.23951	2.21233
C	3.34141	-2.63709	0.77299
C	2.59510	-2.95651	1.96341
C	1.79924	-4.12359	1.99966
H	1.24146	-4.36451	2.92037
C	1.70713	-4.98812	0.89840
H	1.08726	-5.89687	0.95114
C	2.43135	-4.68422	-0.26506
H	2.37180	-5.36635	-1.12918
C	3.25043	-3.53778	-0.34770
C	2.63578	-2.06743	3.20748
H	3.30370	-1.21789	2.96240
C	3.22170	-2.80480	4.42998
H	2.57510	-3.65046	4.74907
H	3.31491	-2.11543	5.29603
H	4.22800	-3.21903	4.21590
C	1.25255	-1.47216	3.53795
H	0.87544	-0.85663	2.69312
H	1.30909	-0.80933	4.42723
H	0.50056	-2.26004	3.76143
C	4.02844	-3.27311	-1.63747
H	4.75063	-2.46543	-1.39618
C	4.82844	-4.49885	-2.12089
H	5.49080	-4.89457	-1.32346
H	5.46530	-4.22758	-2.98920
H	4.16747	-5.32957	-2.44958
C	3.10163	-2.74020	-2.75050
H	3.67284	-2.51629	-3.67670
H	2.59314	-1.80260	-2.43071
H	2.31499	-3.48201	-3.01134
K	0.00017	-2.20224	-0.00018
Si	-5.76677	-1.44964	-1.33839
Si	-5.76656	1.44979	1.33830
Al	-3.00077	-0.00013	0.00010
N	-4.11176	-1.44054	-0.66849
N	-4.11144	1.44048	0.66860
C	-3.34136	-2.63720	-0.77289
C	-2.59507	-2.95665	-1.96332

```
C  -1.79917 -4.12371 -1.99954
H  -1.24139 -4.36464 -2.92024
C  -1.70697 -4.98819 -0.89825
H  -1.08705 -5.89690 -0.95098
C  -2.43119 -4.68428  0.26522
H  -2.37162 -5.36639  1.12935
C  -3.25031 -3.53789  0.34783
C  -2.63575 -2.06758 -3.20738
H  -3.30356 -1.21798 -2.96226
C  -3.22183 -2.80493 -4.42984
H  -4.22811 -3.21916 -4.21568
H  -3.31510 -2.11557 -5.29588
H  -2.57525 -3.65060 -4.74896
C  -1.25252 -1.47246 -3.53804
H  -0.50062 -2.26040 -3.76160
H  -1.30912 -0.80966 -4.42734
H  -0.87521 -0.85691 -2.69331
C  -4.02840 -3.27329  1.63756
H  -4.75071 -2.46575  1.39623
C  -4.82820 -4.49916  2.12100
H  -4.16709 -5.32977  2.44970
H  -5.46510 -4.22799  2.98931
H  -5.49051 -4.89501  1.32358
C  -3.10173 -2.74024  2.75065
H  -2.59337 -1.80255  2.43090
H  -3.67302 -2.51648  3.67683
H  -2.31496 -3.48191  3.01149
C  -6.29233 -3.23464 -1.72959
H  -7.29352 -3.23971 -2.21152
H  -5.58359 -3.74434 -2.41400
H  -6.35845 -3.84825 -0.80665
C  -5.91703 -0.41319 -2.93330
H  -5.47131  0.59581 -2.80808
H  -5.41573 -0.89673 -3.79664
H  -6.98703 -0.27703 -3.20240
C  -6.29193  3.23474  1.72988
H  -7.29289  3.23966  2.21231
H  -5.58290  3.74454  2.41391
H  -6.35867  3.84834  0.80696
C  -5.91698  0.41293  2.93291
H  -5.47113 -0.59597  2.80748
H  -5.41578  0.89635  3.79638
H  -6.98697  0.27659  3.20193
C  -3.34102  2.63711  0.77317
C  -3.24999  3.53801 -0.34738
C  -2.43089  4.68440 -0.26453
H  -2.37129  5.36667 -1.12854
C  -1.70670  4.98810  0.89901
H  -1.08679  5.89682  0.95193
C  -1.79890  4.12341  2.00014
H  -1.24113  4.36417  2.92090
C  -2.59477  2.95634  1.96367
C  -4.02801  3.27358 -1.63719
H  -4.75041  2.46608 -1.39597
C  -4.82765  4.49955 -2.12063
H  -4.16643  5.33007 -2.44934
H  -5.46458  4.22846 -2.98894
H  -5.48991  4.89550 -1.32321
C  -3.10133  2.74048 -2.75025
H  -2.59313  1.80270 -2.43054
H  -3.67259  2.51686 -3.67648
H  -2.31445  3.48208 -3.01099
C  -2.63544  2.06700  3.20754
H  -3.30330  1.21748  2.96225
C  -3.22147  2.80408  4.43019
H  -4.22775  3.21836  4.21616
H  -3.31472  2.11450  5.29606
H  -2.57487  3.64966  4.74951
C  -1.25220  1.47178  3.53798
H  -1.30876  0.80874  4.42711
H  -0.87493  0.85646  2.69307
H  -0.50030  2.25967  3.76170
C  -7.07043  0.76018  0.13260
H  -7.01558  1.33097 -0.82209
H  -8.04099  1.06505  0.59251
C  -7.07068 -0.75949 -0.13304
H  -7.01632 -1.33029  0.82168
H  -8.04123 -1.06396 -0.59323
C   7.07074 -0.75963  0.13314
H   7.01641 -1.33055 -0.82151
H   8.04132 -1.06399  0.59335
C   7.07045  0.76001 -0.13268
H   7.01585  1.33088  0.82198
H   8.04095  1.06474 -0.59283
```

K(0)

Method A & B

SCF (BP86/BS1) Energy = -28.3228762473

Enthalpy 0K = -28.322876

Enthalpy 298K = -28.320516

Free Energy 298K = -28.338707

SCF (BP86-D3^{BJ}) Energy = -28.32287625

SCF (C_6H_6) Energy = -28.3255799

SCF (BS2) Energy = -599.9635241

SCF (BP86-D3^{BJ}(C_6H_6)/BS3) Energy = -603.63729946

```
K   0.00000  0.00000  0.00000
```

Method C & D

SCF (BP86/BS4) Energy = -599.883546653

Enthalpy 0K = -599.883547

Enthalpy 298K = -599.881186

Free Energy 298K = -599.899377

SCF (BP86-D3^{BJ}) Energy = -599.883546653

SCF (PCM=C_6H_6) Energy = -599.886566

SCF (SMD=C_6H_6) Energy =

-599.881959
SCF (BS5) Energy = -599.961730916

K 0.00000 0.00000 0.00000

4^Rb^

Method A & B
SCF (BP86/BS1) Energy = -2633.94560140
Enthalpy 0K = -2632.515733
Enthalpy 298K = -2632.417698
Free Energy 298K = -2632.655717
Lowest Frequency = 12.7495 cm^{-1}
Second Frequency = 14.5842 cm^{-1}
SCF (BP86-D3^{BJ}) Energy = -2634.42967725
SCF (C_6H_6) Energy = -2633.95114511
SCF (BS2) Energy = -4257.93276718
SCF (BP86-D3^{BJ}(C_6H_6)/BS3) Energy = -10226.23565952

Rb	-0.00042	-2.31861	0.00034
H	-0.96865	-0.88543	-2.76807
H	0.66518	-2.28089	3.85553
H	0.96848	-0.88622	2.76843
Rb	0.00010	2.31798	-0.00111
H	-0.96825	0.88530	2.76749
Si	-5.90060	-1.40864	-1.38571
Si	-5.90055	1.40840	1.38613
Si	5.90041	1.40898	-1.38567
Si	5.90072	-1.40844	1.38560
Al	-3.11420	0.00015	-0.00018
Al	3.11413	-0.00001	0.00011
N	-4.23823	-1.42957	-0.71511
N	-4.23831	1.42950	0.71524
N	4.23820	1.42985	-0.71465
N	4.23842	-1.42959	0.71478
C	-3.49075	-2.64064	-0.85468
C	-3.41790	-3.57173	0.23882
C	-2.62637	-4.73277	0.12359
H	-2.58697	-5.43675	0.96355
C	-1.91563	-5.02409	-1.04994
H	-1.32754	-5.94473	-1.13178
C	-1.99129	-4.12783	-2.12567
H	-1.45199	-4.35657	-3.05332
C	-2.75649	-2.94385	-2.05413
C	-4.19439	-3.33153	1.53399
H	-4.86329	-2.47598	1.33563
C	-3.25285	-2.92458	2.68998
H	-2.51917	-3.72338	2.90696
H	-3.82306	-2.73352	3.61641
H	-2.69573	-2.00230	2.43893
C	-5.06465	-4.54096	1.93841
H	-5.74043	-4.84354	1.12090
H	-5.68263	-4.29100	2.81849
H	-4.45250	-5.42040	2.20776
C	-2.77959	-2.02095	-3.27411
H	-3.41336	-1.15950	-3.00652
C	-1.37674	-1.46988	-3.61194
H	-1.42270	-0.79831	-4.48726
H	-0.66462	-2.27985	-3.85530
C	-3.39418	-2.71400	-4.51138
H	-2.78590	-3.57734	-4.83595
H	-3.45047	-2.01056	-5.36102
H	-4.41265	-3.08361	-4.30733
C	-6.44248	-3.18052	-1.84414
H	-5.72084	-3.68339	-2.50803
H	-7.41846	-3.15339	-2.35986
H	-6.55564	-3.80821	-0.94384
C	-6.03852	-0.30812	-2.94470
H	-5.57642	0.68040	-2.78170
H	-7.10001	-0.14361	-3.20338
H	-5.54938	-0.76754	-3.81933
C	-7.20506	-0.75771	-0.15046
H	-8.17351	-1.04870	-0.60909
H	-7.14076	-1.34524	0.78516
C	-7.20509	0.75741	0.15098
H	-8.17355	1.04837	0.60961
H	-7.14082	1.34493	-0.78465
C	-6.03823	0.30789	2.94512
H	-5.57668	-0.68085	2.78190
H	-7.09969	0.14388	3.20426
H	-5.54850	0.76705	3.81956
C	-6.44250	3.18023	1.84470
H	-5.72105	3.68294	2.50893
H	-7.41865	3.15303	2.36010
H	-6.55534	3.80809	0.94449
C	-3.49090	2.64065	0.85479
C	-2.75633	2.94373	2.05408
C	-1.99114	4.12770	2.12555
H	-1.45152	4.35631	3.05305
C	-1.91579	5.02409	1.04991
H	-1.32768	5.94472	1.13171
C	-2.62687	4.73292	-0.12345
H	-2.58772	5.43700	-0.96333
C	-3.41839	3.57187	-0.23860
C	-2.77913	2.02071	3.27399
H	-3.41291	1.15924	3.00645
C	-1.37618	1.46968	3.61148
H	-1.42192	0.79808	4.48679
H	-0.66404	2.27968	3.85472
C	-3.39351	2.71362	4.51143
H	-3.44959	2.01011	5.36103
H	-4.41203	3.08321	4.30762
H	-2.78520	3.57697	4.83595
C	-4.19517	3.33178	-1.53363
H	-4.86405	2.47623	-1.33520
C	-5.06550	4.54125	-1.93779
H	-4.45339	5.42068	-2.20726
H	-5.74107	4.84383	-1.12011
H	-5.68370	4.29134	-2.81773

C -3.25389 2.92490 -2.68985
H -2.52025 3.72371 -2.90692
H -3.82430 2.73391 -3.61617
H -2.69670 2.00261 -2.43897
C 3.49064 2.64087 -0.85428
C 2.75647 2.94410 -2.05379
C 1.99121 4.12804 -2.12534
H 1.45196 4.35677 -3.05303
C 1.91536 5.02422 -1.04957
H 1.32722 5.94483 -1.13143
C 2.62602 4.73289 0.12401
H 2.58651 5.43683 0.96399
C 3.41763 3.57191 0.23925
C 2.77970 2.02127 -3.27384
H 3.41354 1.15985 -3.00630
C 1.37692 1.47006 -3.61179
C 3.39425 2.71445 -4.51106
H 2.78585 3.57772 -4.83562
H 3.45067 2.01105 -5.36072
H 4.41264 3.08421 -4.30695
C 4.19409 3.33173 1.53445
H 4.86299 2.47618 1.33609
C 3.25253 2.92478 2.69042
H 3.82274 2.73366 3.61684
H 2.69535 2.00255 2.43933
H 2.51889 3.72360 2.90742
C 5.06435 4.54116 1.93886
H 4.45221 5.42063 2.20811
H 5.74019 4.84367 1.12138
H 5.68226 4.29123 2.81900
C 6.03827 0.30866 -2.94480
H 5.57612 -0.67987 -2.78200
H 7.09978 0.14414 -3.20341
H 5.54925 0.76821 -3.81942
C 6.44218 3.18093 -1.84402
H 5.72042 3.68384 -2.50777
H 7.41808 3.15386 -2.35988
H 6.55545 3.80854 -0.94368
C 7.20505 0.75794 -0.15067
H 8.17346 1.04906 -0.60931
H 7.14078 1.34527 0.78507
C 7.20513 -0.75724 0.15044
H 8.17363 -1.04832 0.60891
H 7.14074 -1.34456 -0.78530
C 6.44284 -3.18031 1.84381
H 5.72099 -3.68364 2.50713
H 7.41855 -3.15311 2.36003
H 6.55664 -3.80761 0.94332
C 6.03841 -0.30796 2.94462
H 5.57649 0.68062 2.78147
H 7.09988 -0.14357 3.20348
H 5.54905 -0.76724 3.81919
C 3.49108 -2.64075 0.85438
C 3.41828 -3.57180 -0.23913
C 2.62692 -4.73296 -0.12388
H 2.58755 -5.43691 -0.96385
C 1.91632 -5.02441 1.04970
H 1.32845 -5.94519 1.13161
C 1.99192 -4.12817 2.12544
H 1.45271 -4.35699 3.05313
C 2.75691 -2.94406 2.05386
C 4.19464 -3.33145 -1.53435
H 4.86341 -2.47579 -1.33601
C 3.25295 -2.92461 -2.69026
H 2.51942 -3.72354 -2.90725
H 3.82307 -2.73336 -3.61670
H 2.69564 -2.00247 -2.43909
C 5.06508 -4.54071 -1.93887
H 5.74101 -4.84317 -1.12144
H 5.68291 -4.29064 -2.81902
H 4.45307 -5.42027 -2.20815
C 2.77995 -2.02121 3.27390
H 3.41335 -1.15951 3.00622
C 1.37698 -1.47066 3.61210
H 1.42291 -0.79919 4.48751
C 3.39506 -2.71412 4.51099
H 3.45126 -2.01073 5.36067
H 4.41360 -3.08336 4.30670
H 2.78714 -3.57770 4.83562
H 0.66467 2.27998 -3.85494
H 0.96893 0.88528 -2.76810
H 1.42299 0.79873 -4.48729

Method C & D
SCF (BP86/BS4) Energy =
-4254.86019710
Enthalpy 0K = -4253.438668
Enthalpy 298K = -4253.340810
Free Energy 298K = -4253.578816
Lowest Frequency = 12.3406 cm^{-1}
Second Frequency = 14.1341 cm^{-1}
SCF (BP86-$D3^{BJ}$) Energy =
-4255.34739705
SCF (PCM=C_6H_6) Energy =
-4254.865453
SCF (SMD=C_6H_6) Energy =
-4254.899222
SCF (BS5) Energy = -4258.22964408

Rb 0.00015 -2.25381 -0.00189
H 1.02023 -0.81240 2.71754
H -0.66565 -2.18391 -3.83881
H -1.02222 -0.81397 -2.71906
Rb 0.00016 2.25858 0.00011
H 1.02176 0.81205 -2.71672
Si 5.91735 -1.39455 1.39238
Si 5.91904 1.39252 -1.39029
Si -5.91832 1.39389 1.39051
Si -5.91785 -1.39475 -1.39062
Al 3.15123 0.00022 0.00076
Al -3.15101 0.00030 -0.00054
N 4.26392 -1.41911 0.71653

N	4.26526	1.41827	-0.71530
N	-4.26474	1.41898	0.71496
N	-4.26410	-1.41917	-0.71552
C	3.50918	-2.62261	0.85967
C	3.43807	-3.56542	-0.22726
C	2.64383	-4.72564	-0.10273
H	2.60353	-5.44268	-0.93964
C	1.92610	-5.00349	1.07162
H	1.33031	-5.92628	1.15922
C	2.00005	-4.09728	2.14094
H	1.45221	-4.31958	3.07256
C	2.76955	-2.91493	2.06157
C	4.21700	-3.33597	-1.52360
H	4.91112	-2.49527	-1.31528
C	3.28615	-2.88610	-2.66980
H	2.52752	-3.66497	-2.90520
H	3.86171	-2.68828	-3.59931
H	2.74647	-1.95036	-2.40095
C	5.05994	-4.55690	-1.94263
H	5.72851	-4.89205	-1.12297
H	5.69447	-4.30732	-2.81914
H	4.42839	-5.42363	-2.23433
C	2.79517	-1.98213	3.27433
H	3.45208	-1.13282	3.00037
C	1.40438	-1.39235	3.58406
H	1.45415	-0.69729	4.44907
H	0.66303	-2.18141	3.83817
C	3.38784	-2.66653	4.52414
H	2.75196	-3.50854	4.87333
H	3.46986	-1.94459	5.36437
H	4.40001	-3.07483	4.32758
C	6.44823	-3.15918	1.86180
H	5.73879	-3.64393	2.56327
H	7.44717	-3.13881	2.34791
H	6.52181	-3.81012	0.96546
C	6.05632	-0.28938	2.94131
H	5.60933	0.71195	2.76835
H	7.12431	-0.13889	3.21068
H	5.55035	-0.73550	3.82188
C	7.22679	-0.75481	0.16534
H	8.19552	-1.04013	0.64136
H	7.17593	-1.36408	-0.76552
C	7.22722	0.75247	-0.16210
H	8.19652	1.03745	-0.63714
H	7.17562	1.36177	0.76871
C	6.05838	0.28684	-2.93879
H	5.61124	-0.71440	-2.76566
H	7.12646	0.13616	-3.20770
H	5.55279	0.73272	-3.81970
C	6.45111	3.15668	-1.86013
H	5.74245	3.64141	-2.56240
H	7.45042	3.13559	-2.34546
H	6.52432	3.80804	-0.96407
C	3.51127	2.62207	-0.85968
C	2.77232	2.91384	-2.06214
C	2.00376	4.09671	-2.14287
H	1.45655	4.31865	-3.07495
C	1.93000	5.00387	-1.07435
H	1.33502	5.92709	-1.16304
C	2.64691	4.72645	0.10061
H	2.60671	5.44420	0.93692
C	3.44027	3.56576	0.22648
C	2.79772	1.97995	-3.27405
H	3.45394	1.13043	-2.99909
C	1.40660	1.39088	-3.58369
H	1.45613	0.69497	-4.44803
H	0.66591	2.18025	-3.83880
C	3.39130	2.66280	-4.52426
H	3.47315	1.94004	-5.36381
H	4.40367	3.07061	-4.32770
H	2.75612	3.50493	-4.87447
C	4.21833	3.33674	1.52342
H	4.91214	2.49555	1.31605
C	5.06164	4.55749	1.94220
H	4.43038	5.42474	2.23293
H	5.73093	4.89175	1.12276
H	5.69547	4.30813	2.81928
C	3.28656	2.88805	2.66933
H	2.52829	3.66753	2.90390
H	3.86148	2.69036	3.59926
H	2.74644	1.95250	2.40067
C	-3.51063	2.62280	0.85854
C	-2.77189	2.91551	2.06089
C	-2.00344	4.09850	2.14090
H	-1.45646	4.32119	3.07294
C	-1.92955	5.00490	1.07174
H	-1.33475	5.92828	1.15991
C	-2.64619	4.72654	-0.10316
H	-2.60595	5.44371	-0.93996
C	-3.43946	3.56572	-0.22831
C	-2.79736	1.98250	3.27349
H	-3.45344	1.13270	2.99908
C	-1.40621	1.39382	3.58375
C	-3.39113	2.66620	4.52315
H	-2.75605	3.50863	4.87280
H	-3.47299	1.94404	5.36321
H	-4.40352	3.07377	4.32621
C	-4.21741	3.33577	-1.52515
H	-4.91117	2.49467	-1.31724
C	-3.28556	2.88640	-2.67074
H	-3.86043	2.68813	-3.60058
H	-2.74542	1.95105	-2.40148
H	-2.52731	3.66576	-2.90575
C	-5.06080	4.55619	-1.94478
H	-4.42958	5.42328	-2.23610
H	-5.73012	4.89095	-1.12558
H	-5.69459	4.30619	-2.82170
C	-6.05746	0.28932	2.93985
H	-5.60995	-0.71189	2.76754
H	-7.12553	0.13846	3.20868
H	-5.55219	0.73599	3.82054
C	-6.45012	3.15849	1.85903

H -5.74104 3.64391 2.56040
H -7.44915 3.13791 2.34493
H -6.52380 3.80896 0.96234
C -7.22700 0.75304 0.16328
H -8.19606 1.03826 0.63867
H -7.17591 1.36177 -0.76792
C -7.22665 -0.75443 -0.16323
H -8.19563 -1.04014 -0.63850
H -7.17516 -1.36315 0.76796
C -6.44917 -3.15954 -1.85895
H -5.74008 -3.64488 -2.56034
H -7.44827 -3.13922 -2.34473
H -6.52263 -3.80991 -0.96218
C -6.05752 -0.29017 -2.93992
H -5.61023 0.71115 -2.76758
H -7.12564 -0.13955 -3.20869
H -5.55218 -0.73667 -3.82066
C -3.50972 -2.62291 -0.85851
C -3.43791 -3.56508 0.22895
C -2.64423 -4.72569 0.10436
H -2.60349 -5.44225 0.94165
C -1.92783 -5.00458 -1.07055
H -1.33269 -5.92779 -1.15826
C -2.00243 -4.09899 -2.14033
H -1.45572 -4.32216 -3.07242
C -2.77130 -2.91623 -2.06091
C -4.21572 -3.33468 1.52578
H -4.90968 -2.49382 1.31758
C -3.28386 -2.88464 2.67109
H -2.52535 -3.66370 2.90629
H -3.85867 -2.68618 3.60092
H -2.74399 -1.94924 2.40142
C -5.05883 -4.55508 1.94603
H -5.72817 -4.89033 1.12704
H -5.69259 -4.30483 2.82290
H -4.42741 -5.42192 2.23764
C -2.79752 -1.98405 -3.27414
H -3.45413 -1.13450 -3.00021
C -1.40682 -1.39458 -3.58493
H -1.45691 -0.70015 -4.45043
C -3.39094 -2.66892 -4.52332
H -3.47344 -1.94731 -5.36379
H -4.40301 -3.07711 -4.32601
H -2.75529 -3.51108 -4.87257
H -0.66567 2.18347 3.83845
H -1.02111 0.81441 2.71730
H -1.45579 0.69851 4.44857

Rb(0)
Method A & B
SCF (BP86/BS1) Energy = -24.1221090118
Enthalpy 0K = -24.122109
Enthalpy 298K = -24.119749
Free Energy 298K = -24.139043
SCF (BP86-D3^{BJ}) Energy = -24.12210901
SCF (C_6H_6) Energy = -24.12422605
SCF (BS2) Energy = -24.12210901
SCF (BP86-D3^{BJ}(C_6H_6)/BS3) Energy = -3004.32186991

Rb 0.00000 0.00000 0.00000

Method C & D
SCF (BP86/BS4) Energy = -24.1218579598
Enthalpy 0K = -24.121858
Enthalpy 298K = -24.119497
Free Energy 298K = -24.138792
SCF (BP86-D3^{BJ}) Energy = -24.1218579598
SCF (PCM=C_6H_6) Energy = -24.124000
SCF (SMD=C_6H_6) Energy = -24.124613
SCF (BS5) Energy = -24.1222139551

Rb 0.00000 0.00000 0.00000

4^{Cs}
Method A & B
SCF (BP86/BS1) Energy = -2626.082908
Enthalpy 0K = -2624.653689
Enthalpy 298K = -2624.555432
Free Energy 298K = -2624.795269
SCF (BP86-D3^{BJ}) Energy = -2626.565825
Lowest Frequency = 12.0088 cm^{-1}
Second Frequency = 12.1241 cm^{-1}
SCF (C_6H_6) Energy = -2626.088137
SCF (BS2) Energy = -4250.070789
SCF (BP86-D3^{BJ}(C_6H_6)/BS3) Energy = -20050.40162704

Cs 0.000775 -2.435704 -0.000686
Cs 0.000311 2.437230 -0.002853
Al 3.219723 0.000435 -0.000569
Si -6.013557 -1.336859 1.447508
Si 6.013238 1.335413 1.448395
Al -3.219737 -0.000396 0.000212
Si 6.014721 -1.335460 -1.445722
Si -6.014801 1.335632 -1.445188
N 4.356317 -1.398403 -0.767573
N 4.355612 1.398726 0.768351
N -4.355668 -1.399259 0.768024
N -4.356363 1.398345 -0.767050
C 3.623317 -2.609589 -0.967220
C 3.622585 2.609994 0.967446
C 2.887332 2.860443 2.178370
C 2.889099 -2.860023 -2.178775
C 2.149264 -4.053939 -2.315425
H 1.608438 -4.239456 -3.251848

C 3.572985 -3.602000 0.072388
C 3.573364 3.602488 -0.072108
C -3.622505 -2.610595 0.966234
C -3.623682 2.609816 -0.966062
C 2.147496 4.054418 2.314442
H 1.605873 4.239958 3.250397
C 2.884669 -1.866918 -3.342450
H 3.525552 -1.022693 -3.039313
C -3.573535 3.601722 0.074051
C 2.808351 -4.772754 -0.106808
H 2.787270 -5.523849 0.692053
C 2.099450 -5.012754 -1.293338
H 1.531984 -5.940765 -1.426101
C -2.889742 2.861146 -2.177605
C -2.887677 -2.862012 2.177208
C 4.341449 -3.411573 1.380363
H 5.007971 -2.546310 1.219648
C 2.808706 4.773310 0.106504
H 2.788552 5.524527 -0.692263
C -2.807810 -4.772939 0.103557
H -2.787056 -5.523335 -0.695972
C -2.098338 -5.013930 1.289539
H -1.530746 -5.942021 1.421215
C 2.881586 1.867135 3.341846
H 3.522822 1.022968 3.039312
C -4.341743 3.410408 1.382045
H -5.007842 2.544861 1.221090
C 4.343111 3.412160 -1.379341
H 5.009462 2.546873 -1.218041
C 6.556397 3.076937 2.010657
H 6.667606 3.758488 1.150148
H 7.533348 3.018087 2.521898
H 5.835999 3.538138 2.705535
C -3.572640 -3.602101 -0.074254
C -2.147734 -4.056014 2.312493
H -1.606399 -4.242280 3.248474
C 2.098726 5.013308 1.292373
H 1.531222 5.941365 1.424675
C 7.327303 -0.748977 -0.186799
H 7.268172 -1.382698 0.718548
H 8.293512 -1.015362 -0.664679
C -6.558393 3.077521 -2.005896
H -6.668668 3.758589 -1.144889
H -7.535853 3.019073 -2.516204
H -5.838626 3.539050 -2.701215
C 1.474381 -1.295488 -3.606858
H 0.751309 -2.090821 -3.866727
H 1.494665 -0.580616 -4.448440
H 1.099847 -0.752996 -2.720074
C -2.150487 4.055468 -2.313843
H -1.609865 4.241646 -3.250254
C 5.213155 -4.631376 1.747089
H 4.603344 -5.521891 1.983667
H 5.825920 -4.409622 2.638280
H 5.894152 -4.903255 0.923139
C -2.809422 4.772875 -0.104723
H -2.788516 5.523597 0.694494
C -7.327214 -0.749626 0.190077
H -7.268874 -1.382824 -0.715690
H -8.292973 -1.016357 0.668680
C 6.139040 -0.151869 -2.943990
H 5.645617 -0.565915 -3.838731
H 7.198086 0.030920 -3.200438
H 5.673993 0.824085 -2.724166
C 7.327091 0.748831 0.190835
H 7.268886 1.382553 -0.714575
H 8.292805 1.015242 0.669703
C -2.882512 -1.869751 3.341593
H -3.523479 -1.025245 3.039429
C -2.884878 1.868452 -3.341634
H -3.525405 1.023857 -3.038784
C 6.558163 -3.077097 -2.007332
H 6.668083 -3.758697 -1.146699
H 7.535772 -3.018507 -2.517333
H 5.838516 -3.538094 -2.703127
C -7.327430 0.748388 -0.186688
H -7.268422 1.381614 0.719015
H -8.293591 1.015026 -0.664533
C 6.135926 0.151814 2.946795
H 5.642168 0.566137 3.841220
H 7.194752 -0.031395 3.203882
H 5.670621 -0.823964 2.726761
C 1.470940 1.295683 3.604213
H 1.490128 0.579954 4.445087
H 1.097336 0.754230 2.716403
H 0.747731 2.090909 3.864024
C -6.136766 -0.154305 2.946698
H -5.643405 -0.569349 3.841010
H -7.195646 0.028935 3.203532
H -5.671176 0.821535 2.727527
C -2.100904 5.013803 -1.291295
H -1.533885 5.942142 -1.423695
C -6.556557 -3.078901 2.008320
H -6.668311 -3.759564 1.147178
H -7.533210 -3.020492 2.520167
H -5.835795 -3.540880 2.702305
C 3.388435 -3.049803 2.542057
H 2.836288 -2.116331 2.322342
H 3.948881 -2.896826 3.481574
H 2.651643 -3.855958 2.718581
C -4.341906 -3.410769 -1.381616
H -5.008004 -2.545342 -1.220051
C 3.467700 -2.482274 -4.634455
H 4.488969 -2.866965 -4.476891
H 3.508266 -1.726266 -5.438595
H 2.849067 -3.321369 -5.000671
C -6.138935 0.152870 -2.944127
H -5.645266 0.567378 -3.838517
H -7.197929 -0.029699 -3.200941
H -5.674005 -0.823241 -2.724750
C -1.471989 -1.298691 3.605463
H -1.494254 0.582799 -4.447659

H -1.099501 0.755453 -2.719314
H -0.751669 2.093419 -3.866045
C -3.389598 -3.048897 -2.543856
H -2.836879 -2.115776 -2.324116
H -3.950696 -2.895266 -3.482876
H -2.653277 -3.855282 -2.721287
C -3.464803 -2.485919 4.633536
H -2.846130 -3.325445 4.998697
H -4.486279 -2.870229 4.476394
H -3.504619 -1.730499 5.438267
C -5.214057 4.629625 1.749293
H -5.826613 4.407221 2.640465
H -5.895281 4.901456 0.925514
H -4.604684 5.520369 1.986139
C -3.468100 2.483966 -4.633471
H -2.849840 3.323478 -4.999366
H -4.489559 2.868123 -4.475838
H -3.508260 1.728212 -5.437871

Method C & D

SCF (BP86/BS4) Energy = -4246.99539461
Enthalpy 0K = -4245.574231
Enthalpy 298K = -4245.476345
Free Energy 298K = -4245.714899
Lowest Frequency = 11.4752 cm^{-1}
Second Frequency = 12.9146 cm^{-1}
SCF (BP86-D3^{BJ}) Energy = -4247.47997077
SCF (PCM=C_6H_6) Energy = -4247.000236
SCF (SMD=C_6H_6) Energy = -4247.032790
SCF (BS5) Energy = -4250.36743475

Cs -0.00035 -2.39558 -0.00082
Cs 0.00048 2.39753 -0.00094
Al 3.27326 -0.00017 0.00027
Si -6.04708 -1.31069 1.46091
Si 6.04783 1.30988 1.46014
Al -3.27327 0.00010 0.00024
Si 6.04732 -1.31014 -1.46069
Si -6.04813 1.30937 -1.45978
N 4.39903 -1.38472 -0.77521
N 4.39942 1.38454 0.77497
N -4.39879 -1.38488 0.77536
N -4.39977 1.38444 -0.77455
C 3.66297 -2.59061 -0.98050
C 3.66330 2.59030 0.98077
C 2.92319 2.82830 2.19419
C 2.92308 -2.82922 -2.19398
C 2.18499 -4.02462 -2.34238
H 1.63695 -4.20337 -3.28323
C 3.62040 -3.59854 0.04801
C 3.62100 3.59879 -0.04724
C -3.66241 -2.59051 0.98090
C -3.66387 2.59028 -0.98051
C 2.18524 4.02371 2.34312
H 1.63708 4.20202 3.28397
C 2.91410 -1.82102 -3.34514
H 3.58906 -0.99628 -3.04196
C -3.62161 3.59886 0.04732
C 2.85881 -4.77127 -0.14452
H 2.84281 -5.53877 0.64724
C 2.14323 -4.99722 -1.33131
H 1.57393 -5.93015 -1.47341
C -2.92383 2.82825 -2.19403
C -2.92217 -2.82854 2.19425
C 4.39376 -3.42167 1.35568
H 5.08107 -2.56654 1.18708
C 2.85960 4.77157 0.14582
H 2.84390 5.53953 -0.64549
C -2.85817 -4.77139 0.14555
H -2.84227 -5.53919 -0.64592
C -2.14226 -4.99678 1.33224
H -1.57279 -5.92957 1.47455
C 2.91411 1.81968 3.34498
H 3.58771 0.99417 3.04078
C -4.39498 3.42248 1.35505
H -5.08269 2.56765 1.18657
C 4.39436 3.42234 -1.35497
H 5.08206 2.56751 -1.18646
C 6.58314 3.03735 2.04835
H 6.65996 3.74936 1.20000
H 7.58129 2.97961 2.53318
H 5.87421 3.47385 2.78134
C -3.61992 -3.59882 -0.04724
C -2.18386 -4.02379 2.34292
H -1.63555 -4.20211 3.28369
C 2.14386 4.99693 1.33263
H 1.57478 5.92991 1.47521
C 7.36637 -0.74299 -0.20808
H 7.32208 -1.40551 0.68605
H 8.33240 -0.99825 -0.70611
C -6.58379 3.03670 -2.04810
H -6.66031 3.74886 -1.19985
H -7.58213 2.97880 -2.53253
H -5.87518 3.47311 -2.78146
C 1.51845 -1.19938 -3.55520
H 0.75459 -1.96695 -3.80961
H 1.53389 -0.46205 -4.38596
H 1.19117 -0.66031 -2.63962
C -2.18592 4.02369 -2.34305
H -1.63768 4.20192 -3.28388
C 5.24473 -4.65185 1.72727
H 4.61977 -5.53466 1.98286
H 5.87539 -4.43282 2.61465
H 5.91785 -4.94836 0.89651
C -2.86028 4.77163 -0.14581
H -2.84460 5.53966 0.64543
C -7.36630 -0.74364 0.20847
H -7.32207 -1.40616 -0.68567

H	-8.33224	-0.99905	0.70663
C	6.16842	-0.11064	-2.93956
H	5.65628	-0.50464	-3.84135
H	7.23324	0.06136	-3.20866
H	5.71853	0.87556	-2.70083
C	7.36659	0.74230	0.20744
H	7.32247	1.40484	-0.68668
H	8.33271	0.99729	0.70544
C	-2.91320	-1.82003	3.34513
H	-3.58728	-0.99481	3.04122
C	-2.91475	1.81956	-3.34476
H	-3.58830	0.99404	-3.04049
C	6.58228	-3.03756	-2.04935
H	6.65856	-3.74991	-1.20123
H	7.58061	-2.97996	-2.53382
H	5.87345	-3.47354	-2.78274
C	-7.36676	0.74164	-0.20702
H	-7.32261	1.40416	0.68712
H	-8.33296	0.99654	-0.70491
C	6.16897	0.11064	2.93920
H	5.65651	0.50461	3.84082
H	7.23377	-0.06102	3.20855
H	5.71941	-0.87572	2.70050
C	1.51790	1.19985	3.55654
H	1.53358	0.46158	4.38644
H	1.18829	0.66234	2.64089
H	0.75554	1.96823	3.81301
C	-6.16823	-0.11134	2.93987
H	-5.65551	-0.50509	3.84145
H	-7.23301	0.06014	3.20940
H	-5.71890	0.87507	2.70095
C	-2.14452	4.99695	-1.33261
H	-1.57531	5.92986	-1.47516
C	-6.58179	-3.03829	2.04930
H	-6.65907	-3.75019	1.20090
H	-7.57965	-2.98071	2.53475
H	-5.87237	-3.47483	2.78179
C	3.45302	-3.02719	2.51411
H	2.91321	-2.08146	2.28257
H	4.02000	-2.86920	3.45638
H	2.69563	-3.81894	2.70744
C	-4.39350	-3.42242	-1.35485
H	-5.08129	-2.56769	-1.18619
C	3.45157	-2.42394	-4.65961
H	4.46385	-2.85784	-4.52775
H	3.51588	-1.64563	-5.44951
H	2.79173	-3.23055	-5.04596
C	-6.16909	0.11002	-2.93877
H	-5.65709	0.50419	-3.84056
H	-7.23390	-0.06214	-3.20780
H	-5.71902	-0.87613	-2.70016
C	-1.51723	-1.19953	3.55628
H	-1.53297	-0.46140	4.38631
H	-1.18824	-0.66167	2.64061
H	-0.75439	-1.96756	3.81233
C	-3.45425	3.02756	2.51334

H	-2.69655	3.81904	2.70659
H	-2.91477	2.08166	2.28166
H	-4.02115	2.86971	3.45568
C	3.45364	3.02740	-2.51326
H	2.69589	3.81884	-2.70652
H	2.91426	2.08144	-2.28162
H	4.02054	2.86961	-3.45561
C	5.24468	4.65289	-1.72673
H	5.87542	4.43406	-2.61409
H	5.91768	4.94986	-0.89603
H	4.61930	5.53536	-1.98244
C	3.45383	2.42160	4.65900
H	2.79555	3.22916	5.04604
H	4.46661	2.85402	4.52620
H	3.51776	1.64310	5.44876
C	-5.24371	-4.65308	-1.72650
H	-4.61824	-5.53546	-1.98231
H	-5.87462	-4.43433	-2.61375
H	-5.91653	-4.95015	-0.89568
C	-1.51855	1.19975	-3.55640
H	-1.53426	0.46148	-4.38631
H	-1.18891	0.66221	-2.64078
H	-0.75621	1.96813	-3.81291
C	-3.45303	-3.02731	-2.51329
H	-2.91378	-2.08125	-2.28173
H	-4.02012	-2.86962	-3.45554
H	-2.69517	-3.81860	-2.70667
C	-3.45221	-2.42229	4.65929
H	-2.79342	-3.22955	5.04608
H	-4.46482	-2.85520	4.52678
H	-3.51627	-1.64387	5.44911
C	-5.24529	4.65305	1.72677
H	-5.87603	4.43425	2.61414
H	-5.91829	4.95001	0.89606
H	-4.61989	5.53552	1.98247
C	-3.45458	2.42139	-4.65879
H	-2.79635	3.22897	-5.04589
H	-4.46736	2.85378	-4.52594
H	-3.51852	1.64286	-5.44851

Cs(0)

Method A & B

SCF (BP86/BS1) Energy =
-20.1880347311

Enthalpy 0K = -20.188035

Enthalpy 298K = -20.185674

Free Energy 298K = -20.205603

SCF (BP86-D3^{BJ}) Energy =
-20.18803473

SCF (C_6H_6) Energy = -20.18979028

SCF (BS2) Energy = -20.18803473

SCF (BP86-D3^{BJ}(C_6H_6)/BS3) Energy =
-7916.40420542-

Cs	0.00000	0.00000	0.00000

Method C & D
SCF (BP86/BS4) Energy =
-20.1884145529
Enthalpy 0K = -20.188415
Enthalpy 298K = -20.186054
Free Energy 298K = -20.205983
SCF (BP86-D3^{BJ}) Energy =
-20.1884145529
SCF (PCM=C_6H_6) Energy = -20.190246
SCF (SMD=C_6H_6) Energy = -20.190490
SCF (BS5) Energy = -20.1884145529

Cs 0.00000 0.00000 0.00000
