# Supplementary material for: An Alumanyl Test Case of Group 1 Redox Interchange
Source: Chemistry. 2025 Jul 21;31(44):e202502197. doi: 10.1002/chem.202502197 (PMC12336768; doi:10.1002/chem.202502197)

checkCIF/PLATON report

You have not supplied any structure factors. As a result the full set of tests cannot be run.

THIS REPORT IS FOR GUIDANCE ONLY. IF USED AS PART OF A REVIEW PROCEDURE FOR PUBLICATION, IT SHOULD NOT REPLACE THE EXPERTISE OF AN EXPERIENCED CRYSTALLOGRAPHIC REFEREE.

No syntax errors found. CIF dictionary Interpreting this report

Datablock: s25msh15

Bond precision:	C-C = 0.0042 A		Wavelength=1.54184
Cell:	a=9.8944(1)	b=18.1117(1)	c=12.4161(1)
	alpha=90	beta=110.383(1)	gamma=90
Temperature:	150 K		

	Calculated	Reported
Volume	2085.70(3)	2085.70(3)
Space group	P 21	P 1 21 1
Hall group	P 2yb	P 2yb
Moiety formula	C30 H50 Al N2 Si2, C8 H16 Li O2	C38 H66 Al Li N2 O2 Si2
Sum formula	C38 H66 Al Li N2 O2 Si2	C38 H66 Al Li N2 O2 Si2
Mr	673.03	673.02
Dx,g cm-3	1.072	1.072
Z	2	2
Mu (mm-1)	1.206	1.206
F000	736.0	736.0
F000’	739.03	
h,k,lmax	12,22,15	12,22,15
Nref	8368[4323]	7092
Tmin,Tmax	0.568,0.672	0.731,1.000
Tmin’	0.515	

Correction method= # Reported T Limits: Tmin=0.731 Tmax=1.000
AbsCorr = MULTI-SCAN

Data completeness= 1.64/0.85 Theta(max)= 73.213

R(reflections)= 0.0394(7052) wR2(reflections)= 0.1070(7092)

S = 1.086 Npar= 462

The following ALERTS were generated. Each ALERT has the format
test-name_ALERT_alert-type_alert-level.
Click on the hyperlinks for more details of the test.

Alert level C

```
PLAT042_ALERT_1_C Calc. and Reported MoietyFormula Strings  Differ     Please Check
              Calc: C30 H50 Al N2 Si2, C8 H16 Li O2
              Rep.: C38 H66 Al Li N2 O2 Si2
PLAT241_ALERT_2_C High   'MainMol' Ueq as Compared to Neighbors of        C32 Check
PLAT241_ALERT_2_C High   'MainMol' Ueq as Compared to Neighbors of        C36 Check
PLAT340_ALERT_3_C Low Bond Precision on  C-C Bonds ..............    0.00424 Ang.
```

Alert level G

```
PLAT002_ALERT_2_G Number of Distance or Angle Restraints on AtSite         14 Note
PLAT003_ALERT_2_G Number of Uiso or U(i,j)  Restrained non-H-Atoms         12 Report
PLAT033_ALERT_4_G Flack x Value Deviates > 3.0 * Sigma from Zero .      0.190 Note
PLAT142_ALERT_4_G s.u. on b - Axis Small or Missing .............    0.00010 Ang.
PLAT143_ALERT_4_G s.u. on c - Axis Small or Missing .............    0.00010 Ang.
PLAT153_ALERT_1_G The s.u.'s on the Cell Axes   are Equal ..(Note)     0.0001 Ang.
PLAT176_ALERT_4_G The CIF-Embedded .res File Contains SADI Records         12 Report
PLAT178_ALERT_4_G The CIF-Embedded .res File Contains SIMU Records          6 Report
PLAT188_ALERT_3_G A Non-default SIMU Restraint Value has been used     0.0200 Report
PLAT188_ALERT_3_G A Non-default SIMU Restraint Value has been used     0.0200 Report
PLAT188_ALERT_3_G A Non-default SIMU Restraint Value has been used     0.0200 Report
PLAT188_ALERT_3_G A Non-default SIMU Restraint Value has been used     0.0200 Report
PLAT188_ALERT_3_G A Non-default SIMU Restraint Value has been used     0.0200 Report
PLAT188_ALERT_3_G A Non-default SIMU Restraint Value has been used     0.0200 Report
PLAT191_ALERT_3_G A Non-default SADI Restraint Value has been used     0.0400 Report
PLAT191_ALERT_3_G A Non-default SADI Restraint Value has been used     0.0050 Report
PLAT191_ALERT_3_G A Non-default SADI Restraint Value has been used     0.0050 Report
PLAT191_ALERT_3_G A Non-default SADI Restraint Value has been used     0.0050 Report
PLAT191_ALERT_3_G A Non-default SADI Restraint Value has been used     0.0400 Report
PLAT191_ALERT_3_G A Non-default SADI Restraint Value has been used     0.0050 Report
PLAT191_ALERT_3_G A Non-default SADI Restraint Value has been used     0.0050 Report
PLAT191_ALERT_3_G A Non-default SADI Restraint Value has been used     0.0050 Report
PLAT300_ALERT_4_G Atom Site Occupancy of C13        Constrained at        0.6 Check
PLAT300_ALERT_4_G Atom Site Occupancy of C14        Constrained at        0.6 Check
PLAT300_ALERT_4_G Atom Site Occupancy of C15        Constrained at        0.6 Check
PLAT300_ALERT_4_G Atom Site Occupancy of C16A       Constrained at        0.6 Check
PLAT300_ALERT_4_G Atom Site Occupancy of C17A       Constrained at        0.6 Check
PLAT300_ALERT_4_G Atom Site Occupancy of C18A       Constrained at        0.6 Check
PLAT300_ALERT_4_G Atom Site Occupancy of C13A       Constrained at        0.4 Check
PLAT300_ALERT_4_G Atom Site Occupancy of C14A       Constrained at        0.4 Check
PLAT300_ALERT_4_G Atom Site Occupancy of C15A       Constrained at        0.4 Check
PLAT300_ALERT_4_G Atom Site Occupancy of C16        Constrained at        0.4 Check
PLAT300_ALERT_4_G Atom Site Occupancy of C17        Constrained at        0.4 Check
PLAT300_ALERT_4_G Atom Site Occupancy of C18        Constrained at        0.4 Check
PLAT300_ALERT_4_G Atom Site Occupancy of H13        Constrained at        0.6 Check
PLAT300_ALERT_4_G Atom Site Occupancy of H14A       Constrained at        0.6 Check
PLAT300_ALERT_4_G Atom Site Occupancy of H14B       Constrained at        0.6 Check
PLAT300_ALERT_4_G Atom Site Occupancy of H14C       Constrained at        0.6 Check
PLAT300_ALERT_4_G Atom Site Occupancy of H15A       Constrained at        0.6 Check
PLAT300_ALERT_4_G Atom Site Occupancy of H15B       Constrained at        0.6 Check
PLAT300_ALERT_4_G Atom Site Occupancy of H15C       Constrained at        0.6 Check
```

```
PLAT300_ALERT_4_G Atom Site Occupancy of H16A       Constrained at       0.6 Check
PLAT300_ALERT_4_G Atom Site Occupancy of H17D       Constrained at       0.6 Check
PLAT300_ALERT_4_G Atom Site Occupancy of H17E       Constrained at       0.6 Check
PLAT300_ALERT_4_G Atom Site Occupancy of H17F       Constrained at       0.6 Check
PLAT300_ALERT_4_G Atom Site Occupancy of H18D       Constrained at       0.6 Check
PLAT300_ALERT_4_G Atom Site Occupancy of H18E       Constrained at       0.6 Check
PLAT300_ALERT_4_G Atom Site Occupancy of H18F       Constrained at       0.6 Check
PLAT300_ALERT_4_G Atom Site Occupancy of H13A       Constrained at       0.4 Check
PLAT300_ALERT_4_G Atom Site Occupancy of H14D       Constrained at       0.4 Check
PLAT300_ALERT_4_G Atom Site Occupancy of H14E       Constrained at       0.4 Check
PLAT300_ALERT_4_G Atom Site Occupancy of H14F       Constrained at       0.4 Check
PLAT300_ALERT_4_G Atom Site Occupancy of H15D       Constrained at       0.4 Check
PLAT300_ALERT_4_G Atom Site Occupancy of H15E       Constrained at       0.4 Check
PLAT300_ALERT_4_G Atom Site Occupancy of H15F       Constrained at       0.4 Check
PLAT300_ALERT_4_G Atom Site Occupancy of H16        Constrained at       0.4 Check
PLAT300_ALERT_4_G Atom Site Occupancy of H17A       Constrained at       0.4 Check
PLAT300_ALERT_4_G Atom Site Occupancy of H17B       Constrained at       0.4 Check
PLAT300_ALERT_4_G Atom Site Occupancy of H17C       Constrained at       0.4 Check
PLAT300_ALERT_4_G Atom Site Occupancy of H18A       Constrained at       0.4 Check
PLAT300_ALERT_4_G Atom Site Occupancy of H18B       Constrained at       0.4 Check
PLAT300_ALERT_4_G Atom Site Occupancy of H18C       Constrained at       0.4 Check
PLAT301_ALERT_3_G Main Residue  Disorder .............(Resd    1)        17% Note
PLAT412_ALERT_2_G Short Intra XH3 .. XHn     H6A      ..H16A     .      2.06 Ang.
                                                       x,y,z  =     1_555 Check
PLAT412_ALERT_2_G Short Intra XH3 .. XHn     H6A      ..H16      .      2.06 Ang.
                                                       x,y,z  =     1_555 Check
PLAT412_ALERT_2_G Short Intra XH3 .. XHn     H9       ..H14D     .      1.97 Ang.
                                                       x,y,z  =     1_555 Check
PLAT860_ALERT_3_G Number of Least-Squares Restraints ............         49 Note
```

---

0 **ALERT level A** = Most likely a serious problem - resolve or explain
0 **ALERT level B** = A potentially serious problem, consider carefully
4 **ALERT level C** = Check. Ensure it is not caused by an omission or oversight
67 **ALERT level G** = General information/check it is not something unexpected

2 ALERT type 1 CIF construction/syntax error, inconsistent or missing data
7 ALERT type 2 Indicator that the structure model may be wrong or deficient
17 ALERT type 3 Indicator that the structure quality may be low
45 ALERT type 4 Improvement, methodology, query or suggestion
0 ALERT type 5 Informative message, check

It is advisable to attempt to resolve as many as possible of the alerts in all categories. Often the minor alerts point to easily fixed oversights, errors and omissions in your CIF or refinement strategy, so attention to these fine details can be worthwhile. In order to resolve some of the more serious problems it may be necessary to carry out additional measurements or structure refinements. However, the purpose of your study may justify the reported deviations and the more serious of these should normally be commented upon in the discussion or experimental section of a paper or in the "special_details" fields of the CIF. checkCIF was carefully designed to identify outliers and unusual parameters, but every test has its limitations and alerts that are not important in a particular case may appear. Conversely, the absence of alerts does not guarantee there are no aspects of the results needing attention. It is up to the individual to critically assess their own results and, if necessary, seek expert advice.

Publication of your CIF in IUCr journals

A basic structural check has been run on your CIF. These basic checks will be run on all CIFs submitted for publication in IUCr journals (*Acta Crystallographica*, *Journal of Applied Crystallography*, *Journal of Synchrotron Radiation*); however, if you intend to submit to *Acta Crystallographica Section C* or *E* or *IUCrData*, you should make sure that full publication checks are run on the final version of your CIF prior to submission.

Publication of your CIF in other journals

Please refer to the *Notes for Authors* of the relevant journal for any special instructions relating to CIF submission.

PLATON version of 02/02/2025; check.def file version of 02/02/2025

Datablock s25msh15 - ellipsoid plot

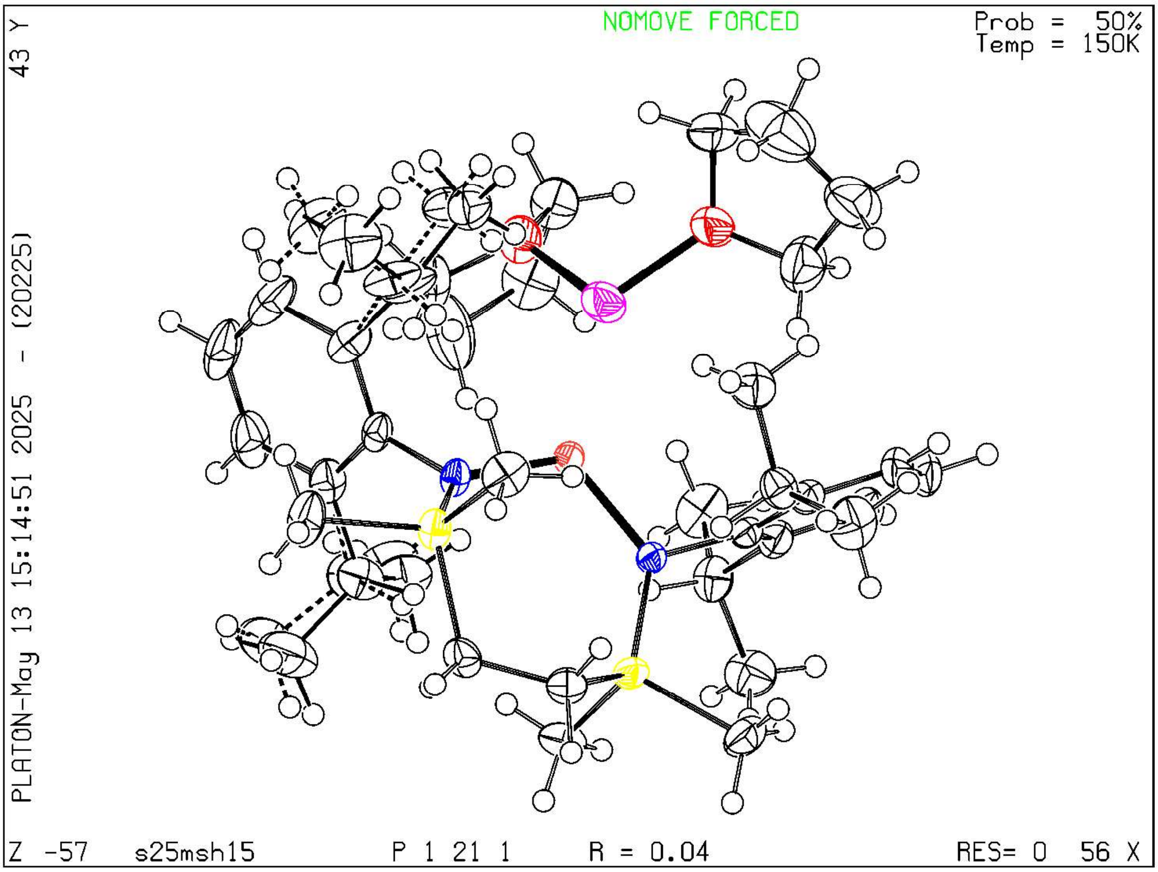

checkCIF/PLATON report

You have not supplied any structure factors. As a result the full set of tests cannot be run.

THIS REPORT IS FOR GUIDANCE ONLY. IF USED AS PART OF A REVIEW PROCEDURE FOR PUBLICATION, IT SHOULD NOT REPLACE THE EXPERTISE OF AN EXPERIENCED CRYSTALLOGRAPHIC REFEREE.

No syntax errors found.   CIF dictionary   Interpreting this report

Datablock: s25msh03

Bond precision: C-C = 0.0041 A   Wavelength=1.54184

Cell:	a=10.0918(3)	b=11.8653(4)	c=16.1802(6)
	alpha=72.810(3)	beta=84.514(3)	gamma=81.842(3)
Temperature:	150 K		

	Calculated	Reported
Volume	1829.23(11)	1829.23(11)
Space group	P -1	P -1
Hall group	-P 1	-P 1
Moiety formula	C30 H50 Al Li N2 Si2, C6 H6	C60 H100 Al2 Li2 N4 Si4, 2(C6 H6)
Sum formula	C36 H56 Al Li N2 Si2	C72 H112 Al2 Li2 N4 Si4
Mr	606.93	1213.85
Dx,g cm-3	1.102	1.102
Z	2	1
Mu (mm-1)	1.289	1.289
F000	660.0	660.0
F000'	662.77	
h,k,lmax	12,14,20	12,14,20
Nref	7338	7167
Tmin,Tmax	0.757,0.902	0.604,1.000
Tmin'	0.743	

Correction method= # Reported T Limits: Tmin=0.604 Tmax=1.000
AbsCorr = MULTI-SCAN

Data completeness= 0.977   Theta(max)= 73.179

R(reflections)= 0.0554(5739)   wR2(reflections)= 0.1500(7167)

S = 1.029   Npar= 391

The following ALERTS were generated. Each ALERT has the format
test-name_ALERT_alert-type_alert-level.
Click on the hyperlinks for more details of the test.

Alert level C

```
PLAT041_ALERT_1_C Calc. and Reported SumFormula    Strings  Differ     Please Check
              Calc: C36 H56 Al Li N2 Si2
              Rep.: C72 H112 Al2 Li2 N4 Si4
PLAT042_ALERT_1_C Calc. and Reported MoietyFormula Strings  Differ     Please Check
              Calc: C30 H50 Al Li N2 Si2, C6 H6
              Rep.: C60 H100 Al2 Li2 N4 Si4, 2(C6 H6)
PLAT242_ALERT_2_C Low    'MainMol' Ueq as Compared to Neighbors of        C13 Check
PLAT340_ALERT_3_C Low Bond Precision on  C-C Bonds ...............    0.00413 Ang.
```

Alert level G

```
PLAT003_ALERT_2_G Number of Uiso or U(i,j)  Restrained non-H-Atoms          6 Report
PLAT045_ALERT_1_G Calculated and Reported Z Differ by a Factor ...          2 Check
PLAT154_ALERT_1_G The s.u.'s on the Cell Angles are Equal ..(Note)      0.003 Degree
PLAT178_ALERT_4_G The CIF-Embedded .res File Contains SIMU Records          1 Report
PLAT188_ALERT_3_G A Non-default SIMU Restraint Value has been used     0.0020 Report
PLAT343_ALERT_2_G Unusual          Angle Range in Main Residue for         C9 Check
PLAT343_ALERT_2_G Unusual          Angle Range in Main Residue for        C10 Check
PLAT343_ALERT_2_G Unusual          Angle Range in Main Residue for        C11 Check
PLAT764_ALERT_4_G Overcomplete CIF Bond List Detected (Rep/Expd) .       1.11 Ratio
PLAT860_ALERT_3_G Number of Least-Squares Restraints .............         36 Note
PLAT941_ALERT_3_G Average HKL Measurement Multiplicity ..........         1.9 Low
```

0 **ALERT level A** = Most likely a serious problem - resolve or explain
0 **ALERT level B** = A potentially serious problem, consider carefully
4 **ALERT level C** = Check. Ensure it is not caused by an omission or oversight
11 **ALERT level G** = General information/check it is not something unexpected

4 ALERT type 1 CIF construction/syntax error, inconsistent or missing data
5 ALERT type 2 Indicator that the structure model may be wrong or deficient
4 ALERT type 3 Indicator that the structure quality may be low
2 ALERT type 4 Improvement, methodology, query or suggestion
0 ALERT type 5 Informative message, check

It is advisable to attempt to resolve as many as possible of the alerts in all categories. Often the minor alerts point to easily fixed oversights, errors and omissions in your CIF or refinement strategy, so attention to these fine details can be worthwhile. In order to resolve some of the more serious problems it may be necessary to carry out additional measurements or structure refinements. However, the purpose of your study may justify the reported deviations and the more serious of these should normally be commented upon in the discussion or experimental section of a paper or in the "special_details" fields of the CIF. checkCIF was carefully designed to identify outliers and unusual parameters, but every test has its limitations and alerts that are not important in a particular case may appear. Conversely, the absence of alerts does not guarantee there are no aspects of the results needing attention. It is up to the individual to critically assess their own results and, if necessary, seek expert advice.

Publication of your CIF in IUCr journals

A basic structural check has been run on your CIF. These basic checks will be run on all CIFs submitted for publication in IUCr journals (*Acta Crystallographica*, *Journal of Applied Crystallography*, *Journal of Synchrotron Radiation*); however, if you intend to submit to *Acta Crystallographica Section C* or *E* or *IUCrData*, you should make sure that full publication checks are run on the final version of your CIF prior to submission.

Publication of your CIF in other journals

Please refer to the *Notes for Authors* of the relevant journal for any special instructions relating to CIF submission.

PLATON version of 02/02/2025; check.def file version of 02/02/2025

Datablock s25msh03 - ellipsoid plot

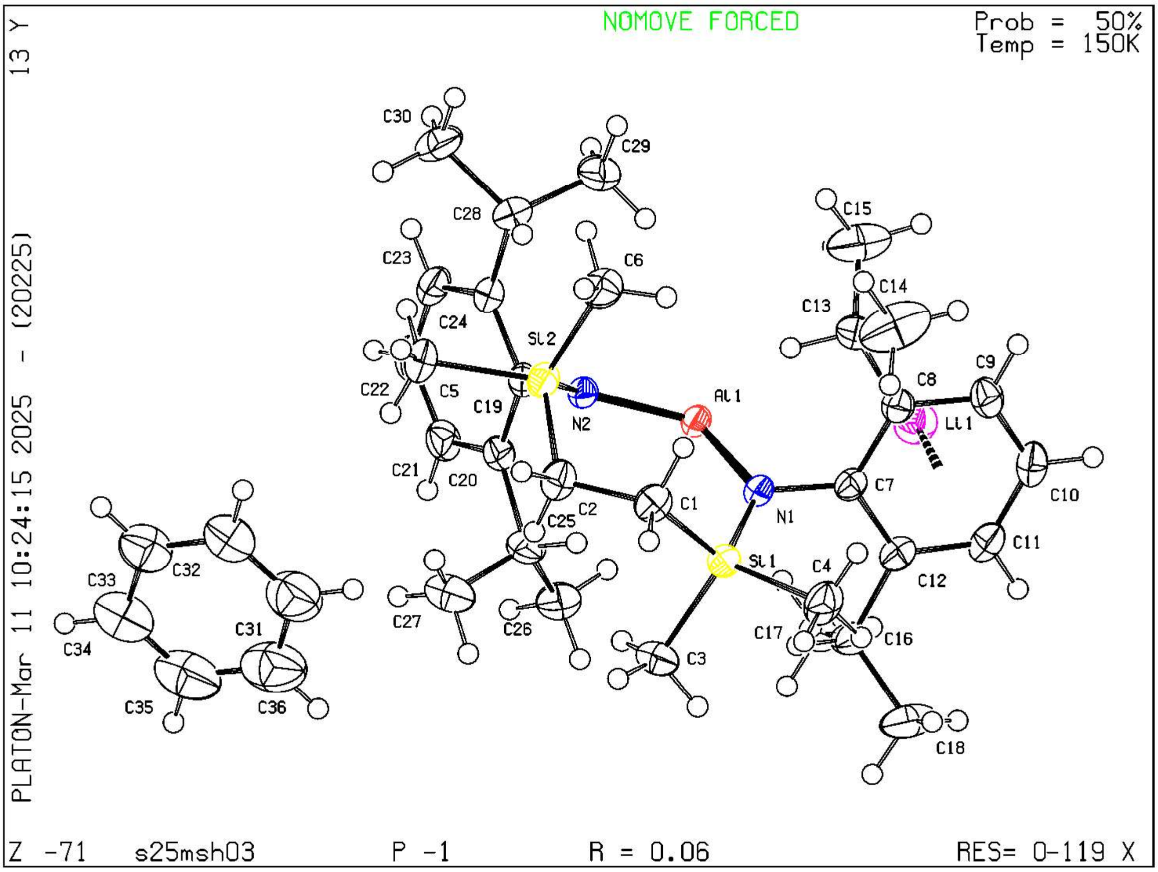

checkCIF/PLATON report

Structure factors have been supplied for datablock(s) e23msh07

THIS REPORT IS FOR GUIDANCE ONLY. IF USED AS PART OF A REVIEW PROCEDURE FOR PUBLICATION, IT SHOULD NOT REPLACE THE EXPERTISE OF AN EXPERIENCED CRYSTALLOGRAPHIC REFEREE.

No syntax errors found.   CIF dictionary   Interpreting this report

Datablock: e23msh07

Bond precision: C-C = 0.0034 A   Wavelength=0.71073

Cell:	a=12.1789(4)	b=12.1827(4)	c=12.3361(5)
	alpha=88.756(3)	beta=78.031(3)	gamma=77.850(3)
Temperature:	150 K		

	Calculated	Reported
Volume	1749.97(11)	1749.97(11)
Space group	P -1	P -1
Hall group	-P 1	-P 1
Moiety formula	C60 H100 Cl4 Li2 N4 Si4 Y2	C30 H50 Cl2 Li N2 Si2 Y
Sum formula	C60 H100 Cl4 Li2 N4 Si4 Y2	C30 H50 Cl2 Li N2 Si2 Y
Mr	1323.30	661.65
Dx,g cm-3	1.256	1.256
Z	1	2
Mu (mm-1)	1.908	1.908
F000	696.0	696.0
F000'	691.46	
h,k,lmax	17,17,17	17,16,16
Nref	10616	8868
Tmin,Tmax	0.496,0.599	0.914,1.000
Tmin'	0.486	

Correction method= # Reported T Limits: Tmin=0.914 Tmax=1.000
AbsCorr = MULTI-SCAN

Data completeness= 0.835   Theta(max)= 30.437

R(reflections)= 0.0385(7261)   wR2(reflections)= 0.0813(8868)

S = 1.032   Npar= 355

The following ALERTS were generated. Each ALERT has the format
test-name_ALERT_alert-type_alert-level.
Click on the hyperlinks for more details of the test.

Alert level B

```
PLAT910_ALERT_3_B Missing # of FCF Reflection(s) Below Theta(Min).         11 Note
                0  1  0,   1 -1  0,   1  0  0,   1  1  0,  -1 -1  1,  -1  0  1,
                0 -1  1,   0  0  1,   0  1  1,   1  0  1,   1  1  1,
```

Alert level C

```
PLAT041_ALERT_1_C Calc. and Reported SumFormula    Strings  Differ     Please Check
              Calc: C60 H100 Cl4 Li2 N4 Si4 Y2
              Rep.: C30 H50 Cl2 Li N2 Si2 Y
PLAT042_ALERT_1_C Calc. and Reported MoietyFormula Strings  Differ     Please Check
              Calc: C60 H100 Cl4 Li2 N4 Si4 Y2
              Rep.: C30 H50 Cl2 Li N2 Si2 Y
PLAT220_ALERT_2_C NonSolvent   Resd 1  C   Ueq(max)/Ueq(min) Range        3.1 Ratio
```

Alert level G

```
PLAT045_ALERT_1_G Calculated and Reported Z Differ by a Factor ...      0.500 Check
PLAT154_ALERT_1_G The s.u.'s on the Cell Angles are Equal ..(Note)      0.003 Degree
PLAT232_ALERT_2_G Hirshfeld Test Diff (M-X)  Y1       --Cl1      .        5.3 s.u.
PLAT232_ALERT_2_G Hirshfeld Test Diff (M-X)  Y1       --Cl2_a    .        8.3 s.u.
PLAT343_ALERT_2_G Unusual          Angle Range in Main Residue for        C22 Check
PLAT912_ALERT_4_G Missing # of FCF Reflections Above STh/L=  0.600       1641 Note
PLAT941_ALERT_3_G Average HKL Measurement Multiplicity ..........         1.9 Low
PLAT969_ALERT_5_G The 'Henn et al.' R-Factor-gap value ..........       1.947 Note
              Predicted wR2: Based on SigI**2  4.17 or SHELX Weight  7.87
PLAT978_ALERT_2_G Number C-C Bonds with Positive Residual Density.          5 Info
```

---

0 **ALERT level A** = Most likely a serious problem - resolve or explain
1 **ALERT level B** = A potentially serious problem, consider carefully
3 **ALERT level C** = Check. Ensure it is not caused by an omission or oversight
9 **ALERT level G** = General information/check it is not something unexpected

4 ALERT type 1 CIF construction/syntax error, inconsistent or missing data
5 ALERT type 2 Indicator that the structure model may be wrong or deficient
2 ALERT type 3 Indicator that the structure quality may be low
1 ALERT type 4 Improvement, methodology, query or suggestion
1 ALERT type 5 Informative message, check

Validation response form

Please find below a validation response form (VRF) that can be filled in and pasted into your CIF.

```
# start Validation Reply Form
_vrf_PLAT910_e23msh07
;
PROBLEM: Missing # of FCF Reflection(s) Below Theta(Min).         11 Note
RESPONSE: ...
```

```
;
_vrf_PLAT041_e23msh07
;
PROBLEM: Calc. and Reported SumFormula    Strings  Differ     Please Check
RESPONSE: ...
;
_vrf_PLAT042_e23msh07
;
PROBLEM: Calc. and Reported MoietyFormula Strings  Differ     Please Check
RESPONSE: ...
;
_vrf_PLAT220_e23msh07
;
PROBLEM: NonSolvent   Resd 1  C   Ueq(max)/Ueq(min) Range        3.1 Ratio
RESPONSE: ...
;
# end Validation Reply Form
```

It is advisable to attempt to resolve as many as possible of the alerts in all categories. Often the minor alerts point to easily fixed oversights, errors and omissions in your CIF or refinement strategy, so attention to these fine details can be worthwhile. In order to resolve some of the more serious problems it may be necessary to carry out additional measurements or structure refinements. However, the purpose of your study may justify the reported deviations and the more serious of these should normally be commented upon in the discussion or experimental section of a paper or in the "special_details" fields of the CIF. checkCIF was carefully designed to identify outliers and unusual parameters, but every test has its limitations and alerts that are not important in a particular case may appear. Conversely, the absence of alerts does not guarantee there are no aspects of the results needing attention. It is up to the individual to critically assess their own results and, if necessary, seek expert advice.

Publication of your CIF in IUCr journals

A basic structural check has been run on your CIF. These basic checks will be run on all CIFs submitted for publication in IUCr journals (*Acta Crystallographica*, *Journal of Applied Crystallography*, *Journal of Synchrotron Radiation*); however, if you intend to submit to *Acta Crystallographica Section C* or *E* or *IUCrData*, you should make sure that full publication checks are run on the final version of your CIF prior to submission.

Publication of your CIF in other journals

Please refer to the *Notes for Authors* of the relevant journal for any special instructions relating to CIF submission.

PLATON version of 02/02/2025; check.def file version of 02/02/2025

Datablock e23msh07 - ellipsoid plot

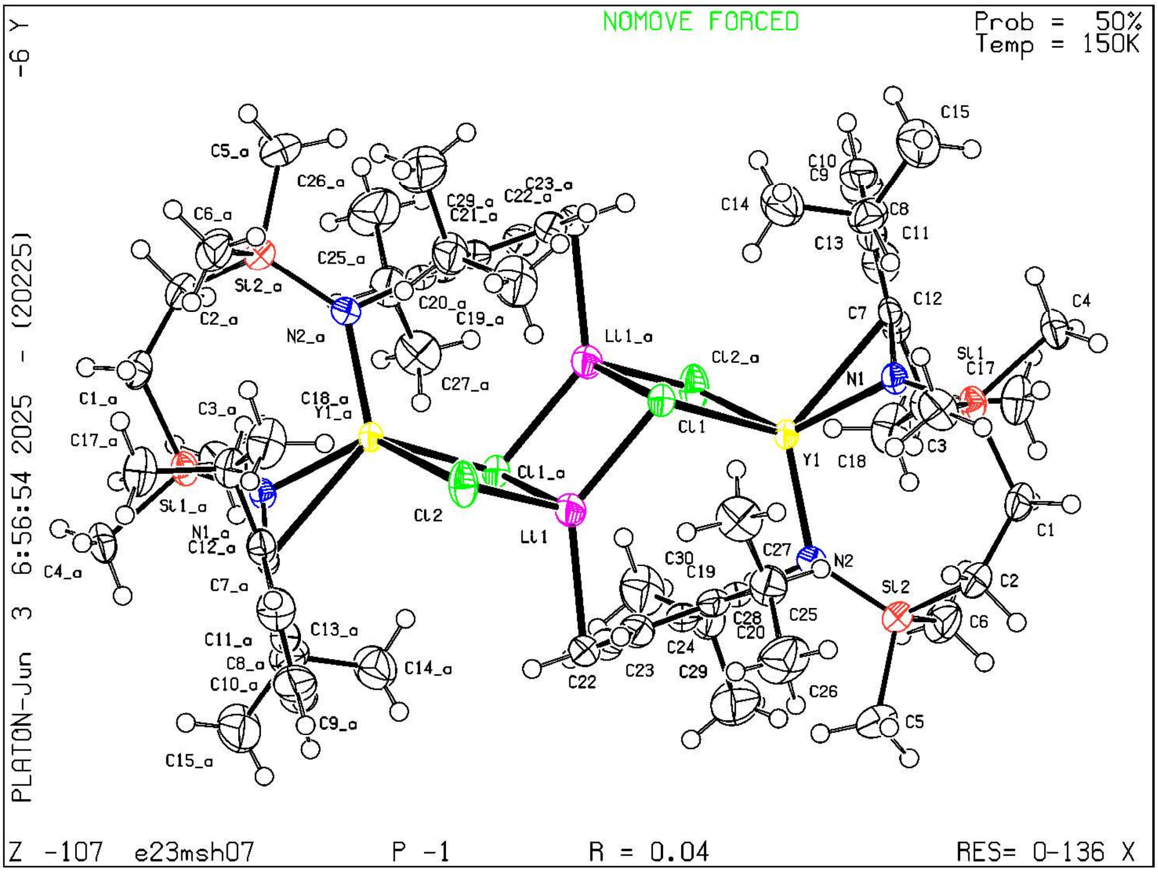

checkCIF/PLATON report

You have not supplied any structure factors. As a result the full set of tests cannot be run.

THIS REPORT IS FOR GUIDANCE ONLY. IF USED AS PART OF A REVIEW PROCEDURE FOR PUBLICATION, IT SHOULD NOT REPLACE THE EXPERTISE OF AN EXPERIENCED CRYSTALLOGRAPHIC REFEREE.

No syntax errors found.   CIF dictionary   Interpreting this report

Datablock: y25msh10

Bond precision: C-C = 0.0036 A   Wavelength=1.54184

Cell:	a=9.7301(1)	b=12.7122(2)	c=15.8207(3)
	alpha=69.127(2)	beta=83.407(1)	gamma=76.470(1)
Temperature:	150 K		

	Calculated	Reported
Volume	1776.64(5)	1776.64(5)
Space group	P -1	P -1
Hall group	-P 1	-P 1
Moiety formula	C30 H50 Cl2 N2 Rb Si2 Y	C30 H50 Cl2 N2 Rb Si2 Y
Sum formula	C30 H50 Cl2 N2 Rb Si2 Y	C30 H50 Cl2 N2 Rb Si2 Y
Mr	740.18	740.18
Dx,g cm-3	1.384	1.384
Z	2	2
Mu (mm-1)	6.146	6.146
F000	764.0	764.0
F000'	766.13	
h,k,lmax	12,16,20	12,16,20
Nref	7754	7453
Tmin,Tmax	0.541,0.650	0.776,1.000
Tmin'	0.379	

Correction method= # Reported T Limits: Tmin=0.776 Tmax=1.000
AbsCorr = MULTI-SCAN

Data completeness= 0.961   Theta(max)= 79.966

R(reflections)= 0.0271(7152)   wR2(reflections)= 0.0725(7453)

S = 1.092   Npar= 366

The following ALERTS were generated. Each ALERT has the format
test-name_ALERT_alert-type_alert-level.
Click on the hyperlinks for more details of the test.

Alert level G

```
PLAT004_ALERT_5_G Polymeric Structure Found with Maximum Dimension          1 Info
PLAT164_ALERT_4_G Nr. of Refined C-H H-Atoms in Heavy-Atom Struct.          3 Note
PLAT232_ALERT_2_G Hirshfeld Test Diff (M-X)  Y1       --Cl1      .        6.0 s.u.
PLAT232_ALERT_2_G Hirshfeld Test Diff (M-X)  Y1       --Cl2      .        9.7 s.u.
PLAT232_ALERT_2_G Hirshfeld Test Diff (M-X)  Y1       --Si1      .        8.7 s.u.
PLAT764_ALERT_4_G Overcomplete CIF Bond List Detected (Rep/Expd) .       1.12 Ratio
PLAT941_ALERT_3_G Average HKL Measurement Multiplicity ..........         3.6 Low
```

0 **ALERT level A** = Most likely a serious problem - resolve or explain
0 **ALERT level B** = A potentially serious problem, consider carefully
0 **ALERT level C** = Check. Ensure it is not caused by an omission or oversight
7 **ALERT level G** = General information/check it is not something unexpected

0 ALERT type 1 CIF construction/syntax error, inconsistent or missing data
3 ALERT type 2 Indicator that the structure model may be wrong or deficient
1 ALERT type 3 Indicator that the structure quality may be low
2 ALERT type 4 Improvement, methodology, query or suggestion
1 ALERT type 5 Informative message, check

It is advisable to attempt to resolve as many as possible of the alerts in all categories. Often the minor alerts point to easily fixed oversights, errors and omissions in your CIF or refinement strategy, so attention to these fine details can be worthwhile. In order to resolve some of the more serious problems it may be necessary to carry out additional measurements or structure refinements. However, the purpose of your study may justify the reported deviations and the more serious of these should normally be commented upon in the discussion or experimental section of a paper or in the "special_details" fields of the CIF. checkCIF was carefully designed to identify outliers and unusual parameters, but every test has its limitations and alerts that are not important in a particular case may appear. Conversely, the absence of alerts does not guarantee there are no aspects of the results needing attention. It is up to the individual to critically assess their own results and, if necessary, seek expert advice.

Publication of your CIF in IUCr journals

A basic structural check has been run on your CIF. These basic checks will be run on all CIFs submitted for publication in IUCr journals (*Acta Crystallographica*, *Journal of Applied Crystallography*, *Journal of Synchrotron Radiation*); however, if you intend to submit to *Acta Crystallographica Section C* or *E* or *IUCrData*, you should make sure that full publication checks are run on the final version of your CIF prior to submission.

Publication of your CIF in other journals

Please refer to the *Notes for Authors* of the relevant journal for any special instructions relating to CIF submission.

PLATON version of 02/02/2025; check.def file version of 02/02/2025

Datablock y25msh10 - ellipsoid plot

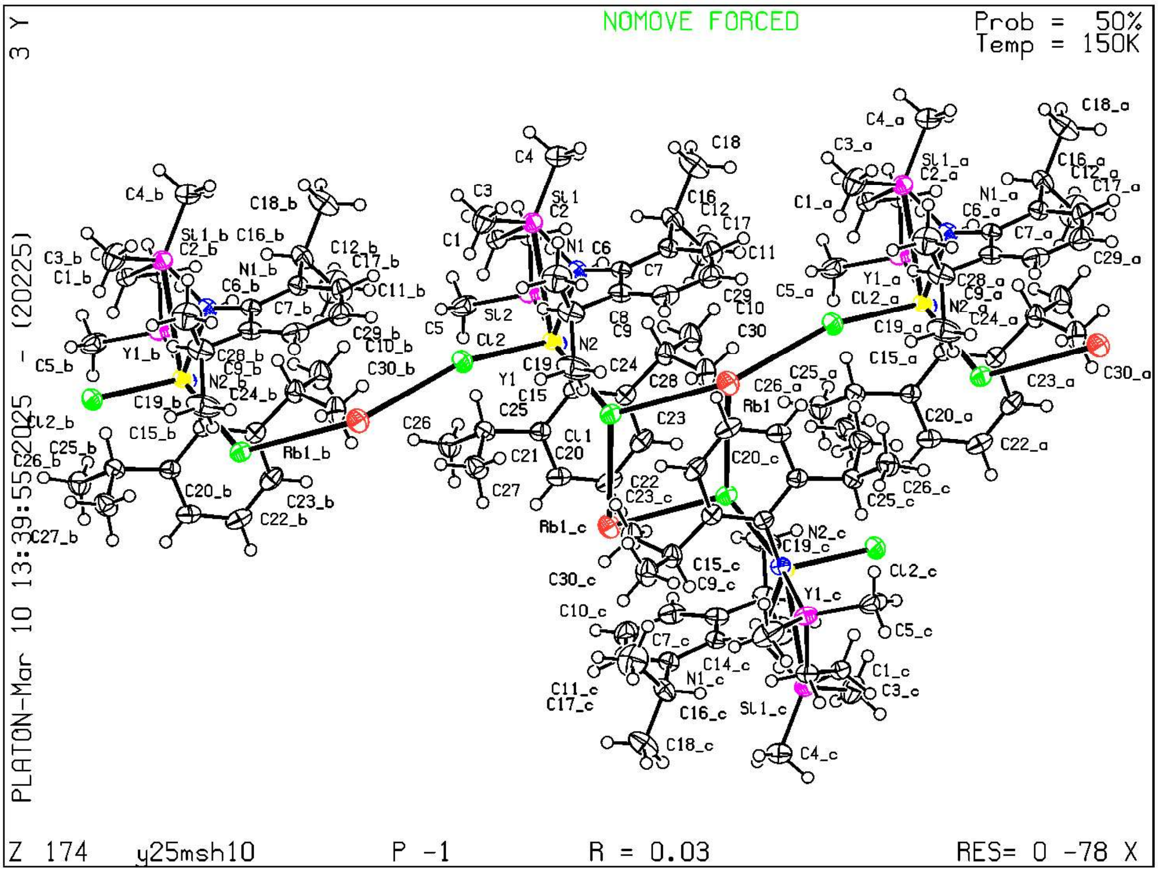

checkCIF/PLATON report

You have not supplied any structure factors. As a result the full set of tests cannot be run.

THIS REPORT IS FOR GUIDANCE ONLY. IF USED AS PART OF A REVIEW PROCEDURE FOR PUBLICATION, IT SHOULD NOT REPLACE THE EXPERTISE OF AN EXPERIENCED CRYSTALLOGRAPHIC REFEREE.

No syntax errors found. CIF dictionary Interpreting this report

Datablock: y25msh32

```
Bond precision:   C-C = 0.0148 A          Wavelength=1.54184

Cell:             a=16.4095(4)     b=13.1233(2)        c=16.8199(4)
                  alpha=90         beta=111.404(3)     gamma=90
Temperature:      150 K

                  Calculated                   Reported
Volume            3372.30(14)                  3372.30(14)
Space group       P 21                         P 1 21 1
Hall group        P 2yb                        P 2yb
Moiety formula    C30 H50 Al N2 Si2, Na        C60 H100 Al2 N4 Na2 Si4
Sum formula       C30 H50 Al N2 Na Si2         C60 H100 Al2 N4 Na2 Si4
Mr                544.87                       1089.73
Dx,g cm-3         1.073                        1.073
Z                 4                            2
Mu (mm-1)         1.468                        1.468
F000              1184.0                       1184.0
F000'             1189.64
h,k,lmax          20,16,21                     20,16,21
Nref              14280[ 7462]                 12062
Tmin,Tmax         0.809,0.929                  0.734,1.000
Tmin'             0.735

Correction method= # Reported T Limits: Tmin=0.734 Tmax=1.000
AbsCorr = MULTI-SCAN

Data completeness= 1.62/0.84       Theta(max)= 77.044

R(reflections)= 0.1103( 10317)                   wR2(reflections)=
                                                 0.3335( 12062)
S = 1.035                   Npar= 684
```

The following ALERTS were generated. Each ALERT has the format
test-name_ALERT_alert-type_alert-level.
Click on the hyperlinks for more details of the test.

Alert level B

```
PLAT340_ALERT_3_B Low Bond Precision on  C-C Bonds ..............     0.0148 Ang.
```

Author Response: This alert is a consequence of the disorder on the Dipp groups.

Alert level C

```
DIFMX02_ALERT_1_C  The maximum difference density is > 0.1*ZMAX*0.75
            The relevant atom site should be identified.
PLAT041_ALERT_1_C Calc. and Reported SumFormula    Strings  Differ     Please Check
              Calc: C30 H50 Al N2 Na Si2
              Rep.: C60 H100 Al2 N4 Na2 Si4
PLAT042_ALERT_1_C Calc. and Reported MoietyFormula Strings  Differ     Please Check
              Calc: C30 H50 Al N2 Si2, Na
              Rep.: C60 H100 Al2 N4 Na2 Si4
PLAT082_ALERT_2_C High R1 Value .................................       0.11 Report
PLAT084_ALERT_3_C High wR2 Value (i.e. > 0.25) ..................       0.33 Report
PLAT094_ALERT_2_C Ratio of Maximum / Minimum Residual Density ....       2.83 Report
PLAT097_ALERT_2_C Large Reported Max.  (Positive) Residual Density       1.41 eA-3
PLAT220_ALERT_2_C NonSolvent   Resd 1  C   Ueq(max)/Ueq(min) Range        4.8 Ratio
PLAT222_ALERT_3_C NonSolvent Resd 1  H   Uiso(max)/Uiso(min) Range        5.1 Ratio
PLAT234_ALERT_4_C Large Hirshfeld Difference C56      --C57      .       0.20 Ang.
PLAT242_ALERT_2_C Low    'MainMol' Ueq as Compared to Neighbors of        C47 Check
PLAT601_ALERT_2_C Unit Cell Contains Solvent Accessible VOIDS   <=         40 Ang**3
```

Alert level G

```
PLAT002_ALERT_2_G Number of Distance or Angle Restraints on AtSite         17 Note
PLAT003_ALERT_2_G Number of Uiso or U(i,j)  Restrained non-H-Atoms         10 Report
PLAT045_ALERT_1_G Calculated and Reported Z Differ by a Factor ...          2 Check
PLAT072_ALERT_2_G SHELXL First  Parameter in WGHT  Unusually Large       0.17 Report
PLAT083_ALERT_2_G SHELXL Second Parameter in WGHT  Unusually Large      21.50 Why ?
PLAT176_ALERT_4_G The CIF-Embedded .res File Contains SADI Records         14 Report
PLAT178_ALERT_4_G The CIF-Embedded .res File Contains SIMU Records          1 Report
PLAT186_ALERT_4_G The CIF-Embedded .res File Contains ISOR Records          8 Report
PLAT188_ALERT_3_G A Non-default SIMU Restraint Value has been used     0.0200 Report
PLAT191_ALERT_3_G A Non-default SADI Restraint Value has been used     0.0400 Report
PLAT191_ALERT_3_G A Non-default SADI Restraint Value has been used     0.0050 Report
PLAT191_ALERT_3_G A Non-default SADI Restraint Value has been used     0.0050 Report
PLAT191_ALERT_3_G A Non-default SADI Restraint Value has been used     0.0050 Report
PLAT191_ALERT_3_G A Non-default SADI Restraint Value has been used     0.0400 Report
PLAT191_ALERT_3_G A Non-default SADI Restraint Value has been used     0.0050 Report
PLAT191_ALERT_3_G A Non-default SADI Restraint Value has been used     0.0050 Report
PLAT191_ALERT_3_G A Non-default SADI Restraint Value has been used     0.0050 Report
PLAT191_ALERT_3_G A Non-default SADI Restraint Value has been used     0.0050 Report
PLAT191_ALERT_3_G A Non-default SADI Restraint Value has been used     0.0050 Report
PLAT300_ALERT_4_G Atom Site Occupancy of C44        Constrained at        0.8 Check
PLAT300_ALERT_4_G Atom Site Occupancy of C45        Constrained at        0.8 Check
```

```
PLAT300_ALERT_4_G Atom Site Occupancy of C46        Constrained at       0.8 Check
PLAT300_ALERT_4_G Atom Site Occupancy of C56        Constrained at      0.65 Check
PLAT300_ALERT_4_G Atom Site Occupancy of C57        Constrained at      0.65 Check
PLAT300_ALERT_4_G Atom Site Occupancy of C58        Constrained at      0.65 Check
PLAT300_ALERT_4_G Atom Site Occupancy of C44A       Constrained at       0.2 Check
PLAT300_ALERT_4_G Atom Site Occupancy of C45A       Constrained at       0.2 Check
PLAT300_ALERT_4_G Atom Site Occupancy of C46A       Constrained at       0.2 Check
PLAT300_ALERT_4_G Atom Site Occupancy of C56A       Constrained at      0.35 Check
PLAT300_ALERT_4_G Atom Site Occupancy of C57A       Constrained at      0.35 Check
PLAT300_ALERT_4_G Atom Site Occupancy of C58A       Constrained at      0.35 Check
PLAT300_ALERT_4_G Atom Site Occupancy of H45A       Constrained at       0.8 Check
PLAT300_ALERT_4_G Atom Site Occupancy of H45B       Constrained at       0.8 Check
PLAT300_ALERT_4_G Atom Site Occupancy of H45C       Constrained at       0.8 Check
PLAT300_ALERT_4_G Atom Site Occupancy of H46A       Constrained at       0.8 Check
PLAT300_ALERT_4_G Atom Site Occupancy of H46B       Constrained at       0.8 Check
PLAT300_ALERT_4_G Atom Site Occupancy of H46C       Constrained at       0.8 Check
PLAT300_ALERT_4_G Atom Site Occupancy of H56        Constrained at      0.65 Check
PLAT300_ALERT_4_G Atom Site Occupancy of H57A       Constrained at      0.65 Check
PLAT300_ALERT_4_G Atom Site Occupancy of H57B       Constrained at      0.65 Check
PLAT300_ALERT_4_G Atom Site Occupancy of H57C       Constrained at      0.65 Check
PLAT300_ALERT_4_G Atom Site Occupancy of H58A       Constrained at      0.65 Check
PLAT300_ALERT_4_G Atom Site Occupancy of H58B       Constrained at      0.65 Check
PLAT300_ALERT_4_G Atom Site Occupancy of H58C       Constrained at      0.65 Check
PLAT300_ALERT_4_G Atom Site Occupancy of H45D       Constrained at       0.2 Check
PLAT300_ALERT_4_G Atom Site Occupancy of H45E       Constrained at       0.2 Check
PLAT300_ALERT_4_G Atom Site Occupancy of H45F       Constrained at       0.2 Check
PLAT300_ALERT_4_G Atom Site Occupancy of H46D       Constrained at       0.2 Check
PLAT300_ALERT_4_G Atom Site Occupancy of H46E       Constrained at       0.2 Check
PLAT300_ALERT_4_G Atom Site Occupancy of H46F       Constrained at       0.2 Check
PLAT300_ALERT_4_G Atom Site Occupancy of H56A       Constrained at      0.35 Check
PLAT300_ALERT_4_G Atom Site Occupancy of H57D       Constrained at      0.35 Check
PLAT300_ALERT_4_G Atom Site Occupancy of H57E       Constrained at      0.35 Check
PLAT300_ALERT_4_G Atom Site Occupancy of H57F       Constrained at      0.35 Check
PLAT300_ALERT_4_G Atom Site Occupancy of H58D       Constrained at      0.35 Check
PLAT300_ALERT_4_G Atom Site Occupancy of H58E       Constrained at      0.35 Check
PLAT300_ALERT_4_G Atom Site Occupancy of H58F       Constrained at      0.35 Check
PLAT301_ALERT_3_G Main Residue  Disorder .............(Resd    1)        17% Note
PLAT412_ALERT_2_G Short Intra XH3 .. XHn     H35A     ..H56      .       2.14 Ang.
                                                    x,y,z  =     1_555 Check
PLAT412_ALERT_2_G Short Intra XH3 .. XHn     H44      ..H45F     .       2.06 Ang.
                                                    x,y,z  =     1_555 Check
PLAT412_ALERT_2_G Short Intra XH3 .. XHn     H44      ..H46D     .       1.99 Ang.
                                                    x,y,z  =     1_555 Check
PLAT412_ALERT_2_G Short Intra XH3 .. XHn     H44      ..H46E     .       1.71 Ang.
                                                    x,y,z  =     1_555 Check
PLAT764_ALERT_4_G Overcomplete CIF Bond List Detected (Rep/Expd) .       1.19 Ratio
PLAT860_ALERT_3_G Number of Least-Squares Restraints ............          86 Note
PLAT933_ALERT_2_G Number of HKL-OMIT Records in Embedded .res File          4 Note
               -4  5  4,   4  3  2,   8  0  4,  12  0  1,
```

---

   0 **ALERT level A** = Most likely a serious problem - resolve or explain
   1 **ALERT level B** = A potentially serious problem, consider carefully
  12 **ALERT level C** = Check. Ensure it is not caused by an omission or oversight
  65 **ALERT level G** = General information/check it is not something unexpected

   4 ALERT type 1 CIF construction/syntax error, inconsistent or missing data

```
15 ALERT type 2 Indicator that the structure model may be wrong or deficient
16 ALERT type 3 Indicator that the structure quality may be low
43 ALERT type 4 Improvement, methodology, query or suggestion
 0 ALERT type 5 Informative message, check
```

It is advisable to attempt to resolve as many as possible of the alerts in all categories. Often the minor alerts point to easily fixed oversights, errors and omissions in your CIF or refinement strategy, so attention to these fine details can be worthwhile. In order to resolve some of the more serious problems it may be necessary to carry out additional measurements or structure refinements. However, the purpose of your study may justify the reported deviations and the more serious of these should normally be commented upon in the discussion or experimental section of a paper or in the "special_details" fields of the CIF. checkCIF was carefully designed to identify outliers and unusual parameters, but every test has its limitations and alerts that are not important in a particular case may appear. Conversely, the absence of alerts does not guarantee there are no aspects of the results needing attention. It is up to the individual to critically assess their own results and, if necessary, seek expert advice.

Publication of your CIF in IUCr journals

A basic structural check has been run on your CIF. These basic checks will be run on all CIFs submitted for publication in IUCr journals (*Acta Crystallographica*, *Journal of Applied Crystallography*, *Journal of Synchrotron Radiation*); however, if you intend to submit to *Acta Crystallographica Section C* or *E* or *IUCrData*, you should make sure that full publication checks are run on the final version of your CIF prior to submission.

Publication of your CIF in other journals

Please refer to the *Notes for Authors* of the relevant journal for any special instructions relating to CIF submission.

PLATON version of 02/02/2025; check.def file version of 02/02/2025

Datablock y25msh32 - ellipsoid plot

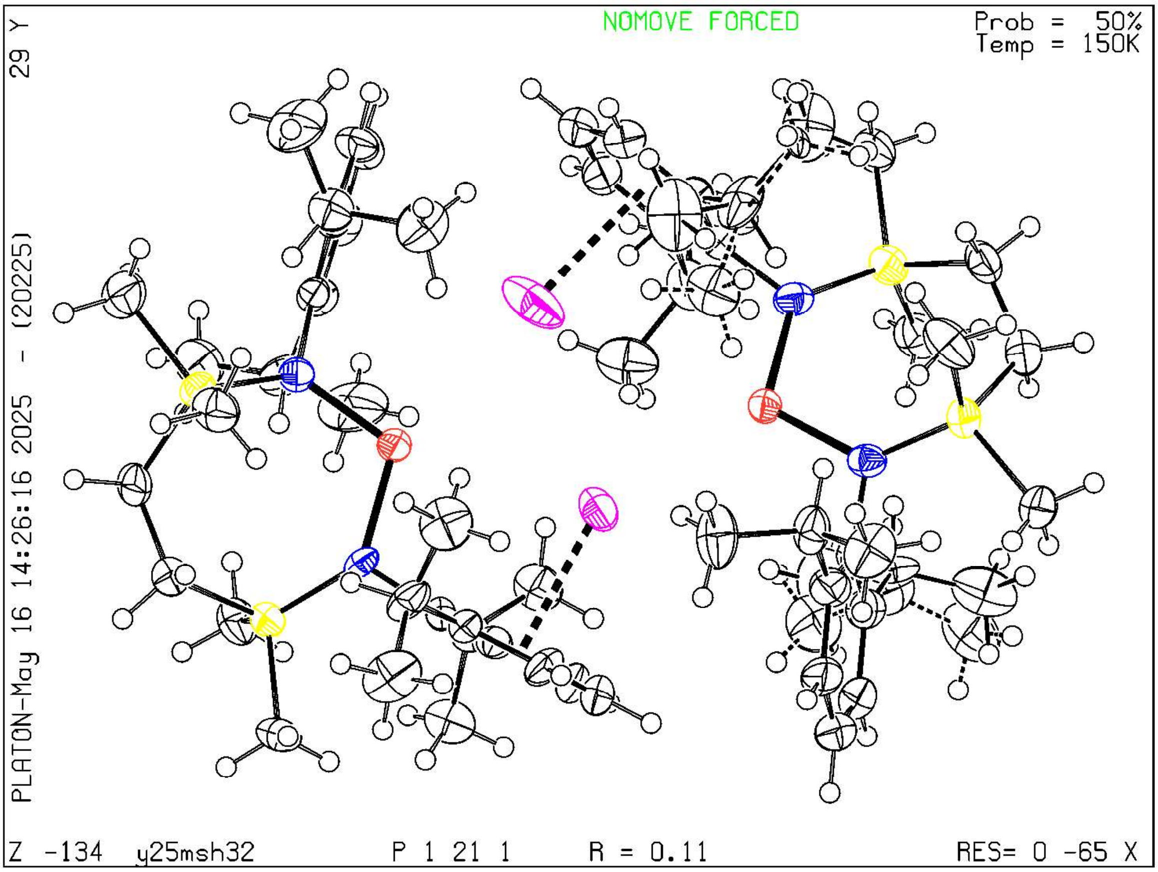

Supplement: Supplementary file 2 — Supporting Information [file CHEM-31-e202502197-s002.pdf]
